# Machine learning-based prediction of drug response in ischemia reperfusion animal model

**DOI:** 10.1038/s41598-025-18620-8

**Published:** 2025-12-02

**Authors:** Asmaa Mohamed Abd ElGwad, Ibrahim Youssef, Abdelrahman Khaled, Eman K. Habib, Nashwa Naguib Omar, Heba F. Khader, Seham Saleh Alaiyed, Mansour Altayyar, Basma Emad Aboulhoda, Marwa Matboli

**Affiliations:** 1 Medical Biochemistry and Molecular Biology Department, Faculty of Medicine, Ain Shams University, Cairo, Egypt; 2 Systems and Biomedical Engineering Department, Faculty of Engineering, Cairo University, Cairo, Egypt; 3 Bioinformatics Group, Center of Informatics Sciences (CIS), School of Information Technology and Computer Sciences, Nile University, Giza, Egypt; 4 Department of Anatomy and Embryology, Faculty of Medicine, Galala University, Ataka, Egypt; 5 Department of Anatomy and Embryology, Faculty of Medicine, Ain Shams University, Cairo, Egypt; 6 Clinical and Chemical Pathology Department, Faculty of Medicine, Ain Shams University Hospitals, Cairo, Egypt; 7 Department of Medical Biochemistry, Menoufia Faculty of Medicine, Menoufia University, Shebin Al-Kom, Egypt; 8 Department of Physiology, College of Medicine, Qassim University, Buraidah, Saudi Arabia; 9 Department of Basic Medical Sciences, College of Medicine, University of Jeddah, 23890 Jeddah, Saudi Arabia; 10 Anatomy and Embryology Department, Faculty of Medicine, Cairo University, Cairo, Egypt; 11 Molecular biology research lab, Faculty of oral and dental medicine, Misr International University, Cairo, Egypt

**Keywords:** Myocardial Ischemia-Reperfusion (MI/R) injury, Cardioprotection, Epigenetic regulation, Anti-inflammatory therapy, Dose-dependent effects, Machine learning, Computational biology and bioinformatics, Drug discovery, Biomarkers, Cardiology, Diseases

## Abstract

Myocardial ischemia is a major global contributor to mortality. While reperfusion therapy remains the most effective treatment, it paradoxically leads to myocardial ischemia–reperfusion (MI/R) injury, resulting in irreversible cardiac damage for which no effective interventions currently exist. This underscores the pressing need to unravel the pathogenesis of MI/R injury and devise new therapeutic strategies. In this study, supervised machine learning models, including logistic regression (LR), support vector machines (SVM), random forests (RF), neural networks (NN), and k-nearest neighbors (kNN), were utilized to predict treatment response. The models incorporated molecular and biochemical features to evaluate three drugs: trans-Anethole (TNA), pentoxifylline (PTX), and cyanidin-3-O-glucoside (Cy3G). The sequential forward selection (SFS) method was employed to select the most relevant features for prediction. To assess model performance, metrics such as precision, accuracy, recall (sensitivity), specificity, and the Matthews Correlation Coefficient (MCC) were analyzed for both reduced and complete models. Among the classifiers, kNN demonstrated notable performance, achieving an accuracy of 0.9156 ± 0.0242 and an average area under the ROC curve (AUC) of 0.90 across three cross-validation iterations surpassing all other classifiers. This observed performance is in line with recent literature that employs advanced computational methods in similar domains. A key advantage of our study is the use of a two-layer framework—integrating molecular signatures with biochemical markers—which can provide improved robustness and biological relevance. This multi-layer integration enhances interpretability and better reflects the multifactorial nature of MI/R injury, while supporting model generalization. Feature selection identified one molecular marker (SOX5) and two biochemical markers (dP/dtmax and cTnT) as significant predictors of drug response. This integrative approach has the potential to enhance personalized therapy for myocardial ischemia by enabling precise drug response predictions and guiding the development of targeted treatment strategies.

**Supplementary Information:**

The online version contains supplementary material available at 10.1038/s41598-025-18620-8.

## Introduction

Ischemic heart disease (IHD) is the most widespread cardiovascular disease globally, with 9.21 million deaths in 2021^1^. Even in developed countries, IHD remains the leading cause of health burden and mortality^2^.

Myocardial reperfusion is one of the most widely adopted therapeutic strategies to address injury following acute myocardial infarction (AMI) by restoring cardiac performance. However, it may exacerbate injury via a process known as ischemia–reperfusion (I/R) injury. Numerous pathways are engaged in I/R injury, including inflammation, oxidation, apoptosis, autophagy, necrosis, ferroptosis, extracellular matrix remodeling, and fibrosis^3–5^. Significant cell death can initiate an inflammatory process and the release of inflammatory mediators known as cytokines. Damage-associated molecular patterns (DAMPs) released from dead cells activate MI/R injury through the initiation of Nuclear Factor Kappa B (NFκB) expression, which, in turn, induces the expression of the NOD-LRR-and Pyrin Domain-Containing Protein 3 (NLRP3) inflammasome in the heart. NLRP3 enhances the infarction process and promotes more myocardial cell death after reperfusion by increasing inflammation and Interleukin-1 Beta (IL-1β) secretion^6^. Additionally, Transforming Growth Factor Beta (TGF-β) is a leading player in myocardial fibrosis. TGF-β also increases the release of cytokines, which is considered the root cause of MI/R injury and represents a major obstacle to treatment^7,8^. Most current treatments focus on minimizing mortality; however, they fail to heal fibrosis or prevent chronic cardiac failure. Consequently, this has created an important research focus on restoring cardiomyocytes affected by MI/R injury^9^.

The increasing advancement in high-throughput sequencing technologies has provided comprehensive insights into the pathology of diseases by analyzing genetic and epigenetic information^10^. Epigenetic modifiers, such as non-coding RNAs (such as long non-coding RNAs (lncRNAs) and microRNAs (miRNAs), have a crucial impact on cardiac reconstruction following MI/R injury^3,11–13^. They enhance reactive oxygen species (ROS) production, oxidative stress, inflammation, and subsequent cell death pathways. Furthermore, lncRNAs activate and/or sponge several miRNAs involved in oxidative stress, inflammation, and cell death pathways^14–16^. For instance, LncRNA NORD promotes apoptosis and fibrosis by inhibiting miR-577 in a rat model. LncRNA MALAT1 induces fibrosis and worsens heart function following MI. Additionally, miRNA-21 exacerbates MI/R injury by promoting cardiac fibrosis through the TGF-β/Smad7 pathway^17^, while miRNA-22 inhibits cAMP response element-binding protein (CBP), leading to suppression of p21 and Bcl-2-Associated X (BAX) gene expression and thereby inhibiting apoptosis caused by MI/R injury^18^.

Most current treatments focus on minimizing mortality; however, they fail to heal fibrosis or prevent chronic cardiac failure. Consequently, this has created an important research focus on restoring cardiomyocytes affected by MI/R injury^9^. Thus, a key goal of medical research is to elucidate strategies for safeguarding the myocardium following MI/R injury^5^. This study, conducted on a rat model, aimed to explore a potential drug for the treatment of MI/R injury. As inflammation is crucial for the development of MI/R injury, this work investigated three drugs with anti-inflammatory properties on MI/R injury; two phytochemical trans-anethole (TNA) and cyanidin-3-O-glucoside (Cy3G) versus synthetic drug pentoxifylline (PTX). TNA is a naturally occurring material found in the essential oils of fennel, anise, and star anise, which exhibits anti-inflammatory properties and antioxidant^19,20^. It also demonstrates anticancer effects by hindering cell proliferation, enhancing apoptosis, and regulating the Phosphoinositide 3-Kinase/Protein Kinase B/Mechanistic Target of Rapamycin (PI3K/Akt/mTOR), NF-κB, and Mitogen-Activated Protein Kinase (MAPK) pathways^21^. Similarly, a methylxanthine derivative called PTX is known to affect blood viscosity and enhance microcirculation. It also has anti-inflammatory effects, reducing IL-6, TNF-α, and CRP levels^1,22^. PTX enhances the expression of AKT and PI3K anti-apoptotic genes and boosts antioxidant enzymes, including superoxide dismutase (SOD) and catalase. Conversely, it decreases the oxidative stress marker malondialdehyde (MDA) and the apoptotic marker caspase^23^. Cy3G, a natural anthocyanin, decreases the expression of IL-1β , IL-6, and TNF-α cytokines^24^. It also exhibits potent antioxidant activity by stimulating Nrf2 antioxidant protein expression via the MAPK/ Extracellular Signal-Regulated Kinase (ERK) pathway^25^.

MI/R injury is a multifaceted process involving numerous factors and interactions at genetic, epigenetic, and cellular levels. A complete understanding of the mechanisms underlying MI/R injury may offer unique therapeutic targets and improve outcomes. While cardiac markers are valuable tools, their use in follow-up and monitoring is limited by issues of sensitivity, specificity, prognostic value, and variability^26^. While cardiac markers are valuable tools, their use in follow-up and monitoring is limited by issues of sensitivity, specificity, prognostic value, and variability^27,28^. While prior studies have explored individual drugs or biomarkers for MI/R injury, no study has combined molecular signatures (e.g., SOX5, miR-133a-3p) with functional hemodynamic markers (e.g., dP/dtmax, cTnT) in an ML-driven framework for treatment response prediction. Our work bridges this gap by offering a scalable approach to personalize cardioprotective therapy. MI/R injury, as well as for improving prognosis and treatment.

Hence, there is an urgent need to employ a machine learning for a better understanding of the key players engaged in MI/R injury, as well as for improving prognosis and treatment^29^. To address this need, we leverage a machine learning approach to predict therapeutic response to three promising MI/R drugs (TNA, PTX, Cy3G) in a preclinical model. Our approach uniquely offers several contributions to the field of MI/R injury research. Specifically, we integrate molecular and biochemical features across normal, diseased, and treated animal groups (n = 176) to capture a more comprehensive representation of drug response compared to conventional single-layer biomarker approaches. By applying Sequential Forward Selection (SFS), we derive minimal yet highly informative biomarker panels that improve interpretability while reducing model complexity. Furthermore, we evaluated five distinct classifiers (KNN, Logistic regression, Random Forest, SVM, and Neural Networks) to benchmark predictive performance and identify robust models suited for translational applications. Collectively, this study highlights the value of a multi-layer ML framework for enhancing robustness, biological relevance, and interpretability in drug response prediction within the context of MI/R injury.

In parallel with our contribution, recent advances in computational biology have demonstrated the potential of specialized ML and DL frameworks in addressing complex biomedical prediction problems. For instance, models such as DeepAIPs-Pred (anti-inflammatory peptide prediction)^30^, pACP-HybDeep (anticancer peptide identification)^31^, PNPS-CapsNet (neuropeptide prediction)^32^, as well as DeepSynergy and TransSynergy for drug synergy prediction^33^. These frameworks exemplify how capsule networks and deep learning designs deliver both high predictive accuracy and biological insight. While our study differs in scope by focusing on MI/R injury and prioritizing interpretability through a multi-layer, feature-optimized ML design, acknowledging these advances situates our work within the rapidly evolving landscape of computational approaches in translational biomedical research.

## Material and methods

This work attempts to generate a machine learning model to predict response to trans-Anethole (TNA), Pentoxifylline (PTX), and cyanidin-3-O-glucoside (Cy3G) in the treatment of MI/R injury in the rat model.

### Drugs and chemicals

TNA, PTX, urethane, Cy3G and pentobarbital sodiumwere bought from (Sigma Aldrich, St. Louis, USA) Supplementary table 1.

### Animal model

One hundred seventy-eight male Wistar rats, weighing between two hundred and two hundred fifty grams, were acquired from the Nile Pharmaceuticals company’s animal house (Cairo, Egypt). Rats were housed in an animal room with a twelve-hour dark/light cycle. Normal rat chow ad libitum and tap water were available. The National Institutes of Health’s instructions to deal with laboratory animals were followed. (NIH Publication No. 85–23, revised 1996), and received approval from the Faculty of Medicine’s Institutional Animal Ethics Committee, Ain Shams University. Approval no. FWA000017585, FWA0000149. All methods were performed in accordance with Ain Shams University Animal Ethics Committee guidelines and regulations and in accordance with ARRIVE guidelines. Euthanasia was carried out via anesthetic overdose followed by exsanguination to be compatible with surgical models requiring tissue preservation ( heart for infarct size measurement) and minimizing pain during the procedure. We confirmed anesthesia depth via verifying loss of pedal reflex (toe pinch) and stable respiratory depression (< 30 breaths/min).Exsanguination was carried out via Cardiac puncture and collecting blood for 2–3 min until circulatory collapse occurs, followed by secondary death confirmation.

### MI/R injury initiation

1.5 mg/kg body weight of urethane has been administered to anesthetize rats ^34^. Intubation was done using a rodent ventilator adjusted at 70–80 breaths per minute. After that, the heart was exposed via median sternotomy, with sutures encircling the left anterior descending coronary artery utilizing 5–0 polyethylene suture. The free ends of the suture were used to create a noose around a rubber band that was flattened on the myocardium. The noose was tightened around the rubber band for forty-five minutes, which caused coronary occlusion^35^. The infarcted area instantly blanched, proving this. Reperfusion was attained by releasing the knot for 120 min. An electrocardiogram investigation in lead II was done both before and following ligation to establish MI injury. A similar process was applied to the sham animals, but they were not sutured^36^.

### Animal groups

**Fig. 1:**
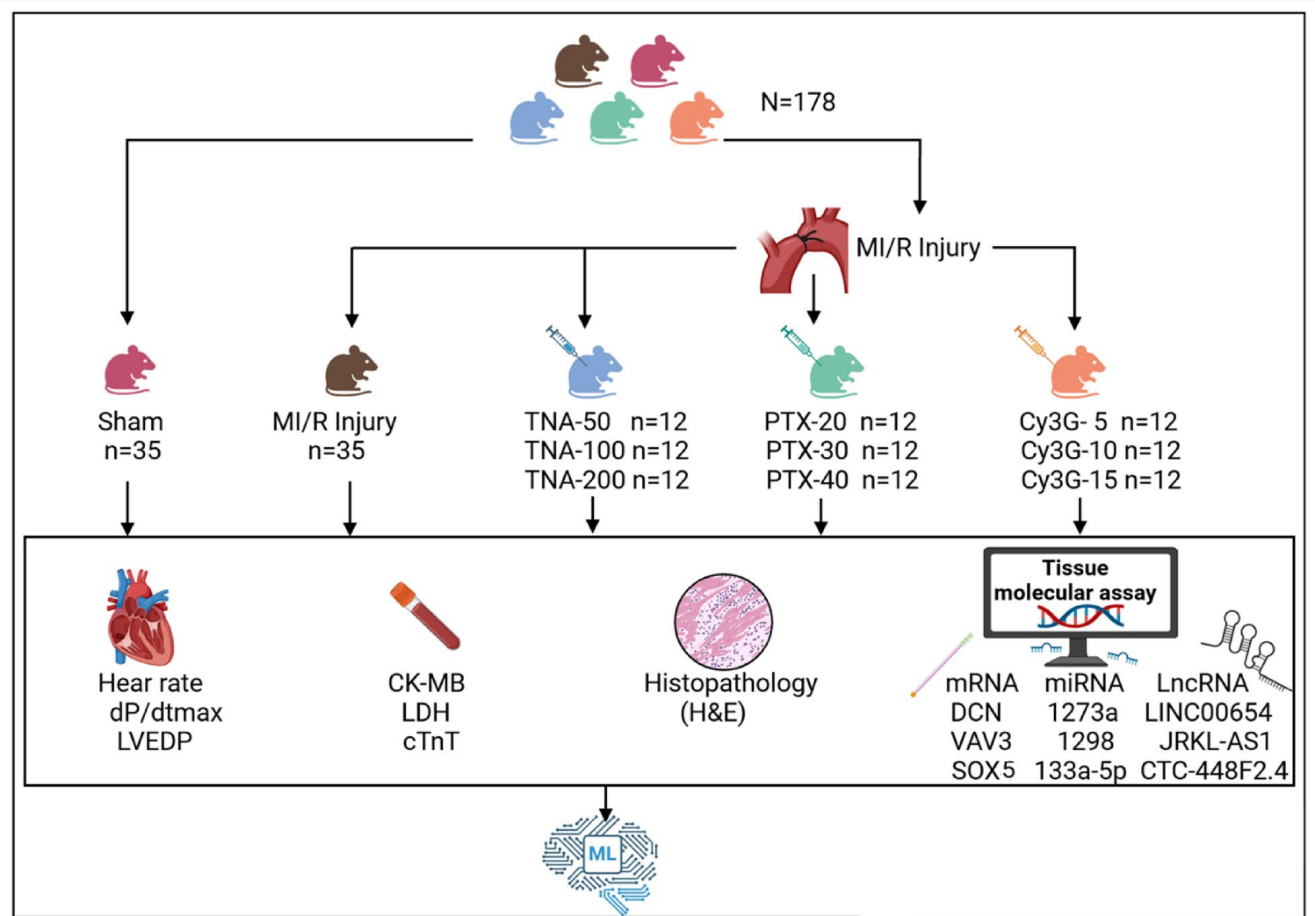
Graphical Abstract of the study Design, including the sham control group, the myocardial injury reperfusion group which is subdivided into the untreated group, the trans-Anethole (TNA) 50, 100 & 200 treated groups, the pentoxifylline (PTX) 20, 30 & 40 treated groups, and the cyanidin-3-O-glucoside (Cy3G) 5, 10 & 15 treated groups. All groups underwent heart performance assessment, including heart rate, maximum rate of pressure change (dP/dtmax), and left ventricular end-diastolic pressure (LVEDP). Cardiac markers Creatine Kinase-MB (CK-MB), Cardiac Troponin T (cTnT), and Lactate Dehydrogenase (LDH) were assessed in blood. Also, histological examination of the heart tissues was done by hematoxylin–eosin (H&E) and bioinformatic to retrieve mRNAs of DEG genes decorin (DCN), Vav guanine nucleotide exchange factor 3 (VAV3), SRY-box transcription factor 5 (SOX5) and their epigenetic regulators miRNAs (miR-1273a, miR-1298 and miR-133a-3p and their interacting lncRNAs (long intergenic non-protein coding RNA 654 (LINC00654), JRKL antisense RNA 1 (JRKL-AS1) and CTC-448F2.4. Finally, all this data was analyzed using machine learning algorithms.This figure was created with BioRender.com (https://biorender.com/) under a CC BY open access license.

Rats were allocated at random into 11 groups: sham group, MI/R injury group, both including 35 rats, TNA-50 group, TNA -100 group, TNA-200 group, PTX-20 group, PTX-30 group, PTX-40 group, Cy3G-5 group, Cy3G-10 group, and Cy3G-15 group, all treated groups included 12 rats. Intraperitoneal injections of drug dosages were performed five minutes before coronary sutures. Heart performance, markers, histopathological examination, and molecular assay (Fig. 1).

### Heart performance assessment

After delicately pulling off the pericardium over the heart with forceps, a 25–30-gauge needle was used to make a stab wound near the apex of the heart. Next, after removing the needle, a pressure catheter tip (Millar Mikro-Tip®; paired with a PowerLab data acquisition system; NIBP, AD Instruments, Australia) was inserted into the left ventricle. After ten minutes of signal stabilization, heart rate, maximum rate of pressure change (dP/dtmax), and left ventricular end-diastolic pressure (LVEDP) were evaluated. Each reading took three to five minutes, and the mean of three repeated measurements was computed. The catheter was then carefully withdrawn from the stab wound^37^.

### Cardiac marker evaluation in the blood sample

Creatine Kinase-MB (CK-MB), Cardiac Troponin T (cTnT), and Lactate Dehydrogenase (LDH) cardiac markers were examined in sera of retroorbital blood samples after their centrifugation at 1200 × g for 10 min.A spectrophotometer was used for the assessment of serum LDH. Commercial ELISA kits were used for measuring serum cTnT and CK-MB as per the manufacturer’s guidance^38^.

### Histological examination of myocardium

Following ful- night fixation in PBS-buffered formalin 10% both ventricular myocardial tissues were kept in 70% ethanol before being embedded in a paraffin block. The 3 μm tissue slices underwent standard hematoxylin–eosin (H&E)^39^ and Masson’s trichrome staining to identify collagen fibers. All tissue sections were inspected for signs of fibrosis and dying cells^40^. Slides were coded by an independent researcher not involved in the scoring process. Histopathologists evaluating the sections (e.g., necrosis, edema, hemorrhage) were unaware of group assignments (sham, MI/R, or treatment groups). Scores were assigned based on standardized morphometric thresholds (e.g., area percentage of edema measured via Leica QWin V3 software). Inter-observer validation was performed between two pathologists; discrepancies were resolved by consensus.

Each examined cardiac muscle section received a score from 0 to 3, according to criteria typically outlining the severity and distribution of pathological changes based on the following indicators: cell necrosis, presence of polymorphonuclear leucocytes, loss of cross striation, edema and microscopic hemorrhage^41^. Edema and hemorrhage were estimated through measuring area percentage of the examined fields using Leica QWin V3 (Quantitative Windows Image Analysis Software) Version: 3.0.1 (Leica microsystems, Germany)( URL link ; https://www.leica-microsystems.com/ ) at image analysis unit, Histology and Cell Biology Department, Faculty of Medicine, Ain Shams University. This was done through two steps: step I; slide grading through examining 4 different high-power fields of H&E-stained sections. step II; group grading by examining 10 different slides were for each group (e.g., in a group of 10 slides, if 4 slides scored 1, 3 slides scored 0, 2 slides scored 3 and 1 slide scored 2, then the score for this group will be 1) supplementary table 2. ImageJ v1.53 (NIH) with *Color Deconvolution Plugin* to quantify collagen-specific staining (blue channel). Fibrotic Area (%): Total collagen-positive area ÷ total tissue area per field. Fibrotic Density: Mean blue-channel intensity in fibrotic regions (0–255 scale). Table S3A–B shows fibrosis metrics across experimental conditions.

### Bioinformatic analysis

**Fig. 2:**
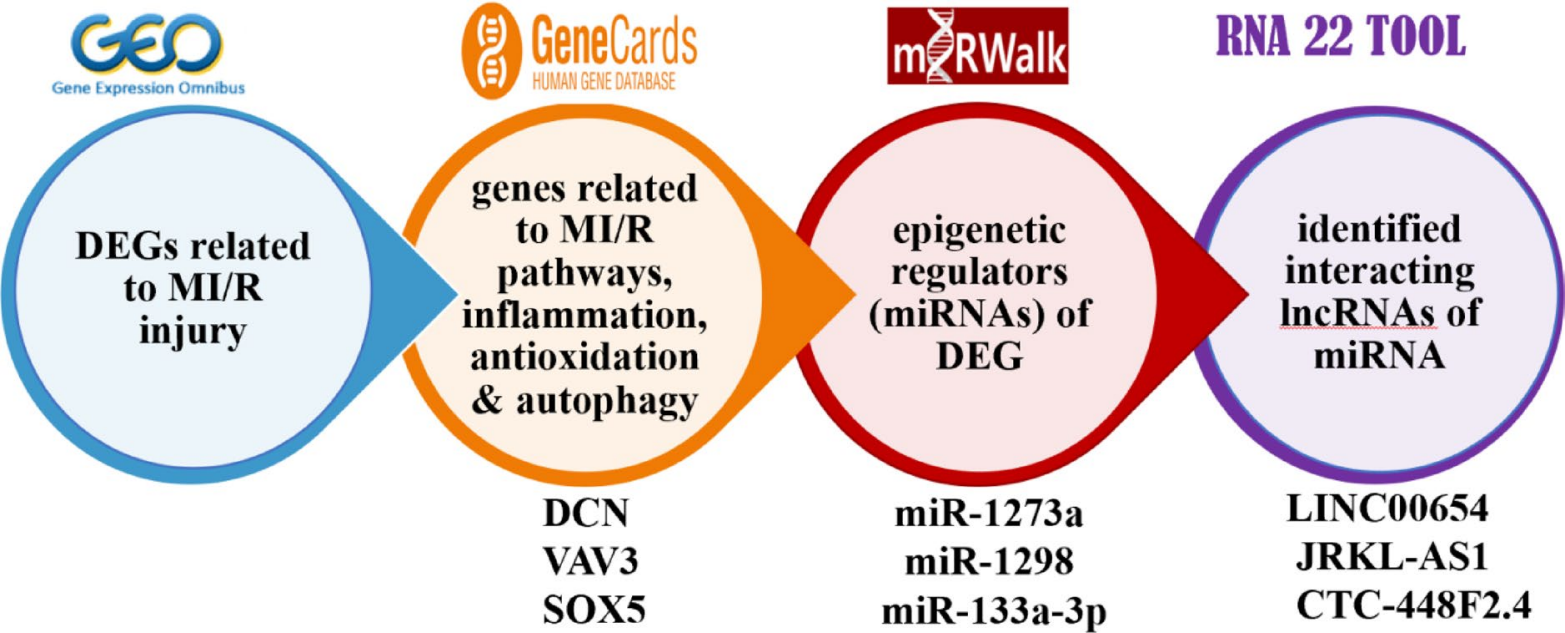
Rationale for selection of the genes involved in MI/R pathogenesis: the Gene Expression Omnibus (GEO) database was used to select DEGs in MI/R injury. The Genecards database was utilized to genes related to retrieve inflammation & autophagy (DCN, VAV3 & SOX5) among DEGs. The miRWalk database was used to identify epigenetic regulators of these DEG; miR-1273a, miR-1298 and miR-133a-3p were chosen. The RNA22 tool was used to select interacting lncRNAs (long intergenic non-protein coding RNA 654 (LINC00654), JRKL Antisense RNA 1 (JRKL-AS1) and CTC-448F2.4 using the RNA22 on selected miRNAs.

In the current study, a depth investigation assessment of the gene involved in MI/R injury was done. As in Fig. 2, The Gene Expression Omnibus (Home—GEO DataSets—NCBI ) database^42^was utilized to obtain mRNA data associated with MI/R injury, using the search term “Ischemia Reperfusion.” The inclusion criteria were expression profiling performed via array, samples collected from both ischemia/reperfusion (I/R) vs controls, and datasets comprising more than five samples. Based on these criteria, two datasets were selected: GSE61592 and GSE14843. Differentially expressed genes (DEGs) were identified using the GEO2R/R package limma with thresholds set at a p-value < 0.05 and |logFC|> 0.5 (Supplementary Tables S4). Gene ontology analysis was conducted using the GeneCards—Human Genes | Gene Database | Gene Search^43^, focusing on genes related to autophagy and inflammation, which are strongly associated with I/R pathogenesis. Thus, Decorin (DCN), Vav guanine nucleotide exchange factor 3 (VAV3), and SRY-box transcription factor 5 (SOX5) were selected^44–47^ (Fig. S1). Our in silico rationale for the chosen genes went in hand with established literature. Inflammation and autophagy are core drivers of MI/R injury pathogenesis, as evidenced by: DAMPs → NF-κB → NLRP3 lead to inflammasome activation amplifies IL-1β–mediated cardiomyocyte death (Toldo & Abbate, 2018). Impaired autophagic flux exacerbates oxidative stress and apoptosis in reperfused myocardium (Matsui et al., 2007). Both pathways are recognized therapeutic targets for cardioprotection. autophagy/inflammation genes were prioritized due to their established mechanistic roles in MI/R injury^48,49^. Thus, Decorin (DCN), Vav guanine nucleotide exchange factor 3 (VAV3), and SRY-box transcription factor 5 (SOX5) were selected . To identify epigenetic regulators of these DEGs, we first retrieved miRNAs interacting with the selected DEGs (miR-1273a, miR-1298 and miR-133a-3p) using the Home—miRWalk database^50^ (Fig. S2). miRWalk: Validated Multi-Source Integration aggregates predictions from 12tools (e.g., TargetScan, miRanda) and experimentally validated interactions across databases (miRTarBase, TarBase). It covers both mRNA and miRNA targets with high confidence. It also filtered false positives via CLIP-seq validation data. It applies machine learning approaches such as random forest approach (TarPmiR) to improve accuracy and provide comprehensive, up-to-date interaction data. Finally, we used the RNA22 tool Computational Medicine Center at Thomas Jefferson University | Big Data in Precision Medicine, Novel Molecules & Disease Research^51^ to identify interacting lncRNAs (long intergenic non-protein coding RNA 654 (LINC00654), JRKL Antisense RNA 1 (JRKL-AS1) and CTC-448F2.4 using the RNA22 tool (Fig. S3)^52^. RNA22: De Novo Motif Discovery for Non-Coding RNAs uses sequence-based pattern recognition (independent of conservation) to detect non-canonical binding sites in lncRNAs. It predicts miRNA-lncRNA interactions via structural stability (ΔG ≤  − 15 kcal/mol). It uses a pattern-based approach to identify potential binding sites and validate them through experimental assays, demonstrating high specificity and the ability to extrapolate to novel microRNAs without prior sequence similarity. Default parameters were applied (e.g. binding scores, seed match, etc.) was shown in (Table S5A–B). This integrated approach(integrative prediction and thermodynamics-based validation) strengthened our epigenetic regulatory network analysis and together enhancing the reliability of miRNA target identification. (Figure S3). Enhanced mechanistic insights (e.g., lncRNA H19 sponging miR-455-5p to regulate autophagy genes).

### RNA extraction from cardiac tissues

The miRNEasy® RNA purification kit (Qiagen, Cat no. 74104) was utilized to purify and isolate total RNA, as well as miRNA, from cardiac tissues following the company’s directions. Quantity and the quality of RNA were examined using the DeNovix DS-11 microvolume spectrophotometer (Wilmington, USA); RNA concentrations ranged from 1.8 to 2. Before the first strand of complementary DNA was generated, DNase 1 was applied to the RNA. QIAGEN OneStep RT-PCR Kit (Qiagen, Cat no. 210212) was used to reverse transcribe the total RNA into cDNA. A real time PCR Applied Biosystems 7500 v2.3 (Applied Biosystems, Foster City, USA) heat cycler was used to conduct reverse transcription quantitative polymerase chain reaction (RT-qPCR).

Differential expression evaluation of DCN, VAV3 and SOX5 mRNAs was performed utilizing “QuantiTect SYBR Green Master Mix, Cat no. 204143” by Qiagen and QuantiTect Primer Assays NM_001920, NM_001079874 and NM_00 6940 utilizing GABDH NM_023964 as an endogenous control following the manufacturer’s instructions. Meanwhile, the "miScript SYBR Green PCR Kit, Cat no. 218073" from Qiagen and the miScript LNA primer assays were used to examine the differential expression of miR-1273a, miR-1298 and miR-133a-3p GeneGlobe IDs YP02106480, YP02108491 and YP00204788, using U6 as an internal control. U6 was selected based on prior evidence of stability in cardiac tissue under ischemic/reperfusion conditions12. Our data further supports its reliability for miRNA normalization in this study. U6 expression stability was rigorously validated across all experimental groups (sham, MI/R injury, and all drug-treated groups). One-way ANOVA confirmed no statistically significant differences in U6 Ct values between groups (P > 0.05). U6 Ct values consistently fell within a narrow range (± 0.5 cycles) across all samples, indicating minimal technical variability (Figure S4). RT2 SYBR Green ROX qPCR Master mix (Qiagen, Helman Germany; Cat no:330500) used for evaluation of differential expression of LINC00654, JRKL-AS1 and CTC-448F2.4 LncRNAs using RT^2^ lncRNA qPCR primers Assay GeneGlobe ID: LPH02280A, LPH20063A and LPH25249A respectively, utilizing GABDH NM_023964 as an endogenous control. The setting of the PCR was as follows: initial heating for 15 min at 95 °C, then 40 cycles of 94°C for 15 s, 55°C for 30 s, and finally 72 °C for 30 s. The Rotor-Gene real-time PCR detection system (Qiagen, Düsseldorf, Germany) was used to determine each sample’s threshold cycle (Ct) value. More than 35 was regarded as a negative Ct value. Using the Leviak method, where RQ = 2^−ΔΔCt^, the (RQ) relative quantification of RNA expression was calculated^53^.

### Statistical analysis

Measurements are represented as mean ± SD. A one-way ANOVA test subsequently followed by Tuckey’s test to compare significance with the sham and MI/R injury groups^54^. The Shapiro–Wilk test was utilized to assess normality. Statistical analysis was done using JASP 0.19.0.0.

### Binary classification of treatment response

A major purpose of this study is to build a computational model to infer the treatment response through constructing a machine learning predictive model that utilizes a set of observational measurements summarizing the sample post-treatment status. Let ${X}_{i}\in {R}_{+}^{n\times {p}_{i}}$ represent a specific category of measurements, where *i* ∈ {Molecular, Biochemical} (Table 1), ${R}_{+}$ denotes non-negative real numbers, *n* is the number of samples (Table 2), and *p*_*i*_ is the number of features within category *i* (Table 1); and let $Y\in {N}^{n\times 1}$ denote the treatment response, where $N$ represents the non-negative integers. Response $Y$ is a binary variable summarizing the treatment response and depends on the damage score (*DS*) classification (responsive/non-responsive) is inherently histology-driven, as DS thresholds (0–4) were derived from standardized morphometric analysis (H&E/Masson’s trichrome).. *DS* ∈ {0 (no damage), 1 (mild), 2 (moderate), 3 (severe), 4 (highly severe)}. A *DS* value $\le 2$ is classified as a successful treatment. Based on this classification, a sample’s responsiveness is defined as a binary variable (1 for responsive, 0 for unresponsive), using the following function:

$$Y = \left\{ {\begin{array}{*{20}l} {1,} \hfill & {{\text{if}}\;DS \le {2}.} \hfill \\ {0,} \hfill & {{\text{if}}\;DS > { 2}.} \hfill \\ \end{array} } \right.$$

**Table 1:** Features and their count for each data type.

Molecular (9 features)	Biochemical (6 features)
1. DCN	1. LVEDP
2. VAV3	2. dP/dtmax
3. SOX5	3. Heart Rate
4. miR-1273a	4. cTnT
5. miR-1298	5. CK-MB
6. miR-133a-3p	6. LDH
7. LINC00654	
8. JRKL-AS1	
9. CTC-448F2.4	

**Table 2:** Number of samples for each group.

Condition	Number of samples
Control (healthy)	35
IRH (disease model; untreated)	35
TNA-50	12
TNA-100	12
TNA-200	12
PTX-20	12
PTX-30	12
PTX-40	12
Cy3G-5	12
Cy3G-10	12
Cy3G-15	12

### Predictive modeling of treatment response

We built several supervised machine learning models to predict the treatment response. These models differ in the processed data type and used classifier. Three different groups of the collected features were processed per model: molecular, biochemical, and their combination. In addition, five various classification techniques were employed, and support vector machines (SVM), random forests (RF), logistic regression (LR), neural networks (NN), and k-nearest neighbors (kNN). To summarize, three models were tested for each classifier to represent the different feature types of data. This exhaustive, combinatorial pattern for the selection of the model predictors out from the different observed features can help with gauging the differential predictive power of the above data types. Besides the aforementioned three constructed models per classifier, we constructed another three corresponding models, which we designated as “reduced” models. In each of these reduced models, we aimed at selecting the smallest set of the most informative features that can best predict the measured outcome. We made use of the greedy forward sequential feature selection (SFS) approach with random forests as the estimator to reduce the model complexity and computational power needed and increase the model interpretability by reducing the number of features included in each predictive model.

The classification techniques used in this study were configured with the following parameters. The kNN classifier was set to 5 neighbors with uniform weighting. The RF classifier consisted of 100 trees, each with a maximum depth of 10. For the NN classification, a multi-layer perceptron (MLP) was employed, featuring two hidden layers with 6 and 4 neurons, respectively, to minimize overfitting; the solver used was the “lbfgs” optimizer. The SVM classifier had a regularization parameter (C) value of 1, and the stopping criterion tolerance was set to 0.0001. The LR classifier allowed a maximum of 100,000 iterations to achieve convergence, with a stopping tolerance of 0.0001. Additionally, the SFS method utilized an RF estimator in the forward direction, incorporating a stopping criterion tolerance of 0.00001 and performing cross-validation across 5 iterations. Table 3 summarizes the tuned parameters for the different prediction models used in this study.

**Table 3:** Tuned parameters for the prediction models.

Classifier	Parameter	Value
RF	# trees	100
	Maximum depth per tree	10
kNN	k	5
	Weight	Uniform
NN	Type	MLP
	# Hidden layers	2
	# Neurons per hidden layer	(6, 4)
	Solver	“lbfgs” optimizer
SVM	Regularization parameter (C)	1
	Stopping tolerance	0.0001
LR	# Iterations (max)	100,000
	Stopping tolerance	0.0001
SFS	Estimator	RF
	Direction	Forward
	Stopping tolerance	0.00001
	Cross validation	5 Iterations

### K-fold cross validation and SMOTE

In order to test the model predictive performance, we split data into two disjoint, exclusive sets, where the first set was employed as the training samples for the machine learning model and the second set was treated as the unseen samples that were fed to the trained model to test its predictive performance. Such splitting of the data samples avoids model overfitting and increases the model generalizability to predict the outcome for data samples not involved in the training phase. This splitting process was repeated *k* times to perform a *k*-fold cross validation (kCV) assessment, where every single sample was used once in the testing phase. The dataset for this study includes samples representing the control group, other samples representing the disease model (no treatment), and a third group of samples exemplifying the different treatment drugs. In order to reduce the misclassification rate which might result from under-representing a group of samples, we opted for an approach capable of preserving the same stratified sampling pattern at each CV iteration instead of following the leave-one-group-out strategy. Each group was divided into *k* subsets, ensuring that during each cross-validation (CV) iteration, only one subset was designated for testing while the remaining subsets were utilized for training. Let *s*_*i*_ represent the index of sample *s*, where *i* = {1, 2, 3, …, *n*} and *n* is the total number of study samples. The function *mod*(*s*_*i*_,*k*), which employs the modulus operator, calculates the integer remainder of dividing *s*_*i*_ by *k .* The output of (*mod*(*s*_*i*_,*k*) + 1) falls within {1, 2, 3, …, *k*}. During the *k*^th^ CV iteration, samples for which (*mod*(*s*_*i*_,*k*) + 1) = *k* were selected as the test set, while the remaining samples were used for training the model.

**Fig. 3:**
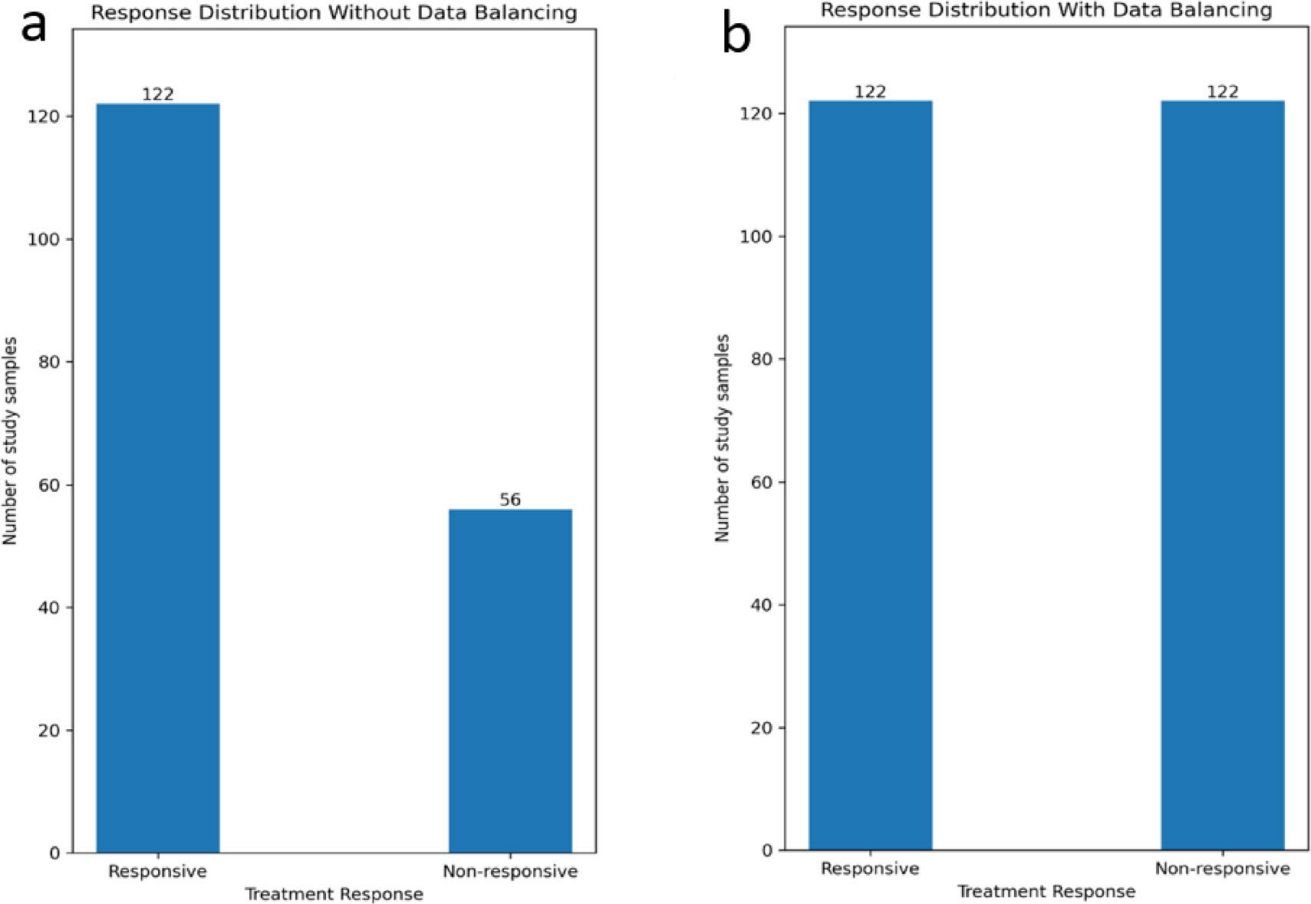
The number of responsive and non-responsive samples (**a**) before and (**b**) after applying the SMOTE technique.

In this study, the two classes representing treatment response were labeled as responsive and non-responsive, with a ratio of 1:2.18, as illustrated in Fig. 3a, b. To address the imbalance and reduce classifier bias, the Synthetic Minority Oversampling Technique (SMOTE) was applied to upsample the minority class, ensuring both classes were of equal size. Notably, the upsampling process was carried out exclusively during the training phase and did not include the test samples.

### Python packages

Python 3.7 was used as the programming language for data processing. Many Python-based packages and modules were used. For example, “NumPy” (version 1.20.3)^55^ and “pandas” (version 1.3.5)^56^ were used to read data from files, store them in data structures such as DataFrames, and manipulate data. Splitting data into iterative, mutually exclusive sets for training and testing, and constructing the predictive models was done with the help of the package “scikit-learn” (version 1.0.2)^57^. The package “imbalanced-learn” (version 0.11.0)^58^ helped in, first, up-sampling the under-sampled classes using the SMOTE technique, and in, second, constructing the processing pipeline with steps corresponding to each prediction model. Figures were generated using the package “matplotlib” (version 3.5.0)^59^. The package “statsmodels” (version 0.13.2)^60^ corrected for the multiple hypothesis tests via the false discovery rate (FDR) approach.

## Results

### Effect on cardiac performance and markers (Fig. 4)

**Fig. 4:**
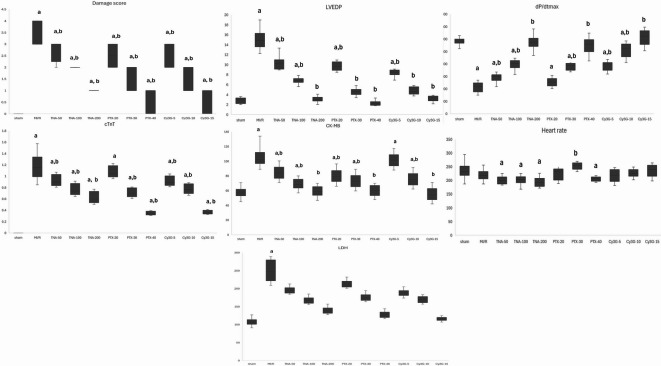
The effects of various treatments on cardiac parameters in the (MI/R) injury model. LVEDP: “Left Ventricular End-Diastolic Pressure”, dP/dtmax: “Maximum Rate of Pressure Change”, cTnT: “Cardiac Troponin T”, CK-MB: “Creatine Kinase-MB”, LDH: “Lactate Dehydrogenase”, MI/R injury: “myocardial ischemic reperfusion”, TNA: “Trans-Anethole”, PTX: “Pentoxifylline”, Cy3G: Cyanidin-3-O-glucoside, data exhibited as mean ±SD, one way ANOVA test subsequently Tukey test to compare significant with sham (a) and MI/R injury groups. (b) Dose-dependent improvements in LVEDP, dP/dtmax, and biomarkers (cTnT, CK-MB) directly informed the damage score (DS) classification (DS ≤ 2 = responsive) used for machine learning prediction. *p*-value <0.01** is considered highly significant. Dose-dependent improvements in LVEDP, dP/dtmax, and biomarkers (cTnT, CK-MB) directly informed the damage score (DS) classification (DS ≤ 2 = responsive) used for machine learning prediction.

"Compared to the sham group, MI/R injury caused significant myocardial damage (P < 0.001). Treatments with TNA, PTX, and Cy3G demonstrated dose-dependent cardioprotection, aligning with our hypothesis that drug efficacy can be quantitatively predicted via biochemical and hemodynamic features. Higher doses (TNA200, PTX-40, Cy3G-15) significantly restored cardiac function: (a) near-normalization of LVEDP and dP/dtmax (e.g., TNA200: LVEDP = 3.06 ± 0.7 mmHg vs. MI/R: 14.94 ± 1.7 mmHg; *P* < 0.001). (b) Dose-correlated reduction in injury biomarkers (cTnT: Cy3G-15 = 0.37 ± 0 ng/mL vs. MI/R: 1.19 ± 0.2 ng/mL; *P* < 0.001). Critically, successful treatment (DS ≤ 2) consistently corresponded to these biochemical improvements, validating our hypothesis that molecular/biochemical features (Fig. 4) are predictive of treatment response. This experimental outcome directly supports the machine learning framework for response prediction (Sect. 2.11–2.12)."Notably, TNA200, PTX-40, and Cy3G-15 resulted in nearly normal levels of LVEDP, dP/dtmax, and cardiac biomarkers. Reinforced that dP/dtmax and cTnT (identified as top predictors by SFS) showed the most significant dose-dependent improvements, supporting their role in model feature selection.

TNA200 and PTX-40 were particularly effective in minimizing damage scores (DS), successful treatment (DS ≤ 2) consistently corresponded to these biochemical improvements, validating our hypothesis that molecular/biochemical features (Fig. 4) are predictive of treatment response. This experimental outcome directly supports the machine learning framework for response prediction. Also, they restore cardiac function, as reflected by significant improvements in LVEDP and dP/dtmax. Similarly, Cy3G-15 demonstrated strong cardioprotection, achieving results comparable to TNA200 and PTX-40 in reducing myocardial stress and injury markers. All treatments revealed marked significant differences when compared to the MI/R injury group (P < 0.001), underscoring their therapeutic potential.

### Histopathological examination

**Fig. 5:**
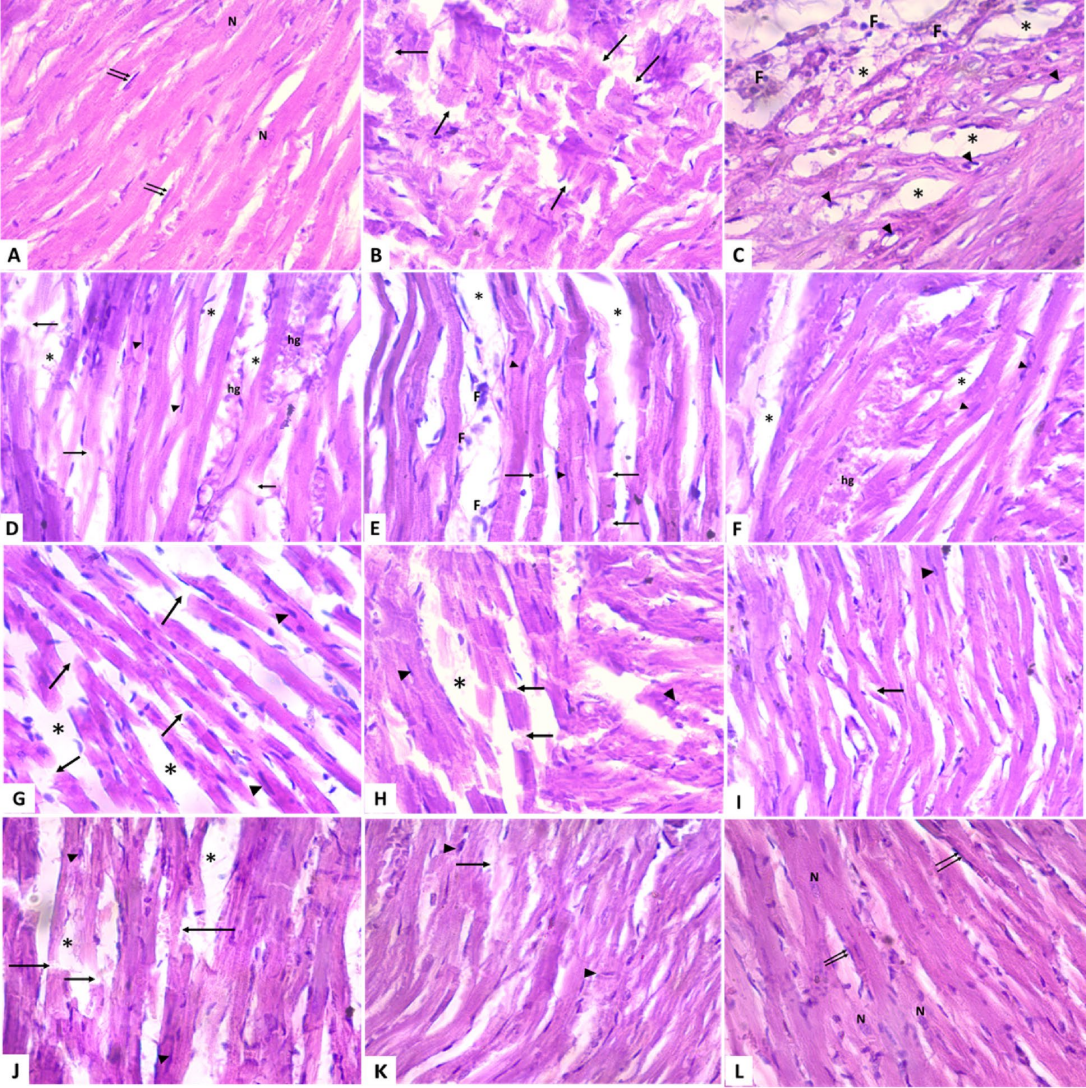
Photomicrographs showing left ventricular tissue histopathological changes of adult male rats stained by H&E staining (× 400). (**A**) The control group showing a normal histological picture of myocardium composed of branching acidophilic myocardial fibers with oval and vesicular nuclei (N), and scattered flat, darkly stained nuclei (↑↑) of connective tissue cells in between the cardiac muscle fibers. (**B** and **C**); MI/R injury group showing severe myocardial necrosis and destruction, disarray of cardiac myocytes, dark pyknotic nuclei (▲) with perinuclear edema, interstitial edema (*) and inflammatory cell (F) infiltration. (**D**–**F**); TNA 50, 100, 200 mg Treated groups respectively, showing multiple fragmented myofibrils (↑) with deep basophilic pyknotic nuclei (▲), interstitial edema (*) and areas of hemorrhage (hg). (**G**–**I**); PTX 20, 30, 40 mg treated groups respectively showed less fragmented myofibrils (↑), and interstitial edema (*). (**J**–**l**); CY3 5, 10, 15mg Treated groups respectively, showing marked improvement of the histological features of the myocardium.

On histopathological examination of left ventricular tissues of different groups using H&E stain (Fig. 5, Table 4). Normal histological appearance of myocardium composed of branching acidophilic myocardial fibers with oval and vesicular nuclei and scattered flat, darkly stained nuclei of connective tissue cells in between the cardiac muscle fibers of control group (Fig. 5a). In contrast MI/R group showed severe myocardial necrosis and destruction, disarray of cardiac myocytes, dark pyknotic nuclei with perinuclear edema, interstitial edema, and inflammatory cell infiltration (Fig. 5b,c). TNA 50, 100, 200 mg treated groups respectively, showing multiple fragmented myofibrils with deep basophilic pyknotic nuclei, interstitial edema and areas of hemorrhage (Fig. 5d–f). PTX 20, 30, 40 mg treated groups respectively showed less fragmented myofibrils, and interstitial edema (Fig. 5g–i). CY3 5, 10, 15mg treated groups respectively, showed marked enhancement of the myocardial histological features (Fig. 5j–l). Table 4: Histopathological scoring system for the heart edema , necrosis, PMNL, loss of striation and hemorrhge using morphometric analysis.

**Table 4:** Histopathological scoring system for the heart edema , necrosis, PMNL, loss of striation and hemorrhge using morphometric analysis.

Component		Necrosis				Polymorphonuclear Leucocytes				Loss of striation				Edema				Hemorrhage			
Grading range		0	1	2	3	0	1	2	3	0	1	2	3	0	1	2	3	0	1	2	3
Groups	sham	80%	10%	10%	0	90%	10%	0	0	70%	20%	10%	0	70%	30%	0	0	80%	20%	0	0
	MI/R	0	20%	20%	60%	10%	10%	30%	50%	0	10%	20%	70%	0	20%	10%	70%	10%	10%	20%	60%
	TNA 50	10%	20%	20%	50%	10%	20%	20%	50%	10%	20%	30%	40%	10%	10%	30%	50%	10%	20%	30%	40%
	TNA100	20%	10%	40%	30%	10%	20%	30%	40%	20%	40%	20%	20%	10%	30%	20%	40%	30%	40%	10%	20%
	TNA200	60%	20%	20%	0	60%	20%	10%	10%	40%	40%	10%	10%	50%	30%	10%	10%	70%	10%	10%	10%
	PTX-20	10%	20%	30%	40%	10%	20%	30%	40%	20%	40%	20%	20%	10%	20%	30%	40%	10%	20%	30%	40%
	PTX-30	20%	60%	10%	10%	40%	40%	20%	20%	30%	30%	20%	20%	40%	40%	20%	20%	30%	50%	10%	10%
	PTX-40	60%	20%	20%	0	70%	30%	0	0	50%	30%	10%	10%	60%	20%	10%	10%	80%	10%	10%	0
	Cy3G-5	30%	30%	20%	20%	30%	20%	20%	30%	40%	40%	20%	20%	60%	20%	10%	10%	30%	50%	10%	10%
	Cy3G-10	50%	30%	10%	10%	60%	20%	10%	10%	50%	30%	10%	10%	70%	10%	10%	10%	60%	20%	10%	10%
	Cy3G-15	70%	20%	10%	0	80%	20%	0	0	60%	30%	10%	0	90%	10%	0	0	80%	20%	0	0

Table showing number and frequency distribution (%) of each the degree of component examined in four different field sections in 10 rats (40 fields/ group).

**Fig. 6:**
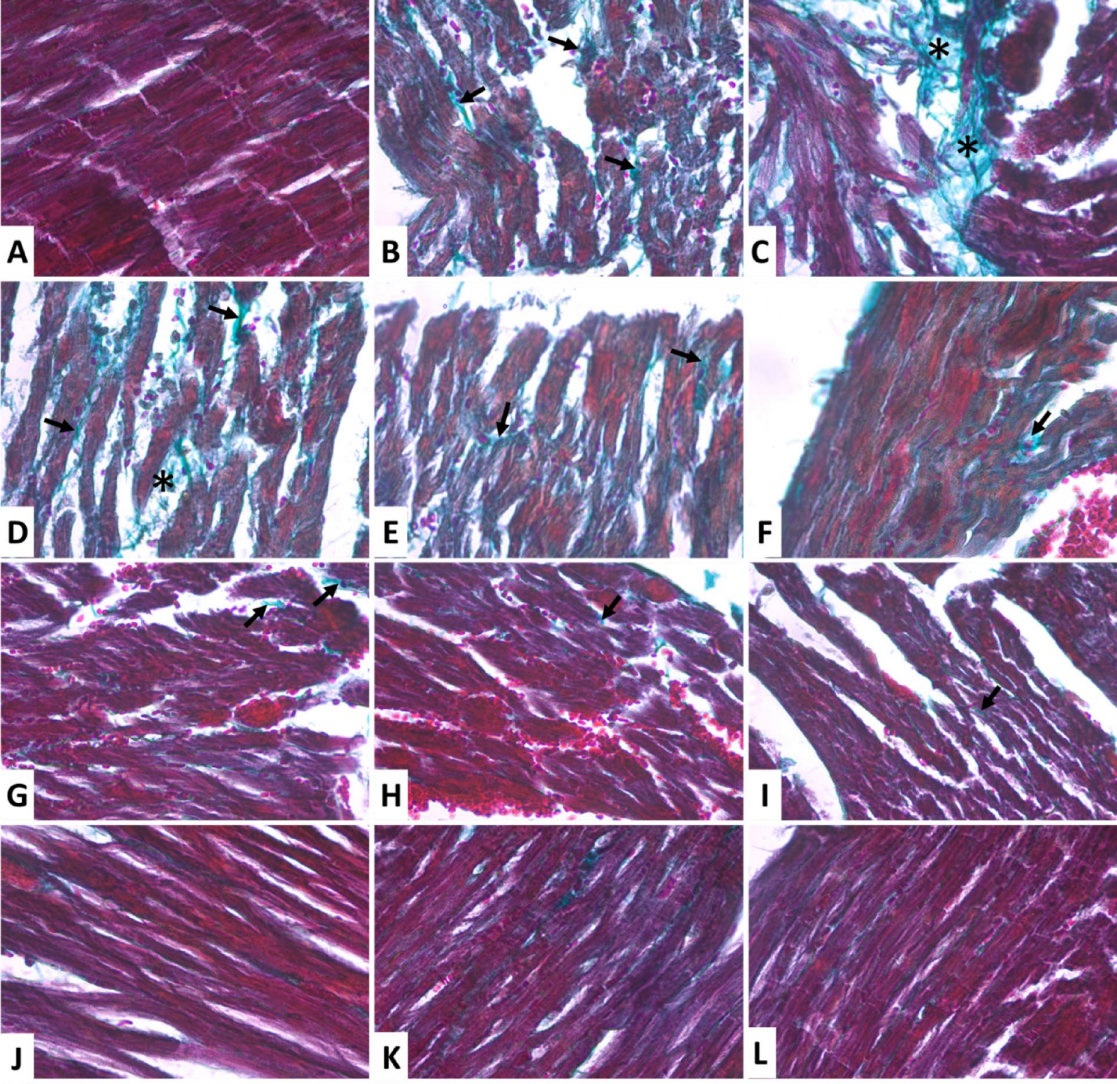
Photomicrographs of left ventricular tissue of adult male rats stained by Masson’s trichrome stain (× 400). (**A**) The control group is showing no collagen of myocardium. (**B** and **C**); MI/R injury group showing large patchy areas of collagen fibers deposition in the interstitium (*) and between distorted cardiac muscle fibers (↑). (**D**–**F**); TNA 50, 100, 200mg Treated groups respectively, showing scattered collagen fibers deposition inbetween cardiac muscle fibers (↑). (**G**–**I**); PTX 20, 30, 40mg Treated groups respectively, showing few scanty collagen fibers (↑). (**J**–**L**); CY3 5, 10, 15mg Treated groups respectively, showing no obvious collagen deposition as normal.

While using Masson’s trichrome in Fig. 6; the control group showed no collagen deposition in between cardiac muscle fibers of the myocardium (Fig. 6a). On the other hand MI/R injury group showed large patchy areas of collagen fibers deposition in the interstitium and in between distorted cardiac muscle fibers (Fig. 6b and c). Treated groups with TNA 50, 100, 200 mg respectively, showed scattered collagen fibers deposition in between cardiac muscle fibers (Fig. 6d–f);. (Fig. 6g–i); PTX 20, 30, 40mg Treated groups respectively, showing few scanty collagen fibers (↑). (Fig. 6j–l); CY3 5, 10, 15mg Treated groups respectively, showing no obvious collagen deposition as normal. Cy3G-High (30 mg/kg), TNA 200 mg/kg and PTX 40 mg/kg reduced fibrosis by > 50% (*p* < 0.001 (Fig. 5G–I) and supplementary Table S3A–B .

### Cardiac molecular markers (Fig. 7)

**Fig. 7:**
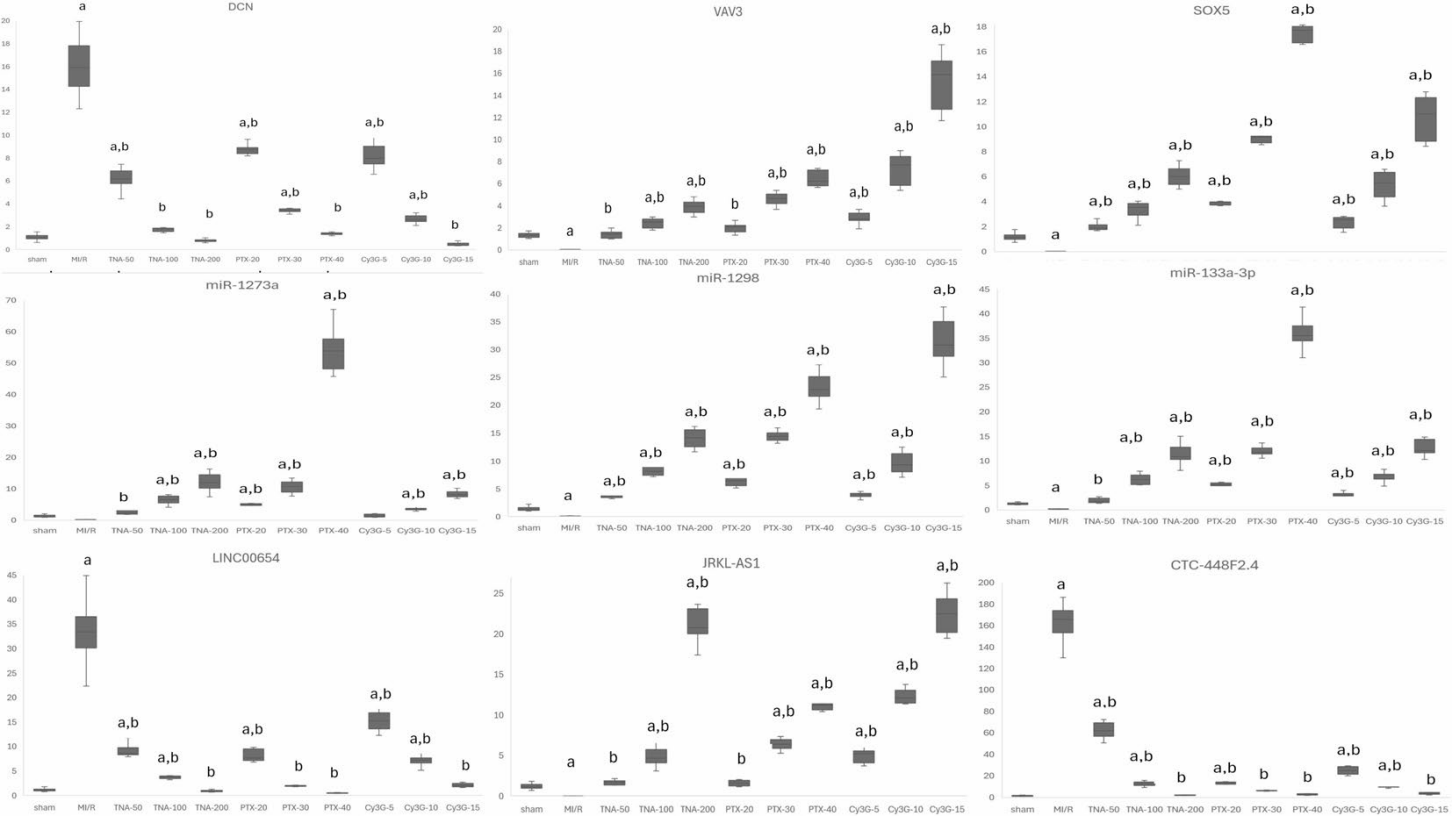
The effects of MI/R-injury and subsequent treatments on the RQs (relative expression levels) of genes, microRNAs, and lncRNAs. DCN: “Decorin”, VAV3: “Vav Guanine Nucleotide Exchange Factor 3”, SOX5: “ SRY-Box Transcription Factor 5”, LINC00654: “Long Intergenic Non-Protein Coding RNA 654”, JRKL-AS1: “ JRKL Antisense RNA 1”, MI/R injury: myocardial ischemic reperfusion, TNA: “Trans-Anethole”, PTX: “Pentoxifylline”, Cy3G: Cyanidin-3-O-glucoside, data expressed as mean±SD; one way ANOVA test, subsequently Tukey test to compare significant with sham (a) and MI/R injury groups (b). *p*-value <0.05 is considered significant.

In the MI/R injury group, RQs of DCN, LINC00654, and CTC-448F2.4 showed significant upregulation compared to the sham group, whereas RQs of VAV3, SOX5, miR-1298, miR-133a-3p, and JRKL-AS1 were markedly downregulated. RQs miR-1273a also declined but did not reach statistical significance. U6 snRNA demonstrated stable expression across all groups (Supplementary Fig. S4), validating its use as an endogenous control for miRNA normalization. These changes reflect a significant alteration in molecular expression patterns induced by MI/R injury. Treatment with TNA, PTX, and Cy3G modulated these patterns, with higher doses restoring expression levels closer to those of the control group.

Among treatments, TNA200, PTX-40, and Cy3G-15 were the most effective, achieving near-normal expression levels for several markers. Notably, PTX-40 significantly increased RQs of VAV3 and SOX5 expression, while Cy3G-15 had a strong effect on RQs of JRKL-AS1 and miR-133a-3p.

### Prediction of the treatment response using the machine learning models

Table 5 shows the features included in and excluded from the reduced models based on their importance to the classification process using the greedy SFS approach. Emphasized that higher doses universally achieved DS ≤ 2 (responsive class), directly linking experimental outcomes to the binary response variable used in machine learning Out of the 9 molecular features, only the SOX5 was found significant for the prediction process, whereas only 2 biochemical features (dP/dtmax and cTnT) were included in the final predictive models, and the remaining 4 were left out. Feature selection (SFS) prioritized dP/dtmax and cTnT—parameters showing the strongest dose–response trends in section "Effect on cardiac performance and markers (Fig. 4)"—validating their utility as key predictors. Surprisingly, when combining both the molecular and biochemical features in one model, only the two significant biochemical features were kept in, while the lonely molecular feature that previously was declared significant turned out to be of redundant information and was ruled out from the combined model.

**Table 5:** Included features and excluded features.

Model Features	Included Features	Excluded Features
	Feature	Feature
**Molecular** Included: 1 Excluded: 8 Total: 9	SOX5	DCN VAV3 miR-1273a miR-1298 miR-133a-3p LINC00654 JRKL-AS1 CTC-448F2.4
**Biochemical** Included: 2 Excluded: 4 Total: 6	dP/dtmax cTnT	LVEDP Heart Rate CK-MB LDH
**Molecular-Biochemical** Included: 2 Excluded: 13 Total: 15	dP/dtmax cTnT	DCN VAV3 SOX5 miR-1273a miR-1298 miR-133a-3p LINC00654 JRKL-AS1 CTC-448F2.4 LVEDP Heart Rate CK-MB LDH

Features kept for the reduced models are listed under the “Included Features” column, while the discarded features are listed under the “Excluded Features” column.

Table 6 through 10 present the performance metrics for both complete and reduced models developed using the logistic regression, k-nearest neighbor, neural networks, random forest, and support vector machines classifiers, respectively. These metrics include accuracy, precision, recall (sensitivity), specificity, and the Matthews Correlation Coefficient (MCC). Although the SMOTE technique was applied to balance the sample distribution between the two classification classes, the MCC remains the most reliable metric for comparing the performance of various machine learning models in binary classification tasks. A value of k = 3 was used for cross-validation in this study^61,62^. Supplementary Files 2, 3, 4, 5, and 6 detail the complete results for each iteration for each classifier.

**Table 6:** Performance assessment of using the multivariate logistic regression models.

Model	Logistic regression (LR)		
	Metric	Complete (all features)	Reduced model (SFS)
Molecular	Accuracy	0.8822 ∓ 0.0266	0.7192 ∓ 0.0194
	Precision	0.9486 ∓ 0.0409	0.8917 ∓ 0.0297
	Recall	0.8782 ∓ 0.0366	0.6721 ∓ 0.0077
	Specificity	0.8963 ∓ 0.0818	0.8222 ∓ 0.0480
	MCC	0.7474 ∓ 0.0614	0.4589 ∓ 0.049
Biochemical	Accuracy	0.8821 ∓ 0.0129	0.8765 ∓ 0.0155
	Precision	0.9491 ∓ 0.0408	0.9494 ∓ 0.0358
	Recall	0.8780 ∓ 0.0319	0.8696 ∓ 0.0489
	Specificity	0.8963 ∓ 0.0818	0.8944 ∓ 0.0749
	MCC	0.7474 ∓ 0.0385	0.7380 ∓ 0.0327
Molecular-biochemical	Accuracy	0.9155 ∓ 0.0244	0.8765 ∓ 0.0155
	Precision	0.9589 ∓ 0.0316	0.9494 ∓ 0.0358
	Recall	0.9176 ∓ 0.0502	0.8696 ∓ 0.0489
	Specificity	0.9130 ∓ 0.0636	0.8944 ∓ 0.0749
	MCC	0.8177 ∓ 0.0453	0.7380 ∓ 0.0327

Results are reported in the (average accuracy ∓ variance) format for all the 3-folds used to cross-validate the models. Note that recall is also the sensitivity metric. MCC, Matthews correlation coefficient.

For the complete models (Tables 7 and 8) and, generally speaking, for the different classifiers, the MCC values are better for the models using the molecular features alone over those from their corresponding models using the biochemical features alone. But when using the reduced models, the MCC values for the biochemical features-based models are far better than those from the molecular features-based models. This can be attributed to the remarkable number of the molecular features that are excluded by the SFS method, where only one feature (SOX5) is included in the final reduced model out of nine features. But for the reduced biochemical modes, two features (dP/dtmax and cTnT) are kept out of six features. The reduced combined (molecular and biochemical) models have similar MCC values to those from the reduced biochemical models, as they exclude any molecular features and keep only the two biochemical features dP/dtmax and cTnT. But the complete combined model showed mixing patterns, where it has better MCC values for the LR, kNN, and RF classifiers over the models using either the molecular and biochemical features, whereas the MCC values are worse for the combined models using the NN and SVM classifiers.

**Table 7:** Performance assessment of using the k-nearest neighbors (kNN) models.

Model	k-nearest neighbor (kNN)		
	Metric	Complete (all features)	Reduced model (SFS)
Molecular	Accuracy	0.8877 ∓ 0.0076	0.8428 ∓ 0.0198
	Precision	0.9430 ∓ 0.0419	0.9284 ∓ 0.0320
	Recall	0.8951 ∓ 0.0561	0.8369 ∓ 0.0260
	Specificity	0.8796 ∓ 0.0917	0.8593 ∓ 0.0618
	MCC	0.7601 ∓ 0.0079	0.6651 ∓ 0.0487
Biochemical	Accuracy	0.8710 ∓ 0.0275	0.9156 ∓ 0.0242
	Precision	0.9404 ∓ 0.0430	0.9670 ∓ 0.0234
	Recall	0.8700 ∓ 0.0384	0.9101 ∓ 0.0572
	Specificity	0.8778 ∓ 0.0875	0.9296 ∓ 0.0500
	MCC	0.7222 ∓ 0.0682	0.8209 ∓ 0.0342
Molecular-biochemical	Accuracy	0.9046 ∓ 0.0150	0.9156 ∓ 0.0242
	Precision	0.9572 ∓ 0.0228	0.9670 ∓ 0.0234
	Recall	0.9028 ∓ 0.0374	0.9101 ∓ 0.0572
	Specificity	0.9130 ∓ 0.0445	0.9296 ∓ 0.0500
	MCC	0.7930 ∓ 0.0266	0.8209 ∓ 0.0342

Results are reported in the (average accuracy ∓ variance) format for all the 3-folds used to cross-validate the models. Note that recall is also the sensitivity metric. MCC, Matthews correlation coefficient.

**Table 8:** Performance assessment of using the neural networks (NN) models.

Model	Neural networks (NN)		
	Metric	Complete (all features)	Reduced model (SFS)
Molecular	Accuracy	0.8766 ∓ 0.0200	0.7702 ∓ 0.0827
	Precision	0.9176 ∓ 0.0202	0.9273 ∓ 0.0280
	Recall	0.9025 ∓ 0.0503	0.7250 ∓ 0.1230
	Specificity	0.8222 ∓ 0.0480	0.8759 ∓ 0.0489
	MCC	0.7228 ∓ 0.0311	0.5699 ∓ 0.1327
Biochemical	Accuracy	0.8541 ∓ 0.0274	0.9103 ∓ 0.0203
	Precision	0.9325 ∓ 0.0478	0.9570 ∓ 0.0118
	Recall	0.8538 ∓ 0.0504	0.9107 ∓ 0.0283
	Specificity	0.8593 ∓ 0.0999	0.9111 ∓ 0.0240
	MCC	0.6887 ∓ 0.0721	0.8009 ∓ 0.0455
Molecular-biochemical	Accuracy	0.8653 ∓ 0.0473	0.9103 ∓ 0.0203
	Precision	0.9086 ∓ 0.0218	0.9570 ∓ 0.0118
	Recall	0.8952 ∓ 0.0588	0.9107 ∓ 0.0283
	Specificity	0.8019 ∓ 0.0567	0.9111 ∓ 0.0240
	MCC	0.6939 ∓ 0.1095	0.8009 ∓ 0.0455

Results are reported in the (average accuracy ∓ variance) format for all the 3-folds used to cross-validate the models. Note that recall is also the sensitivity metric. MCC, Matthews correlation coefficient.

A general note is that the RF classifier did not provide remarkable differential prediction power regardless of the included type of features and whether the SFS features reduction method was applied. Another general note is that the universal best MCC value for each classifier comes from the reduced models, whether they use the biochemical features or both the molecular and biochemical features, due to the above observation regarding the intense exclusion applied to the molecular features (Tables 9 and 10).

**Table 9:** Performance assessment of using the random forests (RF) models.

Model	Random forest (RF)		
	Metric	Complete (all features)	Reduced model (SFS)
Molecular	Accuracy	0.8765 ∓ 0.0155	0.8709 ∓ 0.0148
	Precision	0.9402 ∓ 0.0288	0.9464 ∓ 0.0209
	Recall	0.8786 ∓ 0.0503	0.8709 ∓ 0.0267
	Specificity	0.8778 ∓ 0.0595	0.8944 ∓ 0.0388
	MCC	0.7348 ∓ 0.0261	0.7244 ∓ 0. 0314
Biochemical	Accuracy	0.8877 ∓ 0.0210	0.8990 ∓ 0.0130
	Precision	0.9183 ∓ 0.0300	0.9333 ∓ 0.0119
	Recall	0.9182 ∓ 0.0096	0.9182 ∓ 0.0096
	Specificity	0.8241 ∓ 0.0571	0.8574 ∓ 0.0233
	MCC	0.7410 ∓ 0.0467	0.7680 ∓ 0.0315
Molecular-biochemical	Accuracy	0.8990 ∓ 0.0130	0.8990 ∓ 0.0130
	Precision	0.9504 ∓ 0.0398	0.9333 ∓ 0.0119
	Recall	0.9026 ∓ 0.0327	0.9182 ∓ 0.0096
	Specificity	0.8963 ∓ 0.0818	0.8574 ∓ 0.0233
	MCC	0.7794 ∓ 0.0371	0.7680 ∓ 0.0315

Results are reported in the (average accuracy ∓ variance) format for all the 3-folds used to cross-validate the models. Note that recall is also the sensitivity metric. MCC, Matthews correlation coefficient.

**Table 10:** Performance assessment of using the support vector machines (SVM) models.

Model	Support vector machines (SVM)		
	Metric	Complete (all features)	Reduced model (SFS)
Molecular	Accuracy	0.9047 ∓ 0.0340	0.6965 ∓ 0.0374
	Precision	0.9907 ∓ 0.0131	0.8286 ∓ 0.0563
	Recall	0.8700 ∓ 0.0384	0.7046 ∓ 0.0092
	Specificity	0.9815 ∓ 0.0262	0.6833 ∓ 0.0970
	MCC	0.8079 ∓ 0.0690	0.3636 ∓ 0.0926
Biochemical	Accuracy	0.8540 ∓ 0.0202	0.8932 ∓ 0.0084
	Precision	0.9339 ∓ 0.0588	0.9508 ∓ 0.0398
	Recall	0.8540 ∓ 0.0495	0.8942 ∓ 0.0493
	Specificity	0.8630 ∓ 0.1238	0.8963 ∓ 0.0818
	MCC	0.6924 ∓ 0.0568	0.7713 ∓ 0.0012
Molecular-biochemical	Accuracy	0.8708 ∓ 0.0210	0.8932 ∓ 0.0084
	Precision	0.9431 ∓ 0.0618	0.9508 ∓ 0.0398
	Recall	0.8700 ∓ 0.0384	0.8942 ∓ 0.0493
	Specificity	0.8815 ∓ 0.1303	0.8963 ∓ 0.0818
	MCC	0.7263 ∓ 0.0608	0.7713 ∓ 0.0012

Results are reported in the (average accuracy ∓ variance) format for all the 3-folds used to cross-validate the models. Note that recall is also the sensitivity metric. MCC, Matthews correlation coefficient.

Notably, the combined complete molecular-biochemical model for kNN yielded solid performance. The reduced model maintained or slightly improved the already strong performance of the complete version, and performing best overall achieving 91.56% accuracy, 96.70% precision, 91% Recall , Specificity 92% and an MCC of 0.82.

**Fig. 8:**
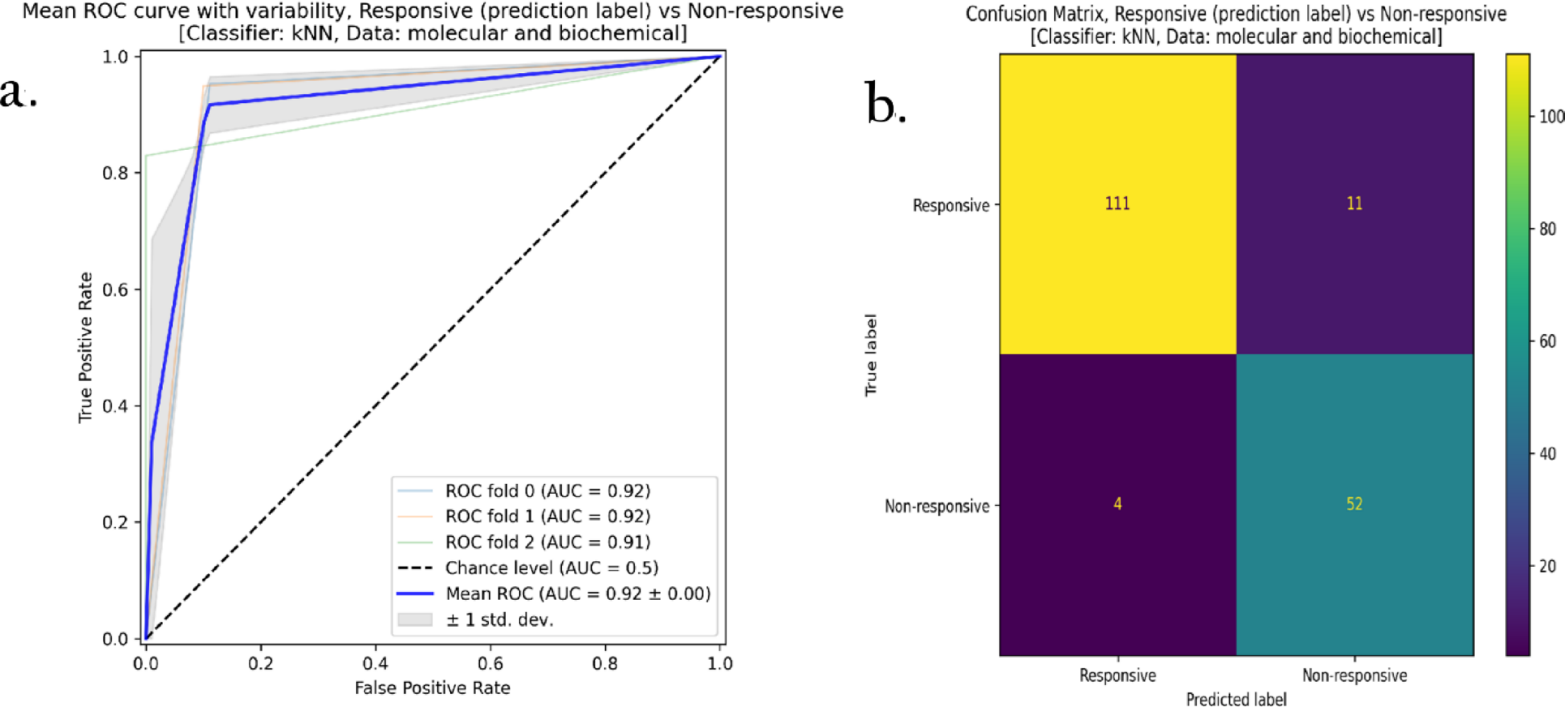
(**a**) The receiver operating characteristics (ROC) curve, and (**b**) the confusion matrix for the reduced model with the kNN classifier. Data used are both the molecular and biochemical features.

**Fig. 9:**
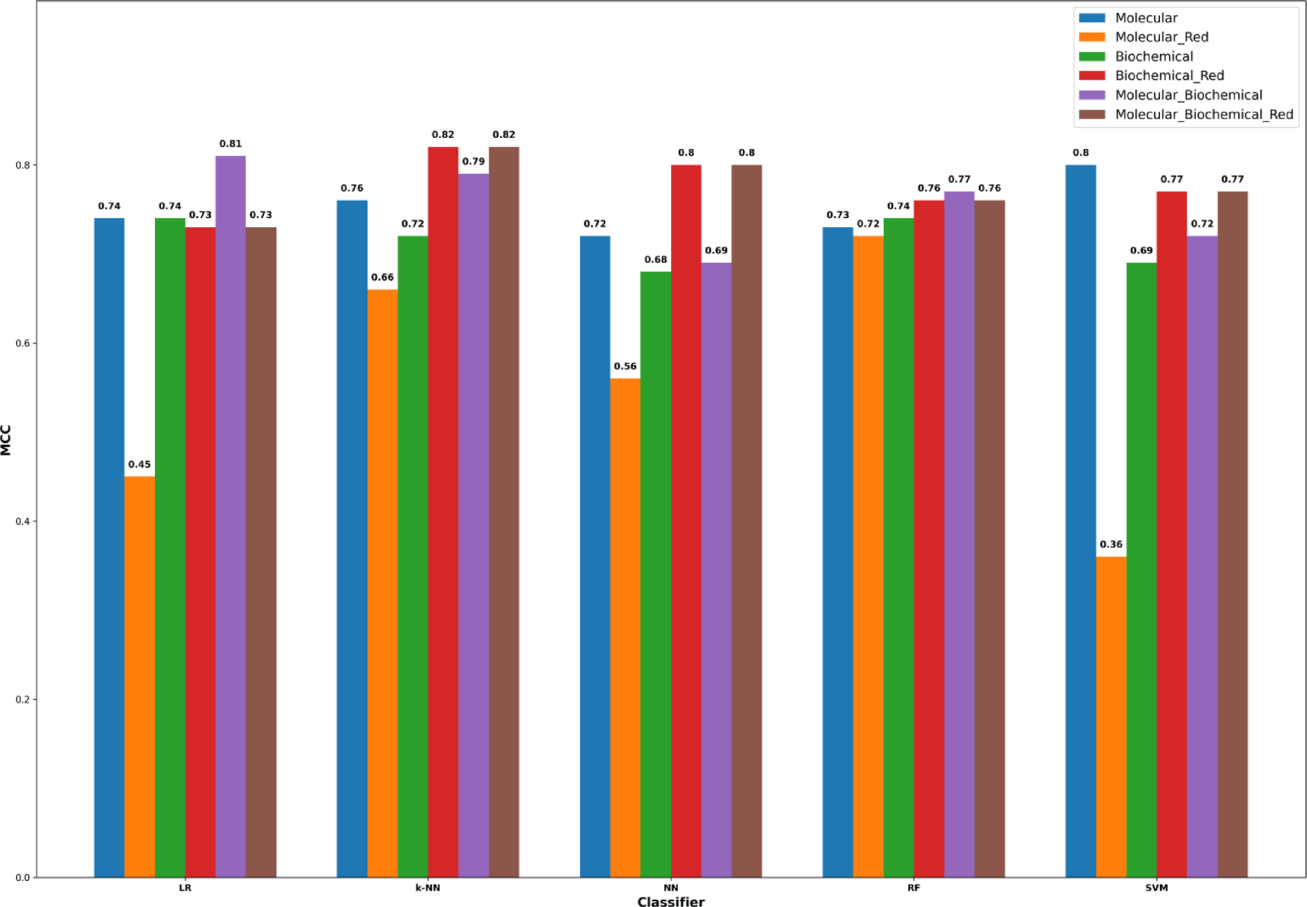
The Matthews correlation coefficient (MCC) for the linear regression (LR), k-nearest neighbor (k-NN), neural network (NN), random forest (RF), and support vector machines (SVM) classifiers for the complete model (all the features) and the reduced (Red) model obtained by applying the SFS approach.

An example of the receiver operating characteristics (ROC) curve for the reduced model built using the kNN classifier and employing the molecular and biochemical features is shown in Fig. 8.a, whereas the confusion matrix for the same machine learning model is shown in Fig. 8.b. This model achieved an average area under the ROC curve (AUC) of 0.90 over the cross-validation iterations (Fig. 8.a). In addition, this model was able to successfully predict 111 responsive samples (positives) out of the 122-ground-truth responsive samples (true positives) and 52 non-responsive samples (negatives) out of the 56-ground truth non-responsive samples (true negatives), as depicted in Fig. 8b.The Supplementary Information contains the ROC curves besides the confusion matrices (Figure S5 and S6) for all the constructed models. Additionally, a comprehensive summary of the Matthews Correlation Coefficient (MCC) for all classifiers is presented in Fig. 9.

**Fig. 10:**
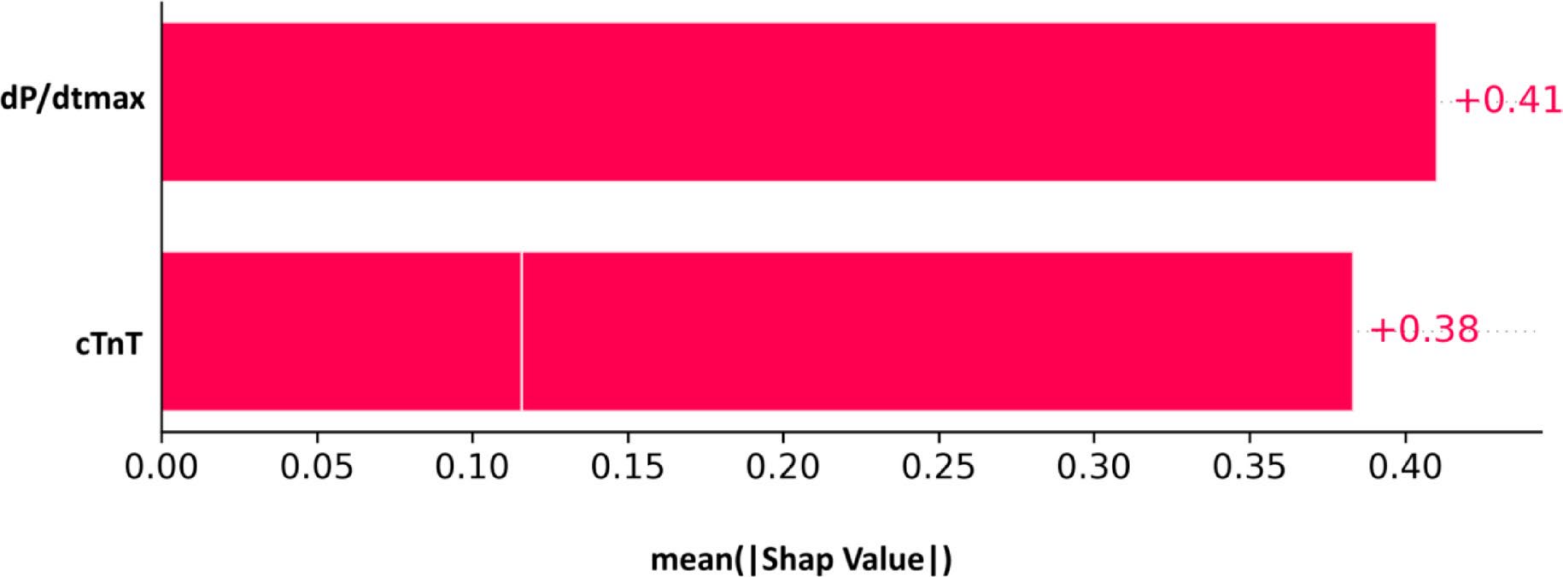
Shap values for the reduced model of the biochemical features using the kNN classifier.

Figure 10 shows the Shap values for the best prediction model that was obtained using the kNN classifier while utilizing the molecular features and turning on the feature reduction step through the greedy SFS approach. We can see that the two features dP/dtmax and cTnT have almost the same contribution to the model predictability, which aligns with the purpose of the feature reduction step.

## Discussion

Myocardial reperfusion is a widely used intervention for AMI injury; however, it can exacerbate the condition by inducing MI/R injury. MI/R injury involves inflammation, oxidative stress, and apoptosis, which collectively worsen cardiac damage and limit the therapeutic benefits of reperfusion therapy^2,3,5^. Thus, there remains a need for effective cardioprotective approaches that can alleviate or reverse I/R-induced biochemical alterations in IHD^63,64^.

This study aimed to develop an I/R rat model and evaluate the therapeutic potential of TNA, PTX, and Cy3G in mitigating MI/R-induced cardiac damage through modulation of genetic and epigenetic pathways.

Our findings confirmed significant deterioration in cardiac function, biomarkers, and histopathology in the MI/R group. This was evidenced by increased LVEDP, reduced dP/dtmax, elevated biomarkers (cTnT, CK-MB, LDH), extensive myocardial destruction, interstitial edema, and inflammatory infiltration. Our findings are consistent with prior studies, such as Zhang et al. (2019), which reported compromised myocardial integrity^65^, and Liu et al. (2014), which identified cTnT, CK-MB, and LDH as key biomarkers for myocardial necrosis and oxidative stress^66^.

TNA demonstrated cardioprotective effects by decreasing LVEDP and increasing dP/dtmax, along with dose-dependent reductions in myocardial damage markers. Similarly, PTX improved cardiac function, with its most significant effects observed at 40 mg/kg^67^, while Cy3G improved cardiac markers and heart function, with 15 mg/kg showing the best results. Treatments with TNA, PTX, and Cy3G demonstrated dose-dependent benefits, with the highest doses (TNA200, PTX40, and Cy3G15) restoring parameters close to normal levels. These improvements are consistent with previous reports emphasizing the importance of anti-inflammatory and antioxidant therapies in preserving cardiac function post-MI/R^68^.

Histopathological analyses revealed that TNA, PTX, and Cy3G alleviated pathological changes, with Cy3G15 showing the most pronounced recovery of myocardial architecture. This finding supports the established role of Cy3G in reducing fibrosis and promoting cellular repair. The histopathological analysis demonstrates severe myocardial injury in the MI/R group, with 60–70% of samples exhibiting Grade 3 necrosis, inflammation, loss of striation, edema, and hemorrhage. Therapeutic interventions revealed dose-dependent protection. TNA 200 mg/kg reduced severe necrosis from 60 to 0%, inflammation from 50 to 10%, and loss of striation from 70 to 10%; PTX 40 mg/kg eliminated severe necrosis (60% → 0%) and inflammation (70% → 0%), while normalizing hemorrhage (60% → 0%); Cy3G-15 mg/kg achieved near-complete recovery, with 80–90% of samples showing Grade 0 (normal) for edema and hemorrhage, outperforming other groups. Intermediate doses (e.g., TNA 50 mg/kg, PTX 20 mg/kg) offered limited efficacy, underscoring the necessity for optimal dosing. Overall, Cy3G-15 mg/kg emerged as the most effective treatment, while TNA 200 mg/kg and PTX 40 mg/kg provided viable alternatives with specific strengths in necrosis reduction and inflammation control, respectively.

TNA has been highlighted in related studies for its anti-inflammatory and epigenetic regulation in I/R models^69^. It significantly reduced TNF-α levels, improved cardiac function, reversed ECG changes and downregulated inflammatory lncRNAs and miRNAs, providing a complementary approach to PTX and Cy3G^2,70^. TNA exhibits anti-inflammatory function by inhibiting the TGF-β pathway, diminishing hepatic steatosis, fibrosis, and proinflammatory cytokines^71^. TNA also exhibits antihypertensive action by reducing arterial remodeling and improving vascular relaxation^72,73^. Furthermore, TNA has an anti hyperlipidemic effect through inhibition of the mTOR/PPARγ pathway crucial for lipid metabolism^74^.

Also, TNA ameliorates kidney injury and decreases TGF-β and angiotensin II receptor expressions in streptozotocin-induced diabetic rats^75^. PTX exhibits potent antioxidant activity, upregulating Nrf2 and reducing oxidative stress^76^. PTX, known for its anti-inflammatory effects, reduced cytokine levels (TNF-α, IL-1β, and CRP) and improved outcomes in cardiac I/R models by decreasing oxidative stress and apoptosis^77,78^. Additionally, PTX, as part of multi-drug interventions, demonstrated improved outcomes in cardiac I/R models by reducing oxidative stress, inflammation, and apoptosis^27,79^.

Cy3G, an important anthocyanin in plant food, with its potent antioxidant properties, upregulated Nrf2, reduced inflammatory markers such as IL-6, TNF-α & IL-1, and restored myocardial architecture^80^. Cy3G diminished inflammatory and fibrotic processes, thereby reducing cardiac hypertrophy both in vitro and in vivo via inhibition of the CTRP3/AMPK pathway ^81^. Our data revealed the dose-dependent efficacy of Cy3G suggests saturation of its anti-inflammatory pathway at 15 mg/kg".Also, Cy3G uniquely normalized 90% of edema cases at 15 mg/kg . Cy3G-treated cardiac tissues showed reduced fibrosis, edema, and inflammation, indicating its ability to restore myocardial architecture consistent with findings by Liu et al. (2014). Cy3G protected against MI/R injury could be related to decreased cytosolic cytochrome c^82^. Cy3G enhanced angiogenesis and decreased blood pressure in rats^83^. Cy3G improved hemodynamics parameters, arterial wall structure, and endothelial cell functions and decreased blood lipid levels in high-fat-diet rabbits^84^.

The dose-dependent effectiveness of TNA200, PTX40, and Cy3G15 is consistent with known optimal dosing strategies that maximize cardioprotective effects. Cardioprotective agents like zoniporide, a sodium-hydrogen exchanger inhibitor, show a clear dose-dependent reduction in infarct size during myocardial I/R injury^85^. Compounds targeting mitochondrial permeability transition pore (PTP), such as TRO40303, exhibit dose-related efficacy in reducing apoptosis and improving mitochondrial function, providing insight into dose optimization strategies^86^. This underscores the importance of precision dosing to maximize therapeutic benefits while minimizing adverse effects.

On the molecular level, numerous bioinformatics and experimental studies have demonstrated the roles of ncRNA interactions, specifically between miRNAs and lncRNAs, as well as their interactions with messenger RNAs (mRNAs). miRNAs can bind complementarily to their target mRNAs, leading to mRNA degradation or translational suppression. LncRNAs can act as sponges for miRNAs, preventing them from targeting mRNAs^87^.

The molecular mechanisms underlying the selected mRNAs (DCN, VAV3 & SOX5) were explored in this study. Decorin (DCN), an extracellular matrix proteoglycan, is upregulated in MI/R and contributes to fibrosis and inflammation via TGF-β signalling^88–90^. VAV3, a Rho GTPase guanine nucleotide exchange factor, is involved in promoting cardiomyocyte survival and is downregulated in MI/R injury^46^. It also enhances cell proliferation in endometrial cancer^91^, gastric cancer^92^ and pancreatic cancer^93^. SOX5 is a member of the SRY-box family transcription factors^94^. SOX5, previously reported to reverse nerve injury by cerebral ischemia via activation of the PI3K/AKT pathway^95^. SOX5 was involved in myocardial repair in our work. Cy3G downregulated DCN expression, reducing fibrosis and improving cardiac outcomes (Khedr et al., 2022). VAV3, and SOX5 were upregulated by TNA and PTX, highlighting their roles in mitigating apoptosis and inflammation^67,96^.

miR-1298 is engaged in signaling of the PP2A/AMPK/GSK3β pathway in the pathogenesis of MI/R injury^97^. miRNA 1273 has been reported to be associated with endocapillary glomerular inflammation and was downregulated in lupus nephritis patients^98^. miR-1298 and miR-1273a, suppressed in MI/R, were restored by TNA and Cy3G^67,68^, reducing inflammation and fibrosis. Several studies explored the role of miR-133a-3p in MI/R injury via inhibition of apoptosis, fibrosis and induction of cardiac reprogramming^99–104^, miR-133a-3p was significantly upregulated by all three treatments as in previous publications^96^.

LINC00654, which is on the reverse strand of chromosome 20, could has a potential diagnostic marker in colorectal cancer^105,106^, is associated with a higher risk of large B cell lymphoma^107^, and is associated with poor overall survival in breast cancer^108^. Additionally, lncRNAs LINC00654 and CTC-448F2.4, upregulated in MI/R, were downregulated by Cy3G and PTX, while JRKL-AS1, upregulated by TNA, played a key role in cardioprotection as previously reported^67,68,96^.

This study also highlights the interplay of genetic and epigenetic regulators in MI/R injury. The downregulation of LINC00654, and CTC-448F2.4 reduced inflammatory responses, while the upregulation of miR-1298, miR-133a-3p, miR-1273a and JRKL-AS1 promoted anti-inflammatory signalling. Restoration of DCN, SOX5 and VAV3 levels by all treatments mitigated apoptotic pathways, preserving cardiomyocyte integrity.

Similar to our findings, Khedr et al. (2022) reported that DCN was upregulated; however, CTC-448F2.4 and miR1273a were downregulated in MI/R, contributing to fibrosis and inflammation, while Cy3G restored molecular markers, improving cardiac outcomes. TNA upregulated VAV3 and miR-1298 and increased JRKL-AS1 expression, modulating gene networks crucial for cardiac protection (Matboli et al., 2020). PTX upregulated SOX5, and miR-133a-3p and inhibited LINC00654, reducing fibrosis and inflammation^96^.

The modulation of ncRNAs underscores their potential as biomarkers and therapeutic targets in MI/R injury. These findings advocate for integrating genetic and epigenetic approaches to advance therapeutic strategies for ischemic heart disease.

Machine learning has increasingly emerged as a significant research tool in medicine^109,110^. By imitating human learning processes, ML autonomously extracts information from extensive clinical datasets^111,112^, thereby overcoming the constraints posed by human factors and variability in traditional analyses. ML has found successful applications across various areas in the cardiovascular domain, such as disease prediction^113–115^ and diagnostic classification^116^. Also, machine learning algorithms are increasingly utilized for tailoring personalized predictions of drug responses^117^. These algorithms enable the integration of data from various sources in a statistically robust way, supporting the identification of predictive biomarkers^118,119^. Thus, in this study, we utilized bioinformatics and machine learning techniques to identify robust features from a panel of 9 molecular markers, 6 biochemical markers, and their combinations to predict responses to three drugs (TNA, PTX, and Cy3G) at varying dosages in an I/R rat model. ML models trained on study data identified dose-specific thresholds for maximal efficacy.

To refine these models, we applied the greedy forward sequential feature selection (SFS) method with random forests as the estimator, combined with threefold cross-validation, aiming to determine the smallest feature set that could effectively represent each model. This process identified SOX5 as the sole molecular feature in the reduced molecular model, while the reduced biochemical model retained dP/dtmax and cTnT as key features.

Our study showed that the complete models, broadly speaking, across the various classifiers, the MCC values were generally higher for models utilizing molecular features alone compared to those relying solely on biochemical features. However, when examining the reduced models, the MCC values for the biochemical feature-based models significantly outperformed those of the molecular feature-based models. While the combined complete model exhibited a mixed performance pattern: it achieved higher MCC values for the LR, kNN, and RF classifiers compared to models using only molecular or biochemical features, yet lower MCC values for the NN and SVM classifiers. A noteworthy observation is that the RF classifier did not demonstrate significant differential predictive performance, irrespective of the feature set or the application of the SFS feature reduction method. Finally, the highest MCC value for each classifier consistently emerged from the reduced models, whether they incorporated biochemical features alone or a combination of molecular and biochemical features.

The reduced model built with the kNN classifier, utilizing both molecular and biochemical features, demonstrated robust performance with an averaged AUC of 0.90 across cross-validation iterations. This model accurately predicted 111 of the 122 true responsive samples (true positives) and correctly classified 52 of the 56 true non-responsive samples (true negatives).

Previous studies have demonstrated the potential of machine learning in exploring cardiovascular diseases and identifying therapeutic biomarkers and drug responses. Venkatesan et al. (2010) developed a scalable predictive modeling framework that integrates mutation profiles, copy number alterations and genome-scale mRNA expression from the Cancer Cell Line Encyclopedia (CCLE). Their models achieved over 70% sensitivity and specificity for various compounds, validating known genetic predictors and identifying novel features^120^. Similarly, Wang et al. (2016) introduced the Predict Drug Response in Cancer Cells (PDRCC) framework; through integrating heterogeneous pharmacogenomics data, including genomic alterations and chemical/therapeutic properties of drugs, the study achieved enhanced prediction coverage using a kernel-based SVM model, PDRCC correlated cancer cell genomic properties with drug responses, enabling predictions for both new drugs and cell lines, thus potentially reducing screening costs^121^. Furthermore, Zhang et al. (2015) proposed a novel dual-layer integrated network model that combines cell line similarity and drug similarity networks. This approach leveraged weighted models to improve drug response predictions, demonstrating the utility of integrating cell line and drug-specific features^122^. Costello et al. (2014) focused on breast cancer cell lines, utilizing proteomic, epigenomic and genomic and data to predict optimal treatment strategies. Their work highlighted the importance of multi-omic data integration in precision medicine applications^123^. Additionally, Huang C. et al. (2018) and Huang H.H. et al. (2018) applied a network-constrained logistic regression method to integrate gene expression data with biological network knowledge. Their models successfully predicted sensitivity to targeted therapies like erlotinib and sorafenib using baseline tumor gene expression data and IC50 values, providing a robust framework for in vivo drug response prediction^124^.

Samadishadlou et al. (2023) emphasized the promising role of microRNAs (miRNAs) as diagnostic biomarkers for cardiovascular conditions, particularly myocardial infarction (MI), stable coronary artery disease (CAD), and healthy individuals. The study utilized machine learning and bioinformatics techniques to analyze three gene expression datasets derived from PBMC samples available in the GEO database. Two key miRNA sets were identified: a differentially expressed set and an optimal set selected based on AUC-ROC. A two-layer classification architecture was implemented, first distinguishing healthy from unhealthy samples, then differentiating MI from stable CAD. The SVM model trained on the AUC-ROC-selected biomarkers achieved excellent performance, with an AUC-ROC of 0.96 and an accuracy of 0.94^125^. Li et al. (2023) performed a weighted gene co-expression network analysis integrated with differentially expressed gene analysis to identify potential candidate genes associated with the pathogenesis of AMI. Using machine-learning algorithms such as SVM-REF, LASSO and RF, 7 optimal feature genes were selected. Their predictive performance was evaluated using receiver operating characteristic (ROC) curves. The expression levels of these seven genes were validated in 80 AMI samples and 71 normal samples. Results showed significant upregulation of these genes in AMI samples, highlighting their potential roles in AMI progression. ROC curve analysis further confirmed their high diagnostic value, with AUC values ranging from 0.827 to 0.849 for genes like THBD, NFIL3, MCEMP1, IRAK3, IL1R2, GABARAPL1 and ACSL1 demonstrating their effectiveness in estimating AMI progression^126^.Of note, our approach offers key advances unlike most previous studies that rely on a single feature layer, our model combines both biochemical markers and molecular (gene expression) data to capture a broader biological profile relevant to drug response. This multimodal integration improves prediction accuracy and biological interpretability. Moreover, the dataset includes 176 well-characterized mouse samples across control, disease, and multiple treatment groups — a relatively robust sample size for an in vivo experimental model. These contributions together make our approach a step toward more comprehensive and biologically grounded prediction models.

Study limitations include the following; (a) While rodent models are well-established for initial MI/R injury studies, findings need further translational validation of biomarkers in human samples and collaborate with clinical partners for translational trials. (b) Sample Size Constraints because of resource constraints and ethical guidelines for animal studies necessitated this design. Nevertheless, our ML models achieved robust accuracy despite the sample size. (c) Our primary aim was to identify predictive features for drug response using ML. However, we acknowledge that mechanistic insights (e.g., SOX5’s role in PI3K/AKT pathway) are critical. Also, applying Spatial multi-omics in future work is essential to map biomarker distribution within ischemic zones, resolve cellular heterogeneity, and validate ML predictions at subcellular resolution. (d) long-term impacts on functional recovery, and safety remain unknown. (e) Expanded drug screening with broader phytochemical libraries is still required. More follow-up studies measuring serum TNFα and myocardial NF-κB in the same model is strongly recommended.

## Conclusion

In conclusion, this study highlights the dose-dependent cardioprotective effects of TNA, PTX, and Cy3G, primarily targeting inflammation and epigenetic regulators. Using advanced machine learning models combined with sequential forward selection (SFS), we identified key biomarkers, including SOX5, dP/dtmax, and cTnT, with strong predictive potential for treatment response. These models demonstrated high accuracy and robust performance, paving the way for more precise and individualized therapeutic strategies for myocardial ischemia.

## Supplementary Information

Below is the link to the electronic supplementary material.

Supplementary Material 1.

Supplementary Material 2.

Supplementary Material 3.

Supplementary Material 4.

Supplementary Material 5.

Supplementary Material 6.

Supplementary Material 7.

Supplementary Material 8.

## Data Availability

The datasets supporting the conclusions of this article are included within the article and its additional files.

## Abbreviations

Akt: Protein kinase B
AMI: Acute myocardial infarction
ANOVA: Analysis of variance
BAX: Bcl-2-associated X
CBP: CAMP response element-binding protein
CK-MB: Creatine kinase-MB
CTC-448F2.4: A specific long non-coding RNA
cTnT: Cardiac troponin T
CV: Cross-validation
Cy3G: Cyanidin-3-O-Glucoside
DAMPs: Damage-associated molecular patterns
DCN: Decorin
DEGs: Differentially expressed genes
dP/dtmax: Maximum rate of pressure change
ERK: Extracellular signal-regulated kinase
GEO: Gene expression omnibus
IHD: Ischemic heart disease
IL-1β: Interleukin-1 beta
IL-6: Interleukin-6
JRKL-AS1: JRKL antisense RNA 1
kNN: K-nearest neighbors
LDH: Lactate dehydrogenase
LINC00654: Long intergenic non-protein coding RNA 654
lncRNA: Long non-coding RNA
LR: Logistic regression
LVEDP: Left ventricular end-diastolic pressure
MAPK: Mitogen-activated protein kinase
MCC: Matthews correlation coefficient
MDA: Malondialdehyde
MI/R: Myocardial ischemia–reperfusion
miRNA: MicroRNA
mTOR: Mechanistic target of rapamycin
NFκB: Nuclear factor kappa B
NIH: National Institutes of Health
NLRP3: NOD-, LRR-, and pyrin domain-containing protein 3
NN: Neural networks
PI3K: Phosphoinositide 3-kinase
PTX: Pentoxifylline
qPCR: Quantitative polymerase chain reaction
RF: Random forests
ROC: Receiver operating characteristics
ROS: Reactive oxygen species
SD: Standard deviation
SFS: Greedy forward sequential feature selection
SMOTE: Synthetic minority oversampling technique
SOD: Superoxide dismutase
SOX5: SRY-box transcription factor 5
SVM: Support vector machines
TGF-β: Transforming growth factor beta
TNA: Trans-Anethole
TNF-α: Tumor necrosis factor alpha
VAV3: Vav guanine nucleotide exchange factor 3
