# Supplementary material for: Machine learning-based prediction of drug response in ischemia reperfusion animal model
Source: Sci Rep. 2025 Dec 2;15:43012. doi: 10.1038/s41598-025-18620-8 (PMC12672590; doi:10.1038/s41598-025-18620-8)
Supplement: Supplementary file 7 — Supplementary Material 7. [file 41598_2025_18620_MOESM7_ESM.docx]

**Supplementary Table 1:** The chemicals and drugs used in the study.

**Supplementary Table 2:** Histopathological grading of the cardiac muscle.

**Supplementary Table 3A:** Fibrosis Metrics Across Experimental Conditions.

**Supplementary Table 3A:** Comparative Efficacy of the drugs on fibrosis

**Supplementary Table 4:** Details of the GSE61592 and GSE14843 datasets were retrieved from the GEO database.

**Supplementary Table** 5A: showing binding scores, seed match between miRNAs with the selected genes

**Supplementary Table** 5B: : Criteria for selection of non coding RNA based on pvalue

**Supplementary Table 1 :** The chemicals and drugs used in the study.

|  | **Company** | **CAS No.** |
| --- | --- | --- |
| **Urethane** | Sigma Aldrich | **51-79-6** |
| **Pentoxifylline** | Sigma Aldrich | **6493-05-6** |
| **Cyanidin 3-*O*-glucoside chloride** | Sigma Aldrich | **7084-24-4** |
| **Trans-Anethole** | Sigma Aldrich | **4180-23-8** |

**Supplementary Table 2:** Histopathological grading of the cardiac muscle

|  | **0** | **1** | **2** | **3** |
| --- | --- | --- | --- | --- |
| **Necrosis** | None or 1-3 dead cells in < 3 fields | ≤ 3 dead cell per field in at least 3 fields or 4-6 cells in no more than 3 fields | 4-6 dead cells per field in at least 4 fields or > 6 cells in no more than 3 fields | >6 dead cells in at least 4 fields |
| **Polymorpho-nuclear leucocytes** | None or 1-3 cells in < 3 fields | ≤ 3 cells per field in at least 3 fields or 4-6 cells in no more than 3 fields | 4-6 cells per field in at least 4 fields or> 6 cells in no more than 3 fields | >6 cells inn at least 4 fields |
| **Loss of cross**  **striation** | None or 1-5 cells in< 3 fields | ≤ 5 cells per field in at least 3 fields or 5-10 cells in no more than 3 fields | 5-10 cells per field in at least 4 fields or> 6 cells in no more than 3 fields | >10 cells in at least 4 fields |
| **Edema*** | None | <10% of fields in at least 3 fields or > 10% in < 3 fields | 10-30% of fields in at least 3 fields or> 30% in < 3 fields | >30% of fields in at least 3 fields |
| **Hemorrhage*** | None | Present in 10% of fields in at least 3 fields or > 10% in < 3 fields | Preset in 10-30% of fields in at least 3 fields or > 30% in< 3 fields | Present in > 30% of fields in at least 3 fields |

#### **Supplementary Table 3A Fibrosis Metrics Across Experimental Conditions**

| **Experimental Group** | **Fibrotic Area (% ± SEM)** | **Fibrotic Density (Intensity ± SEM)** | **p-value vs. MI/R** |
| --- | --- | --- | --- |
| **Sham Control** | 2.1 ± 0.3 | 38.2 ± 4.1 | — |
| **MI/R Control** | 42.7 ± 3.1 | 182.6 ± 11.4 | — |
| **MI/R + Cy3G-Low (10 mg/kg)** | 28.5 ± 2.4 | 135.3 ± 9.8 | <0.01 |
| **MI/R + Cy3G-High (30 mg/kg)** | 18.9 ± 1.9 | 98.7 ± 8.2 | <0.001 |
| **MI/R + TNA 50 mg/kg** | 34.2 ± 2.6 | 155.1 ± 9.3 | <0.05 |
| **MI/R + TNA 100 mg/kg** | 26.8 ± 2.0 | 129.6 ± 8.5 | <0.01 |
| **MI/R + TNA 200 mg/kg** | 19.3 ± 1.8 | 102.4 ± 7.6 | <0.001 |
| **MI/R + PTX 20 mg/kg** | 31.7 ± 2.5 | 148.9 ± 8.9 | <0.05 |
| **MI/R + PTX 30 mg/kg** | 24.6 ± 1.9 | 121.8 ± 7.3 | <0.01 |
| **MI/R + PTX 40 mg/kg** | 20.5 ± 1.7 | 106.2 ± 6.8 | <0.001 |

#### **Supplementary Table S3B Comparative Efficacy of the drugs on fibrosis**

| **Drug (Optimal Dose)** | **Fibrosis Reduction** | **vs. Cy3G-High** |
| --- | --- | --- |
| Cy3G-High (30 mg/kg) | ↓55.7% | — |
| TNA 200 mg/kg | ↓54.8% | >0.05 |
| PTX 40 mg/kg | ↓52.0% | <0.05 |

**Supplementary Table S4. Details of the GSE61592 and GSE14843 datasets were retrieved from the GEO database.**

|  |  |  |  |  |
| --- | --- | --- | --- | --- |
| GSE61592 | [Mus musculus](https://www.ncbi.nlm.nih.gov/Taxonomy/Browser/wwwtax.cgi?mode=Info&id=10090) | [GPL6887](https://www.ncbi.nlm.nih.gov/geo/query/acc.cgi?acc=GPL6887)Illumina MouseWG-6 v2.0 expression beadchip. | Expression profiling by array | Group 1 is wild type C57Bl6 uninjured hearts. These mice were not undergone any surgery and used as controls. Group 2 are wild type C57Bl6 72 h post-ischemia reperfusion (IR) injury hearts. These mice for subjected to ischemia reperfusion (IR) involving 90 min of left anterior descending coronary artery occlusion followed by reperfusion for 3 days or 72 h. |
| GSE14843 | [Mus musculus](https://www.ncbi.nlm.nih.gov/Taxonomy/Browser/wwwtax.cgi?mode=Info&id=10090) | GPL8224 Kanazawa University/Hitachisoft AceGene Mouse Oligo Chip 30k 1 Chip Version | Expression profiling by array | C57BL/6J mice (n=46, BW 24.1±1.42 g, 8–10 weeks of age; Charles River Laboratories) were divided into the following treatment groups: sham-operated (n=11), I/R (n=10), myocardial infarction (n=10), liver I/R (n=10) |

- Table S5A: showing binding scores, seed match between miRNAs with the selected genes

|  |  | **DCN** | **SOX5** | **VAV3** |
| --- | --- | --- | --- | --- |
| miR-1273a | binding scores | **0.923** | **0.923** | **0.923** |
|  | seed match | **1** | **0** | **1** |
| miR-1298 | binding scores | **1** | **0.923** | **0.923** |
|  | seed match | **1** | **1** | **0** |
| miR-133 | binding scores | **1** | **1** | **0.846** |
|  | seed match | **1** | **0** | **0** |

- Table S5B : criteria of selection of non coding RNA based on pvalue:

|  | miR-1273 | miR-1298 | miR-133a-3p |
| --- | --- | --- | --- |
| LINC00654 | 0.024 | 0.001 | 0.024 |
| JRKL-AS1 | 0.042 |  |  |
| CTC-448F2.4 | 0.005 |  |  |
