# Supplementary material for: Machine learning-based prediction of drug response in ischemia reperfusion animal model
Source: Sci Rep. 2025 Dec 2;15:43012. doi: 10.1038/s41598-025-18620-8 (PMC12672590; doi:10.1038/s41598-025-18620-8)

**Figure S1. Gene Ontology using Genecards**

SOX5


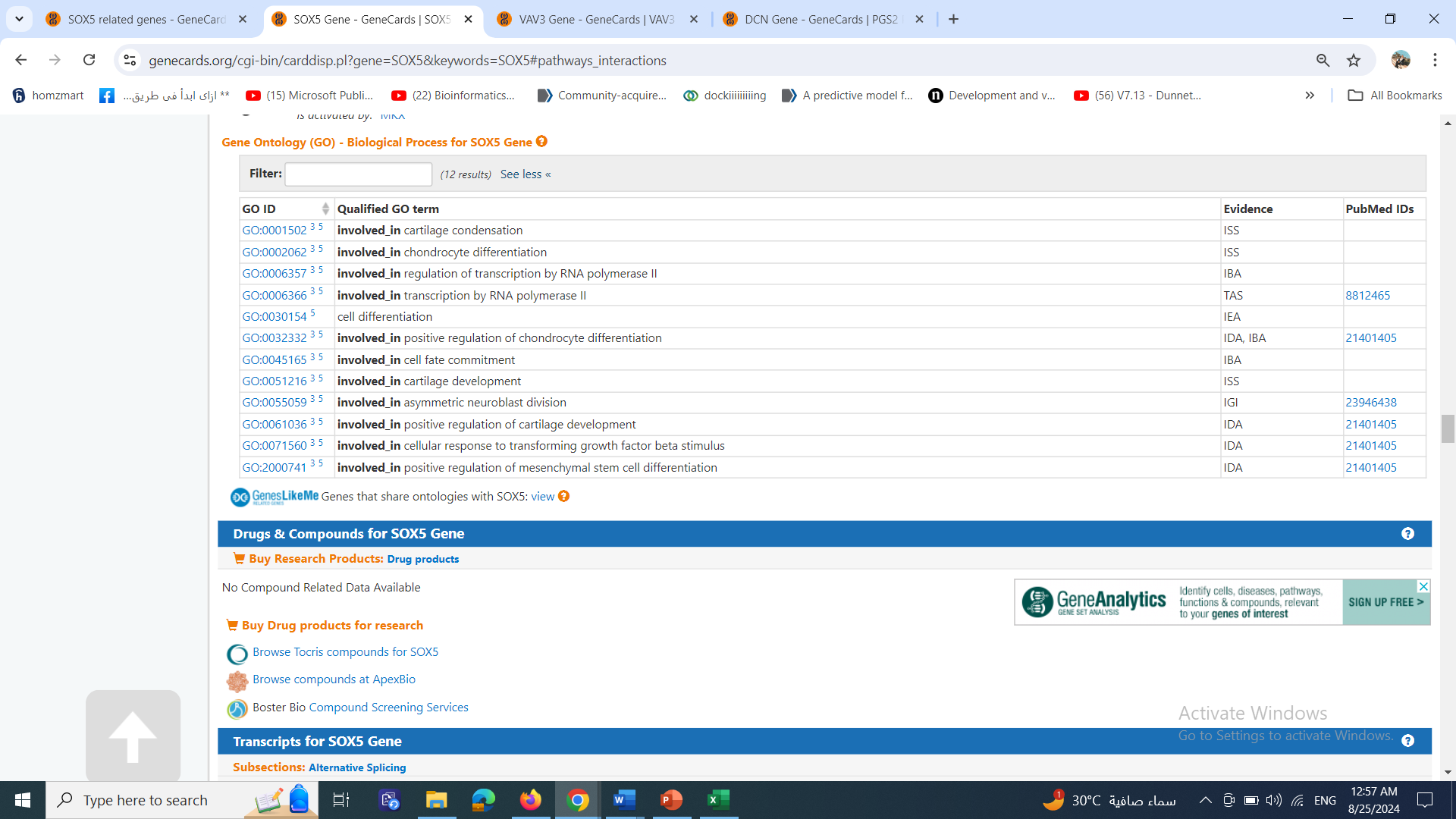


VAV3


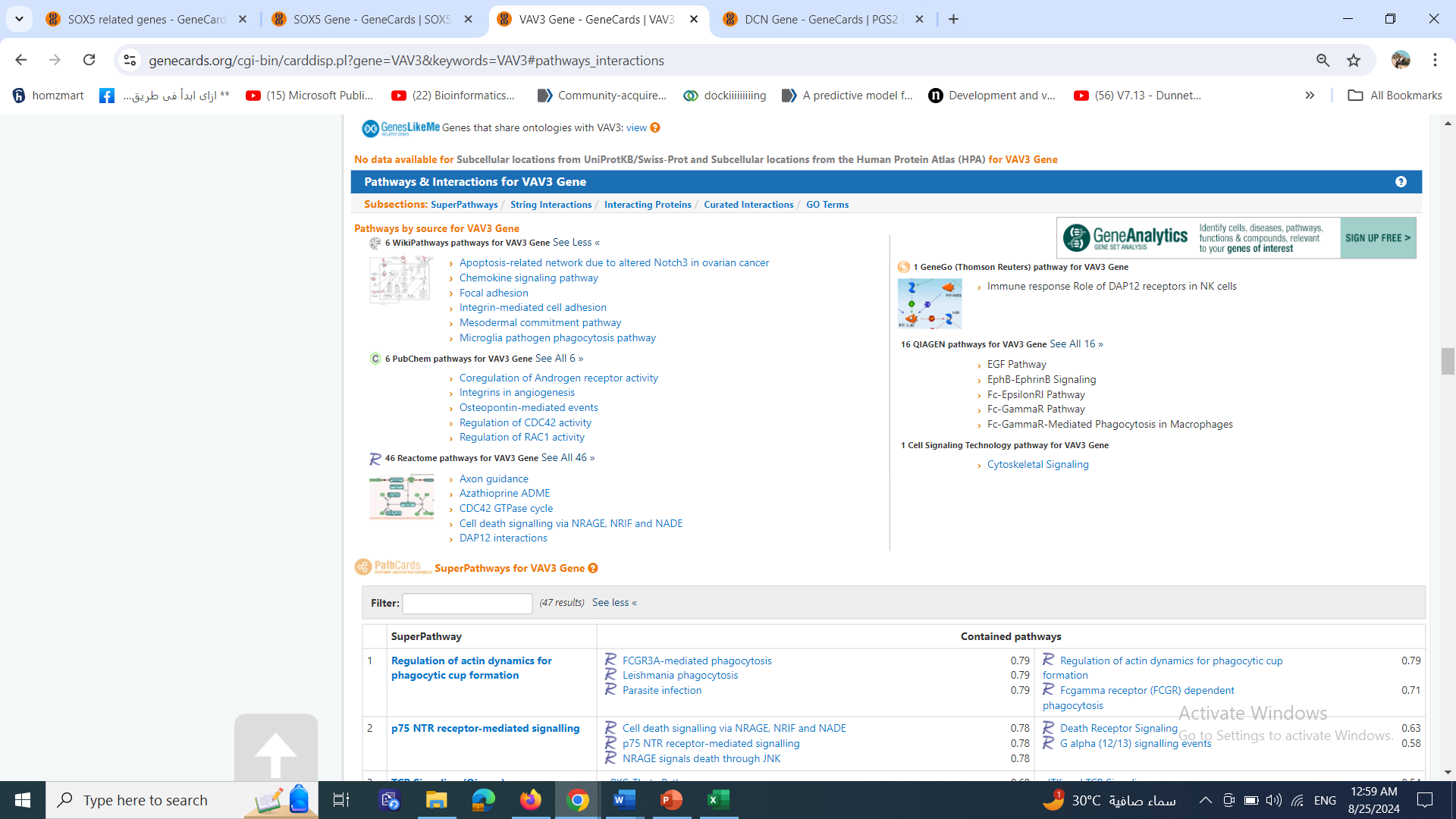


DCN


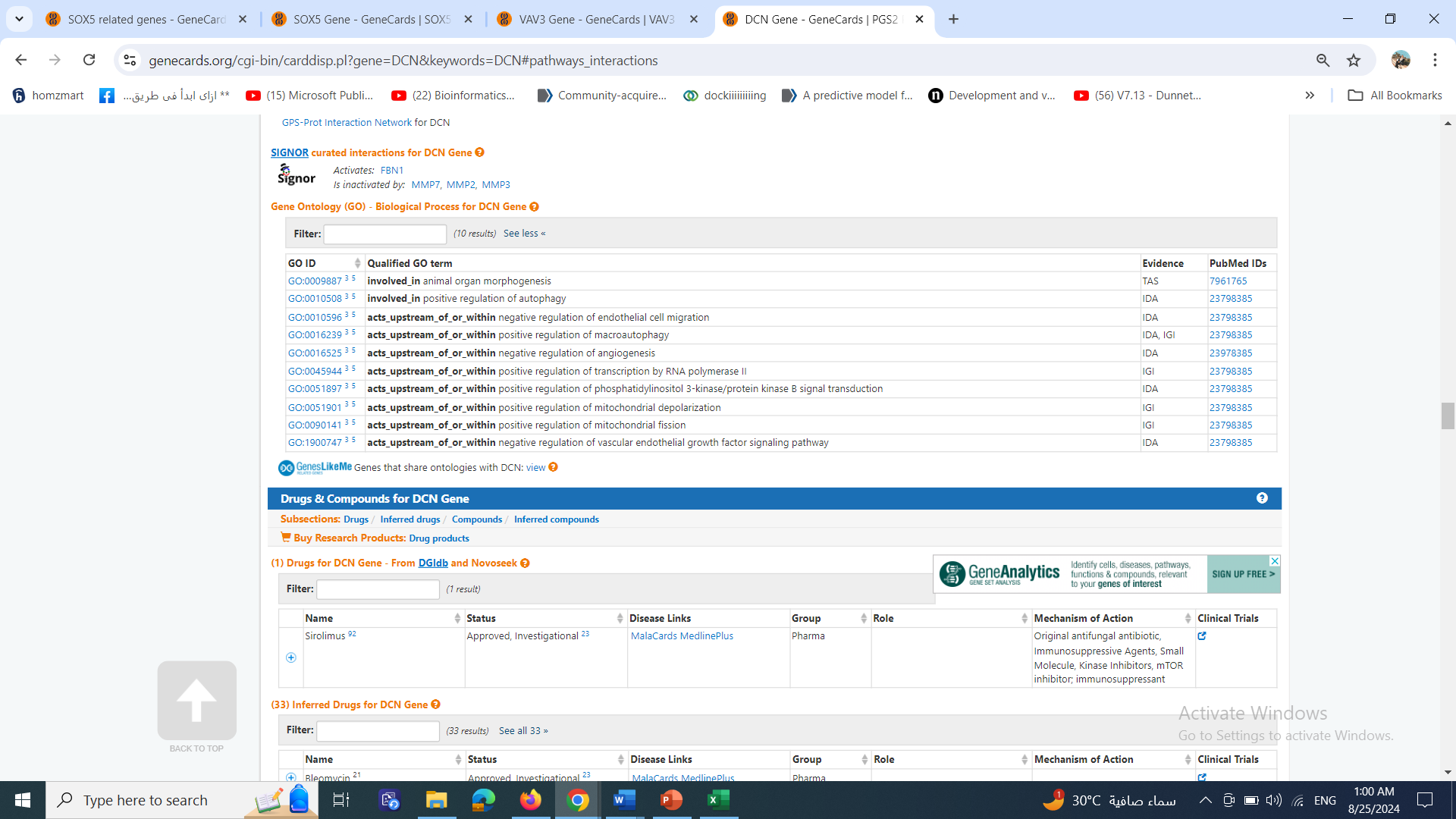


**Figure S2.**Validation of the interaction between the selected mRNAs and the retrieved miRNAs from the mirWalk (<http://mirwalk.umm.uni-heidelberg.de/>) and RNA22 tool (<https://cm.jefferson.edu/>)


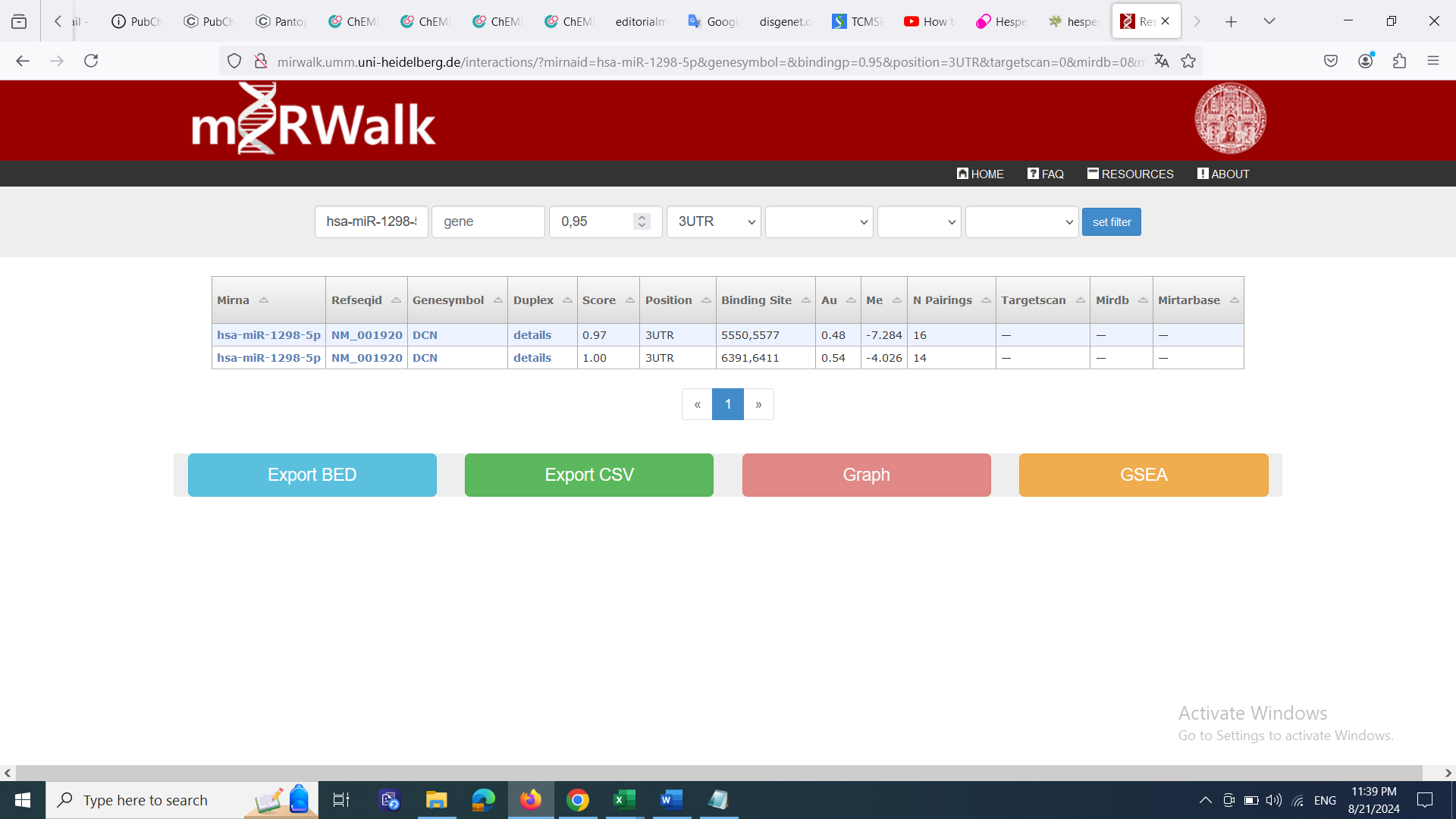


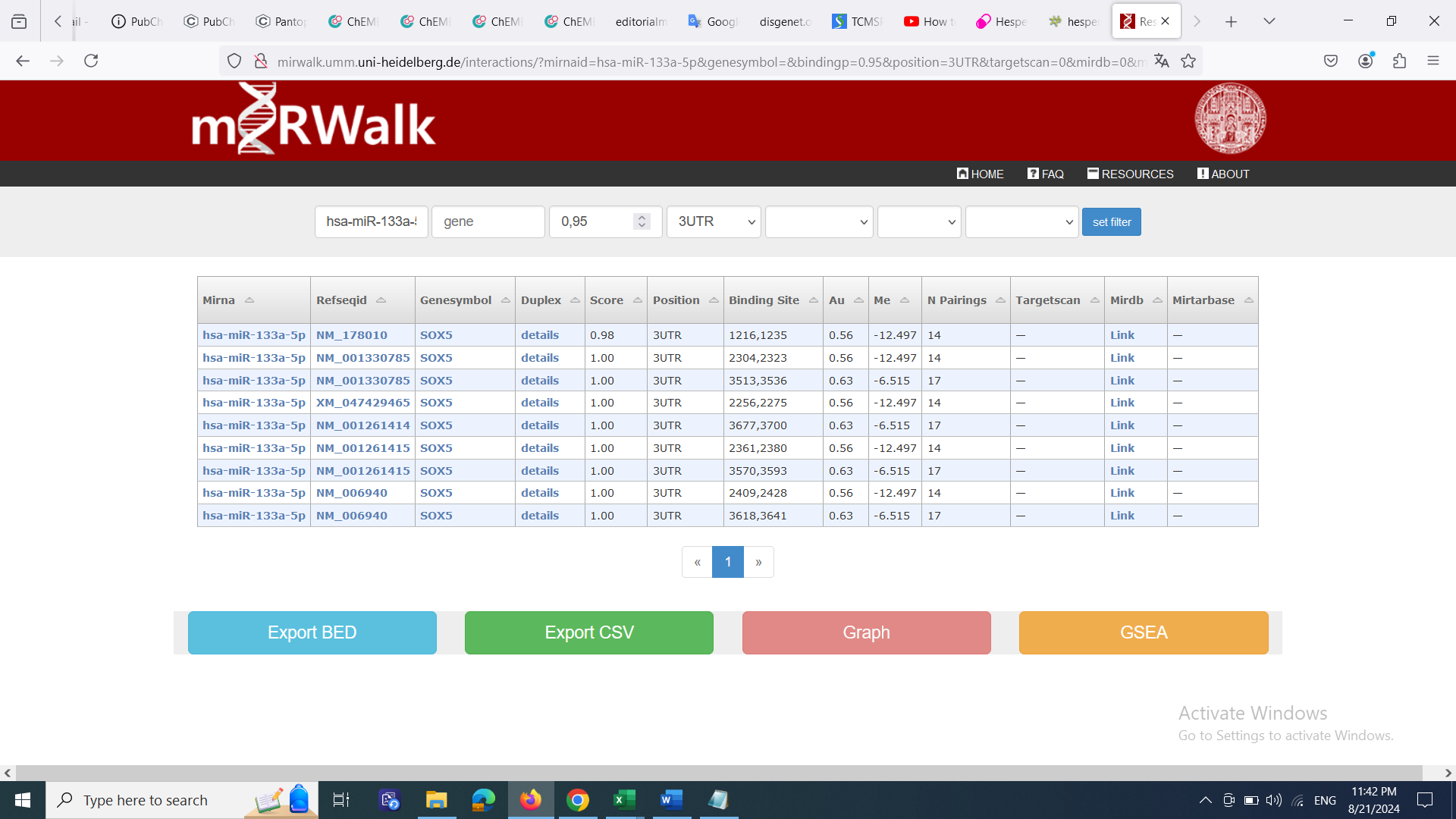


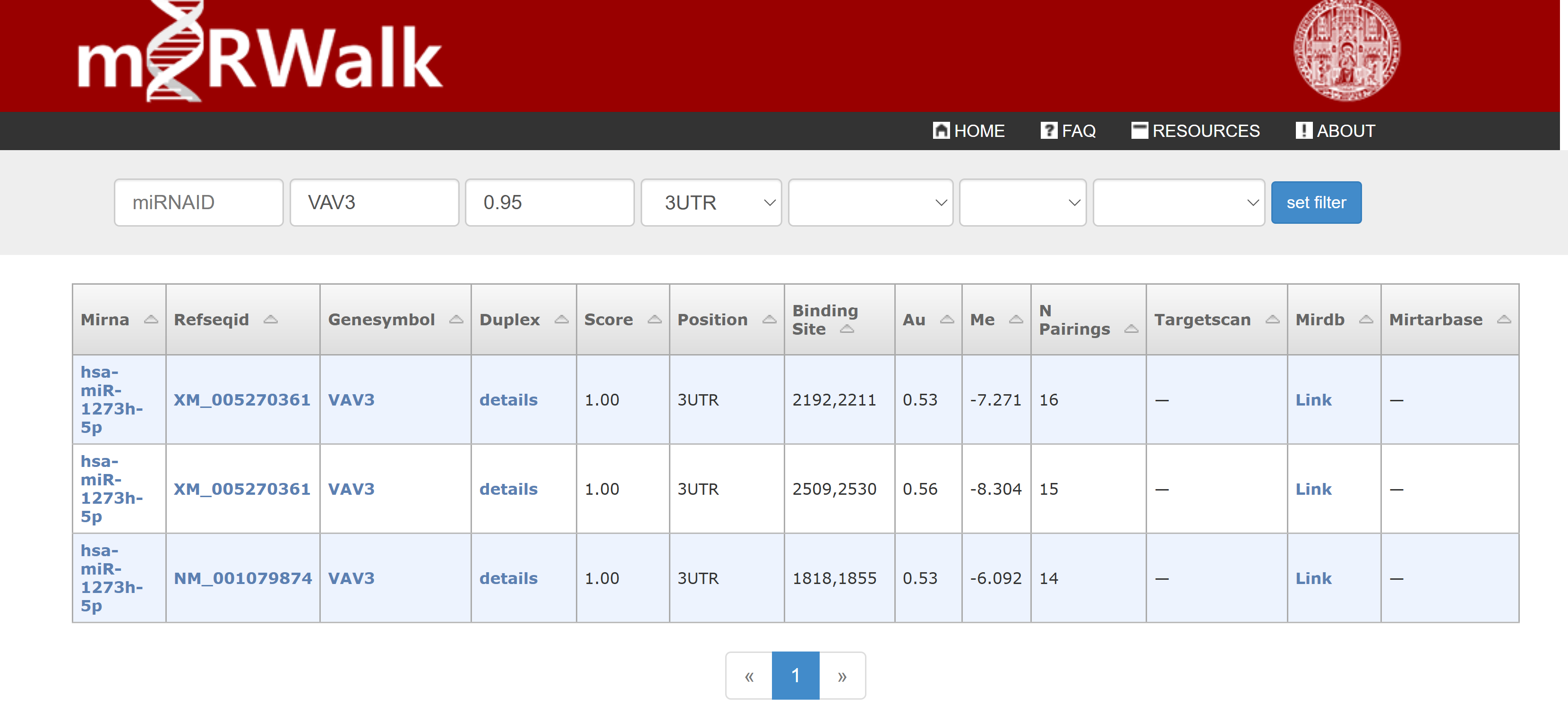


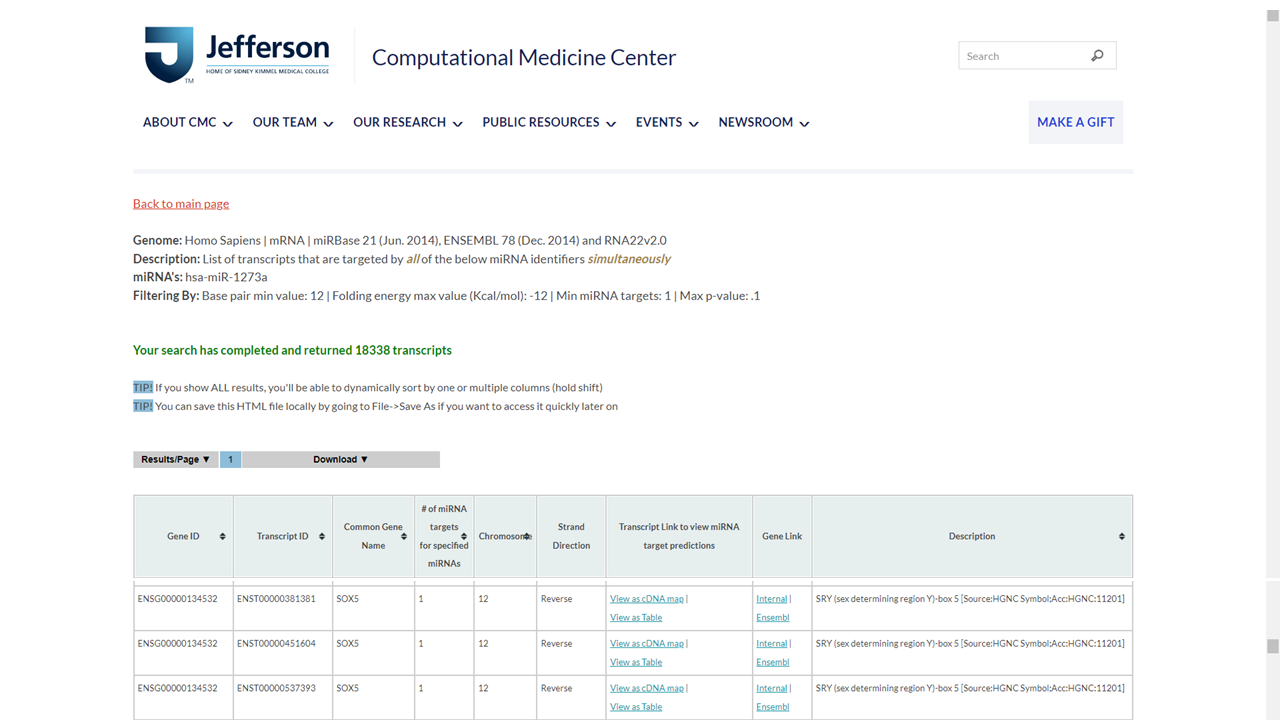


**Figure S3:** Validation of the interaction between the retrieved miRNAs and LncRNAs by RNA22 (<https://cm.jefferson.edu/rna22/>).

**Lnc-RNA-JRKL-AS1&hsa-miR-1273a**


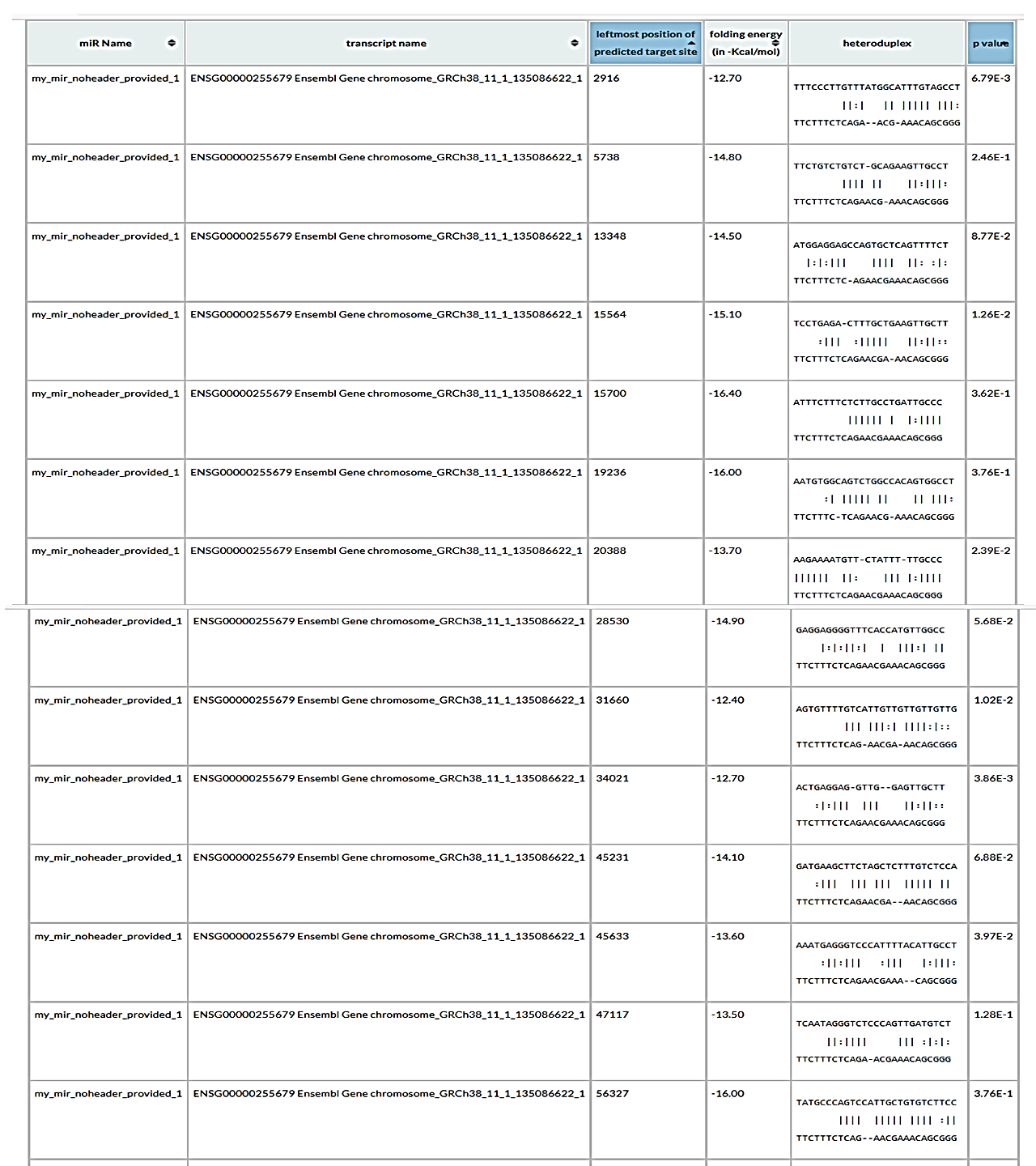


**Lnc-RNA-JRKL-AS1**&**hsa-miR-1298-5p**


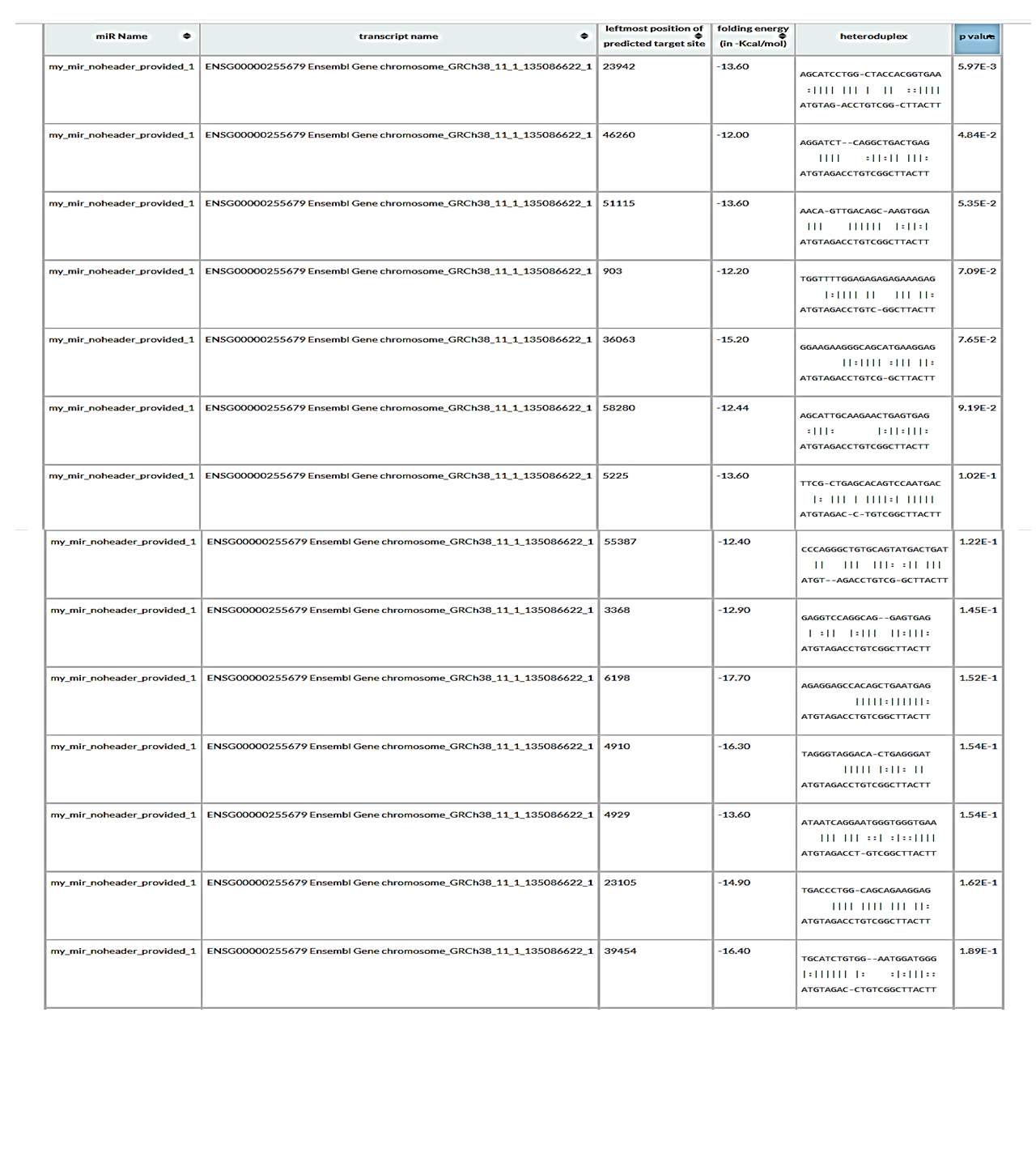


**Lnc-RNA-JRKL-AS1 & hsa-miR-133a-3p**


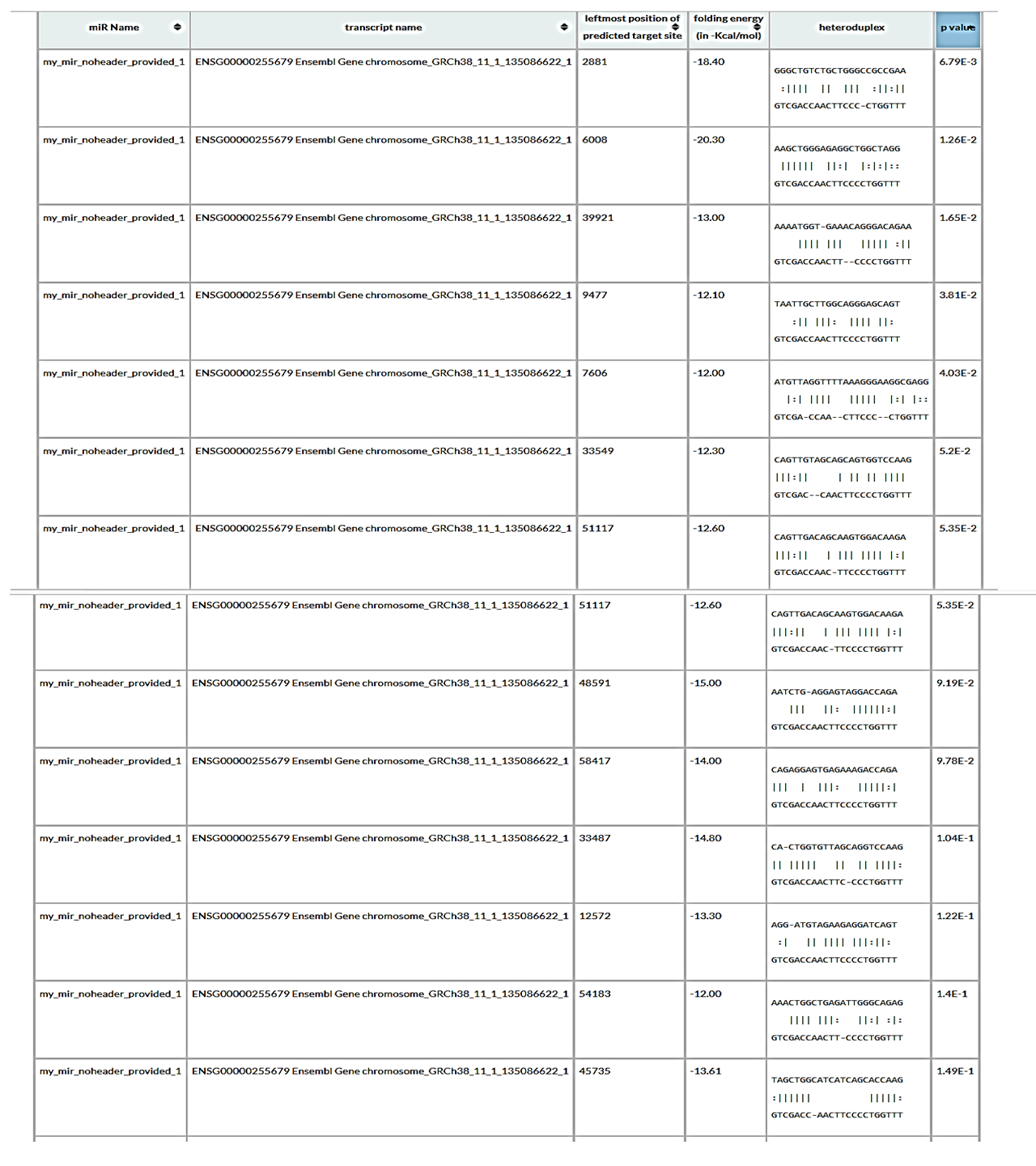


**LINC00654 &** **miR-133a-3p**


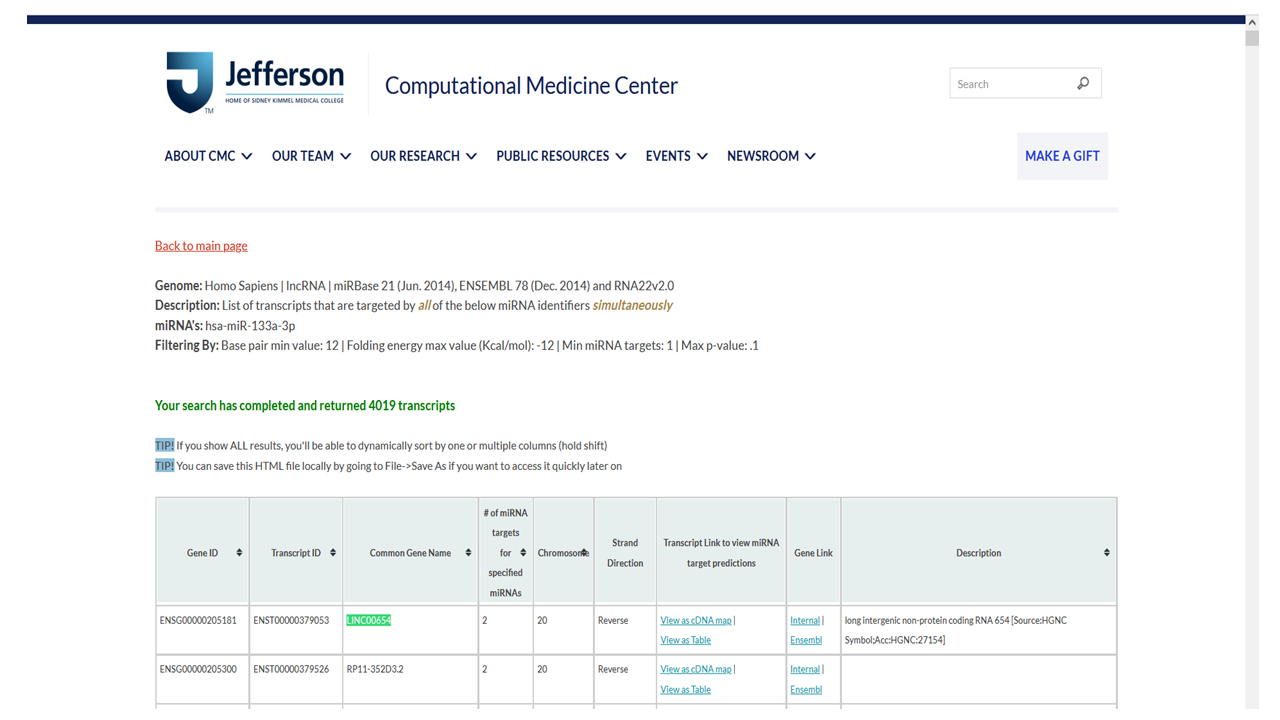


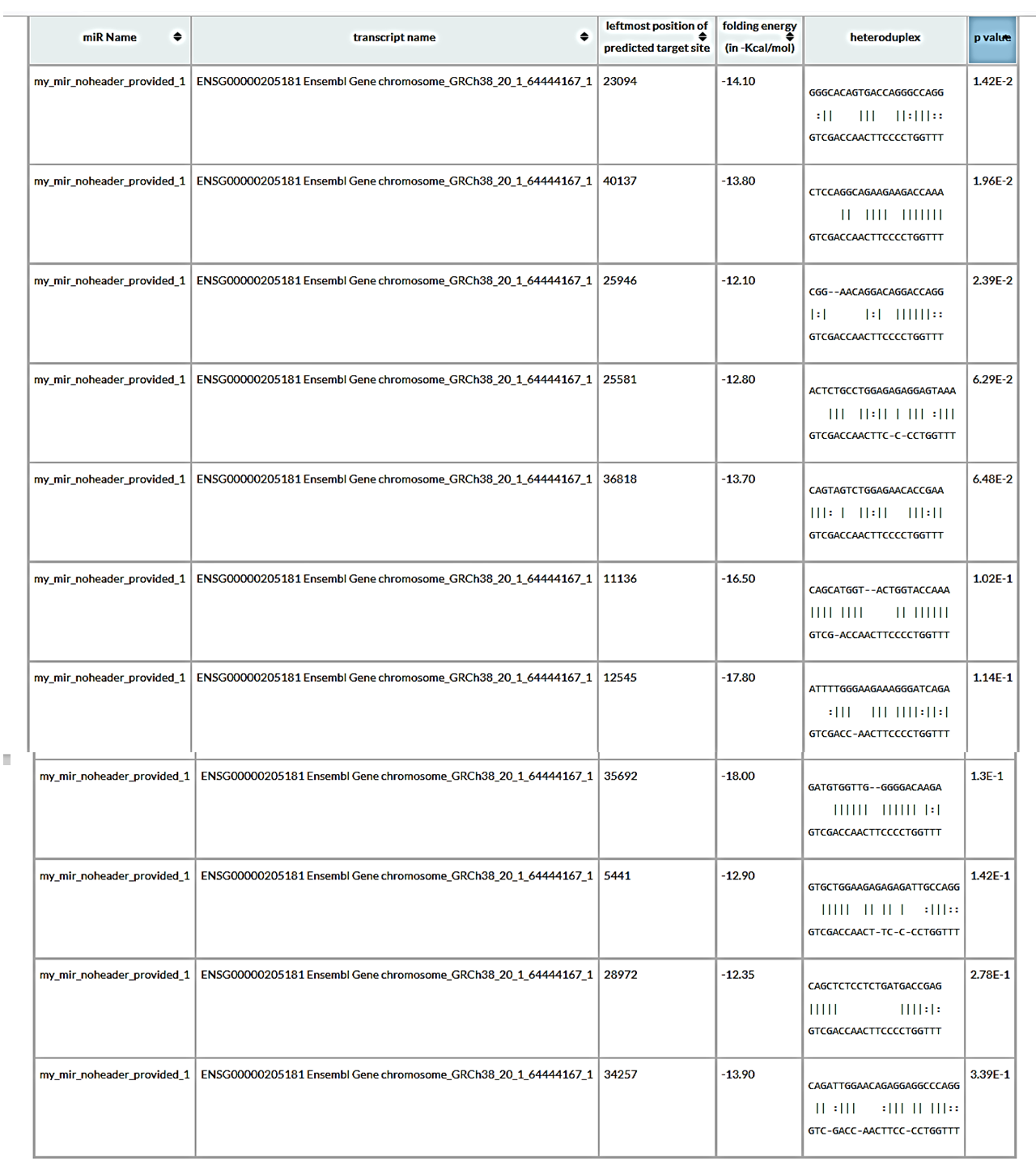


**LINC00654 & miR-1298**


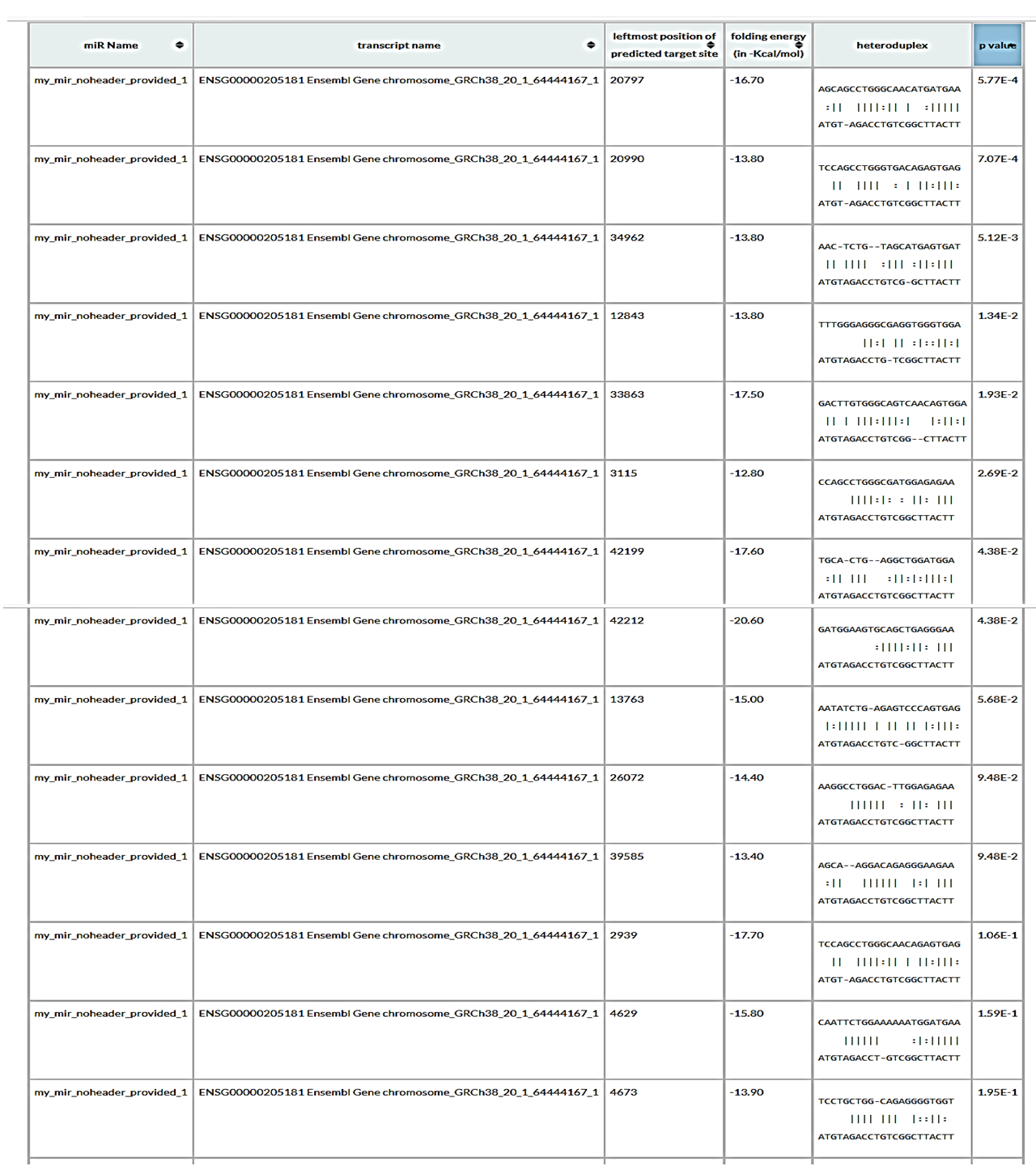


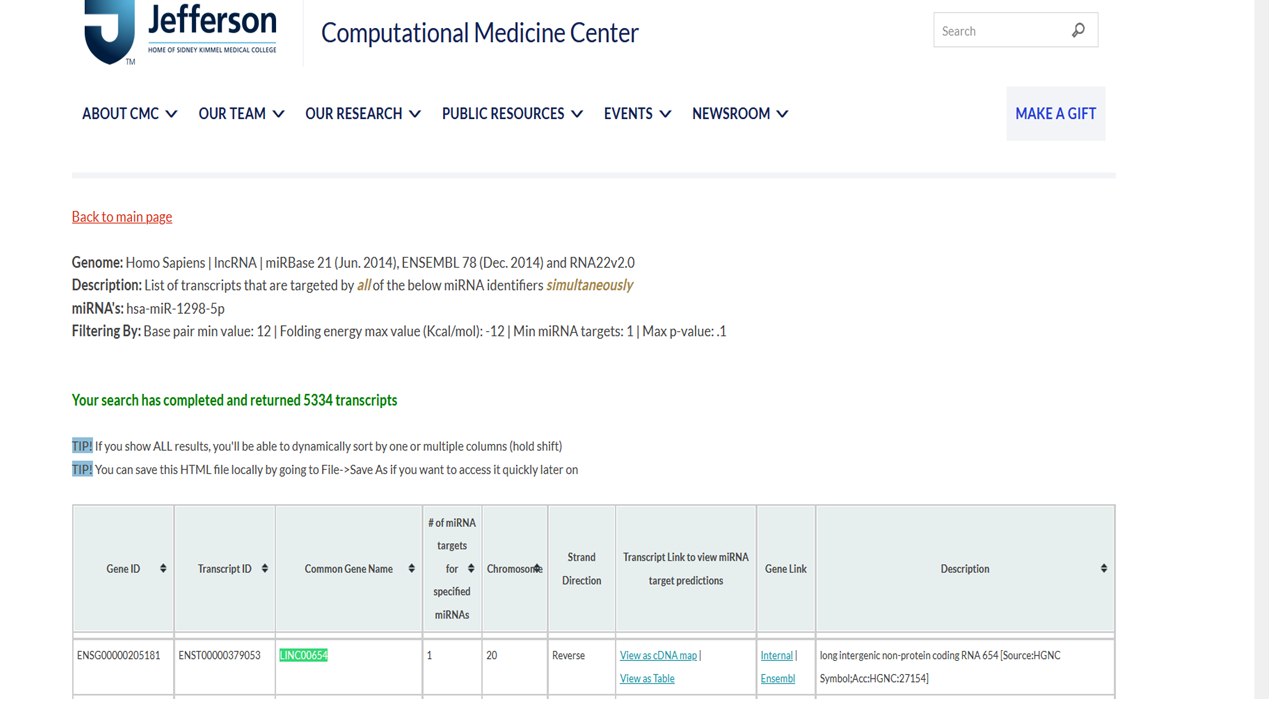


**LINC00654&** **miR-1273a**


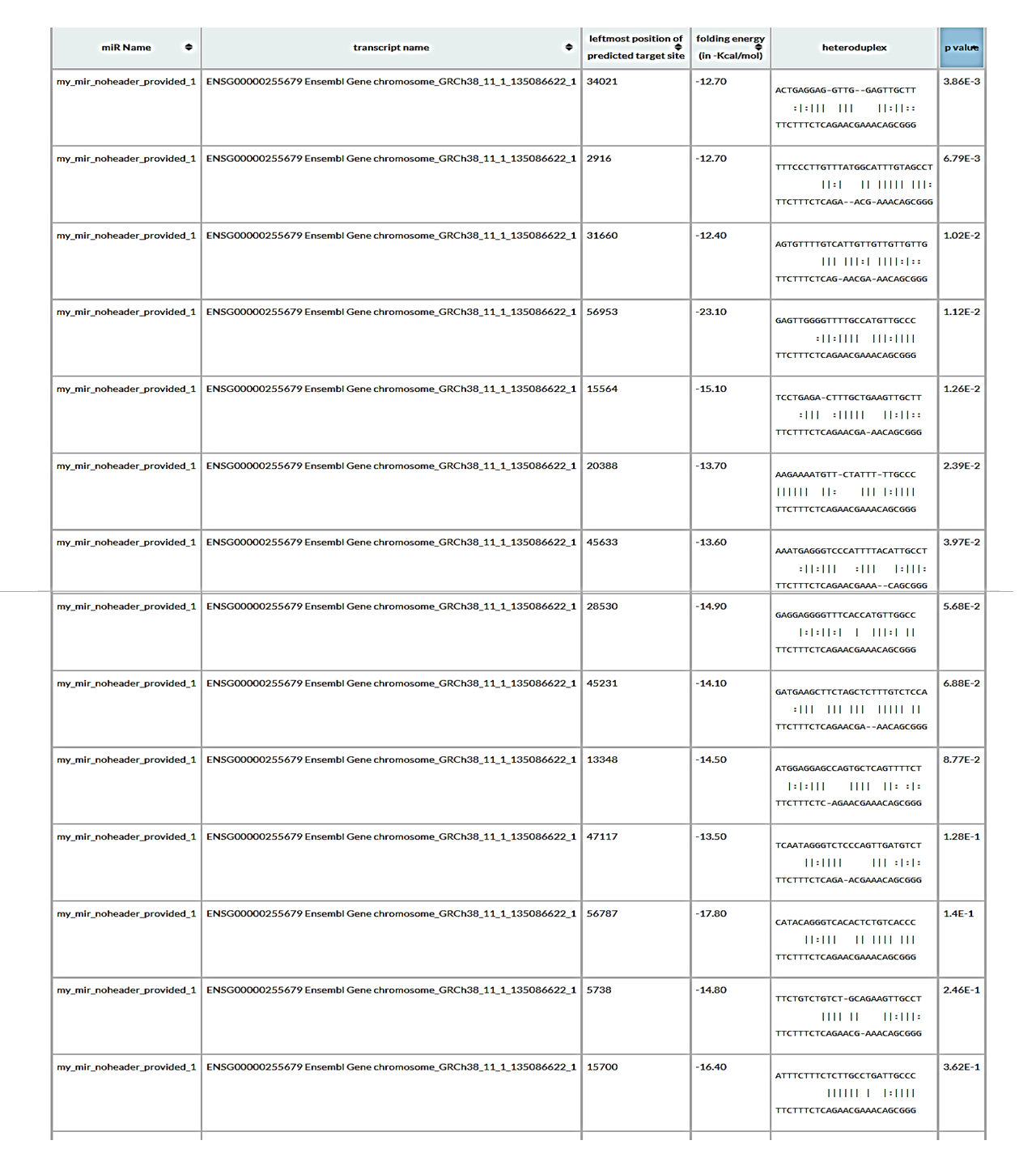


**Lnc-CTC-448F2.4 & hsa-miR-1298-5p**

**
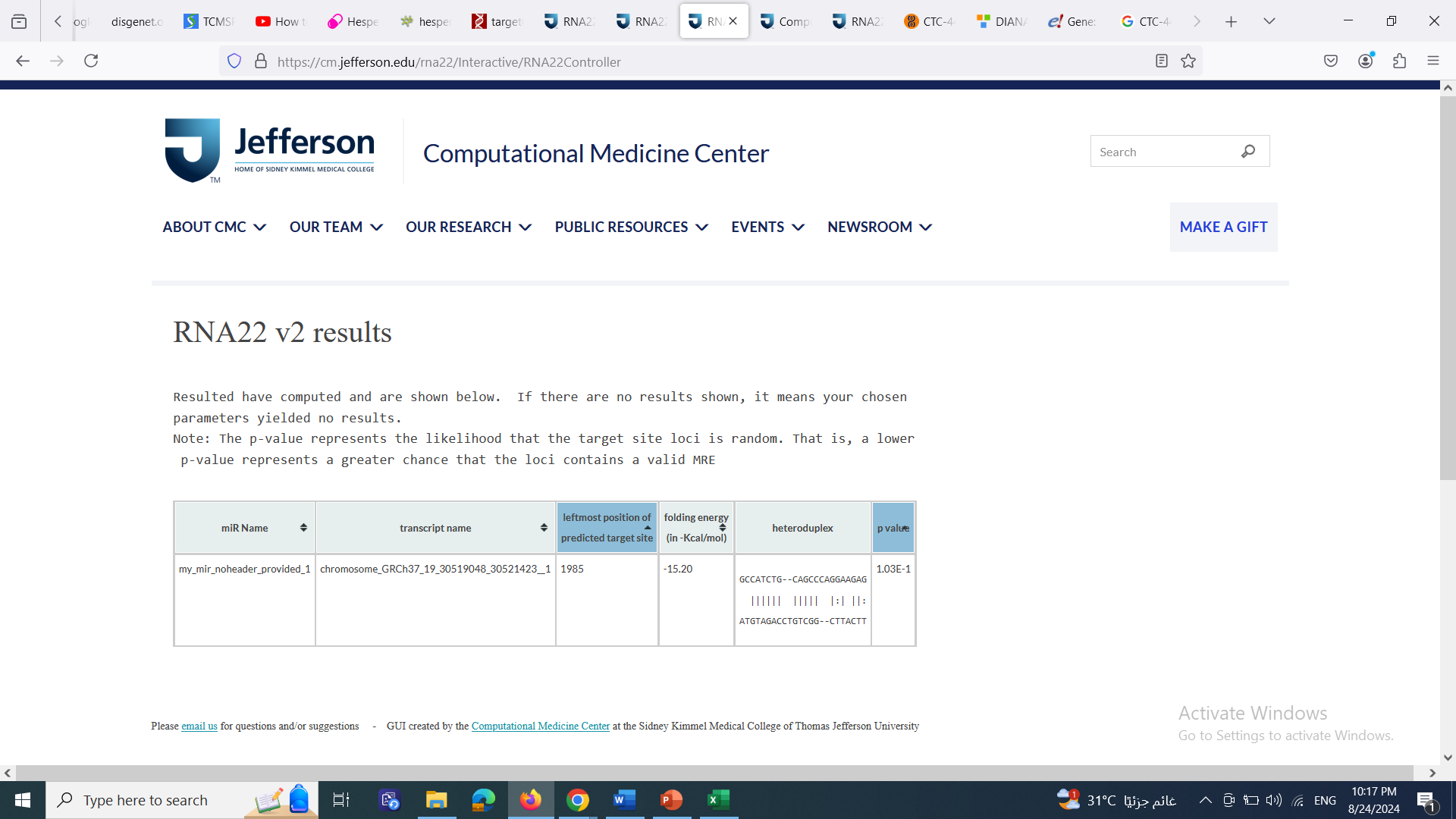
**

**Figure S4: Stability of U6 Expression Across Treatment Groups in the supplementary materials.**

**
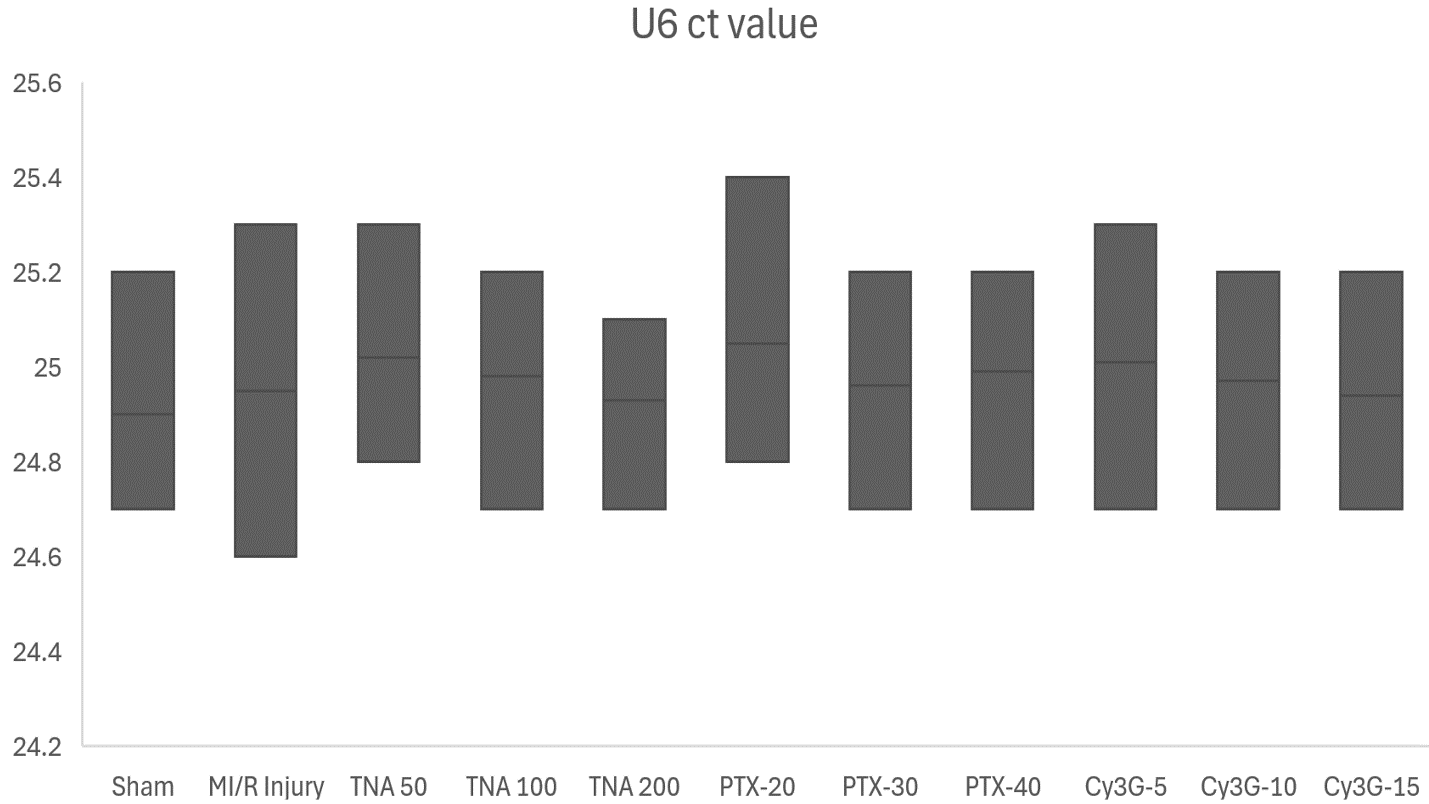
**

- **Visual Format: Box plots showing U6 Ct values for all groups.**
- **Key Annotations**:
  - Horizontal line = median Ct value.
  - Whiskers = interquartile range (IQR).
  - *P*-value from ANOVA (ns = not significant).


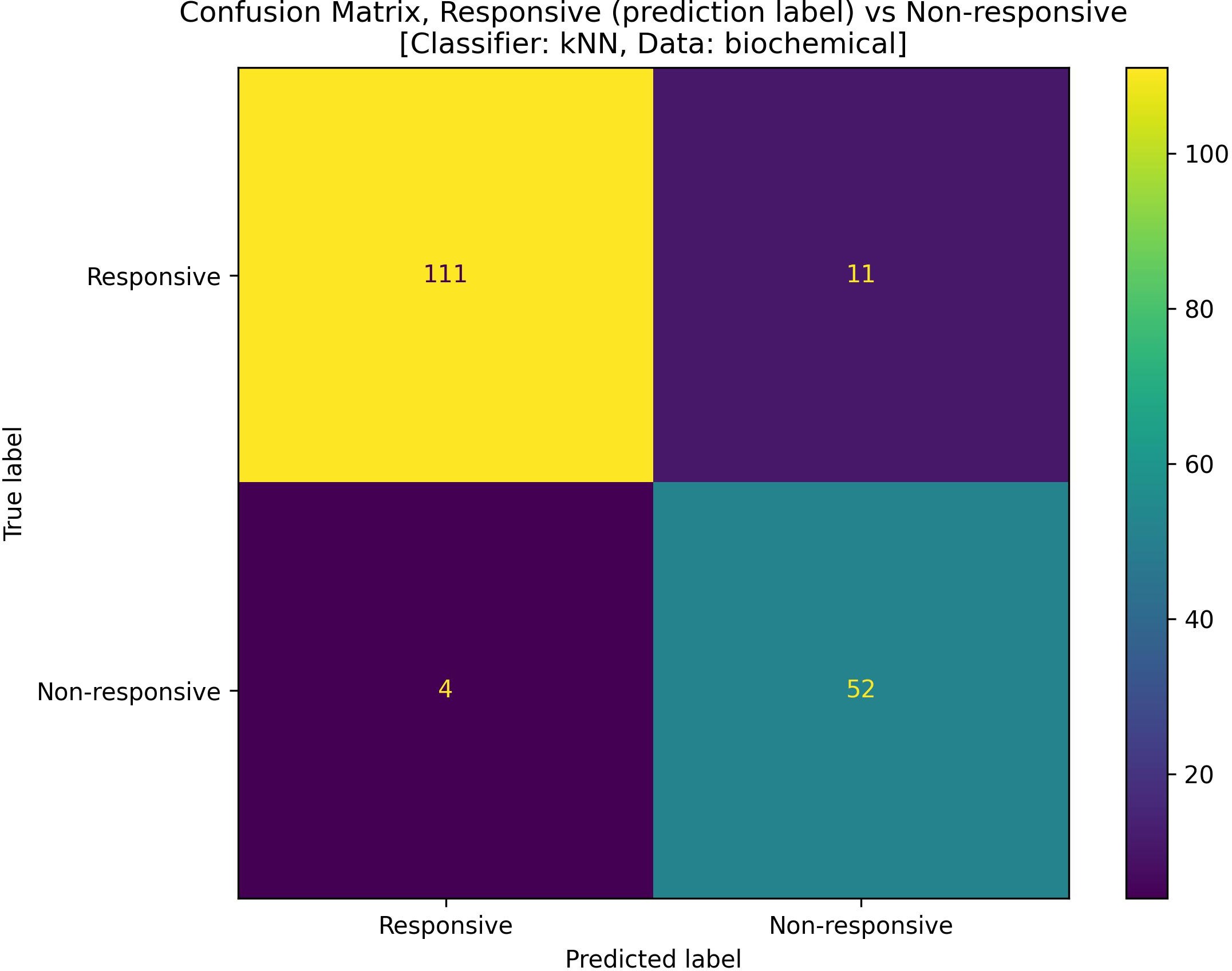
**Figure S5. Confusion Matrices**


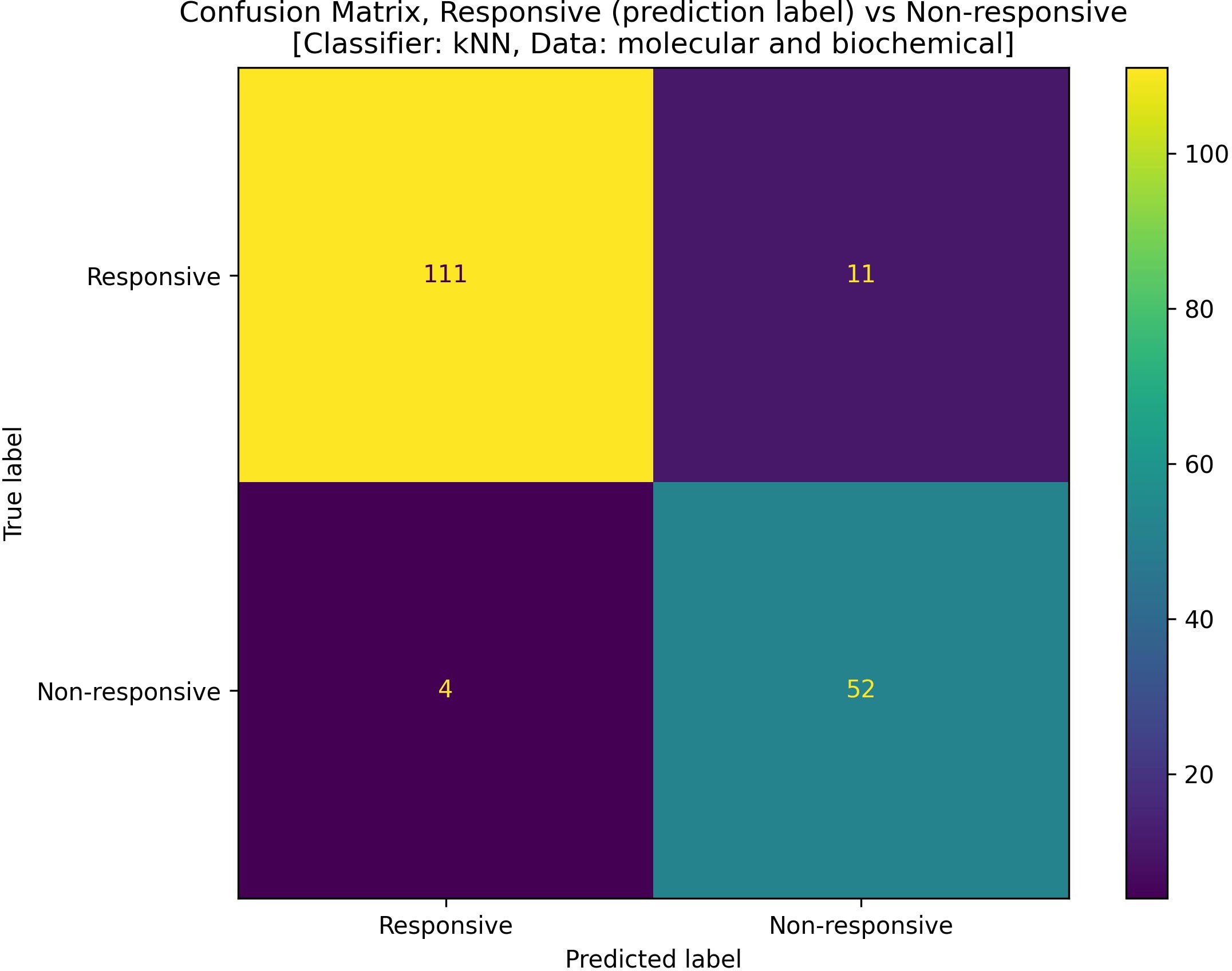


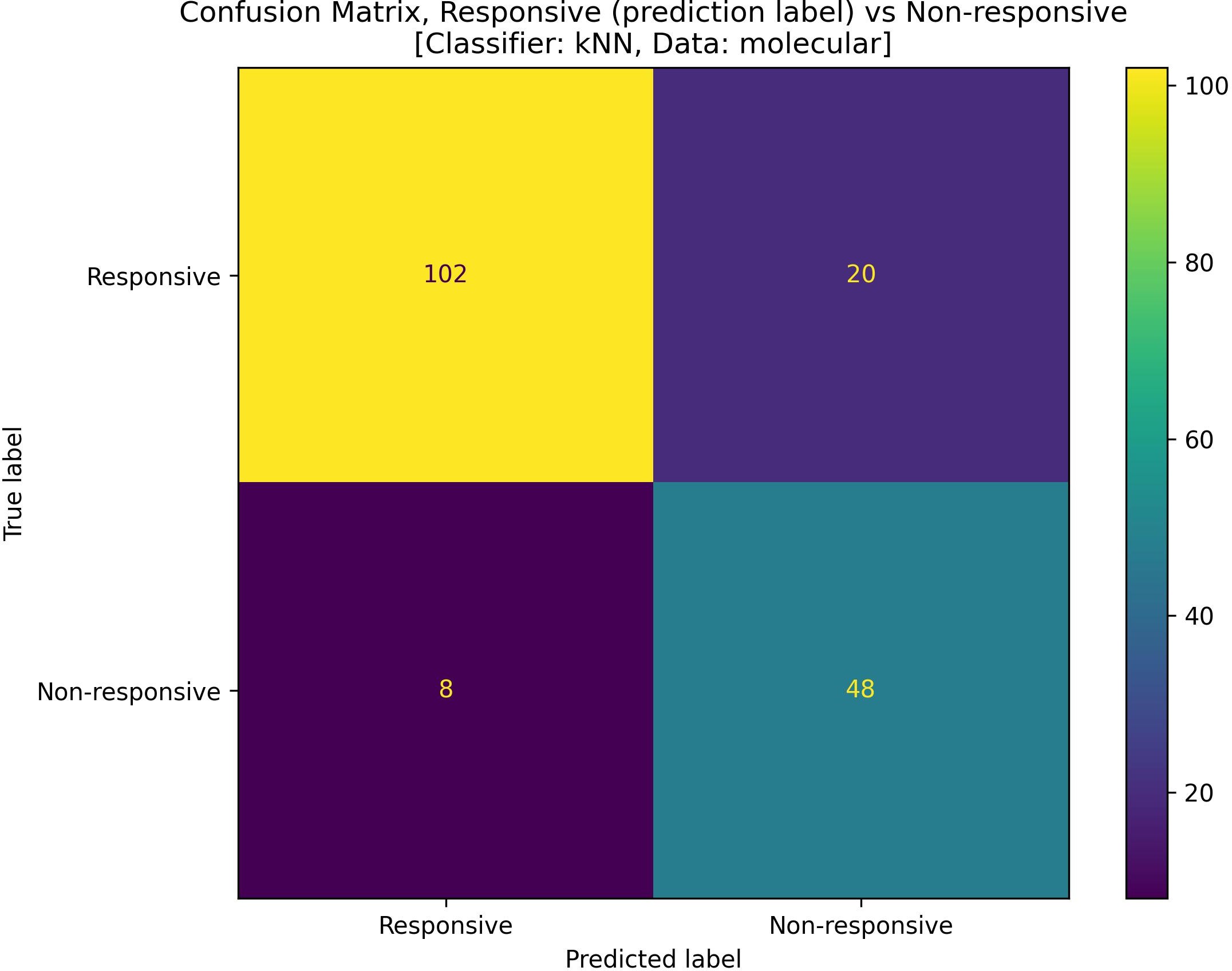


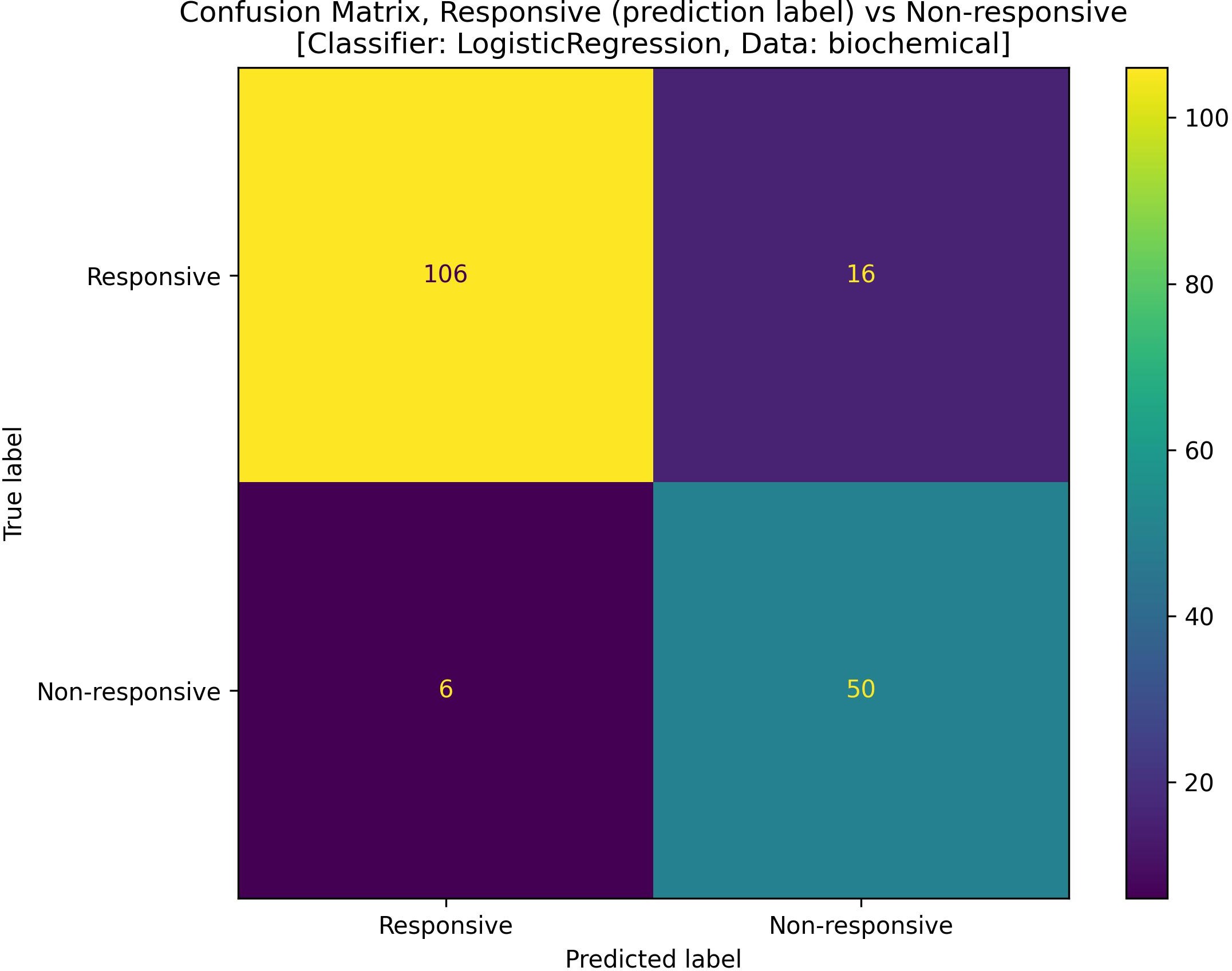


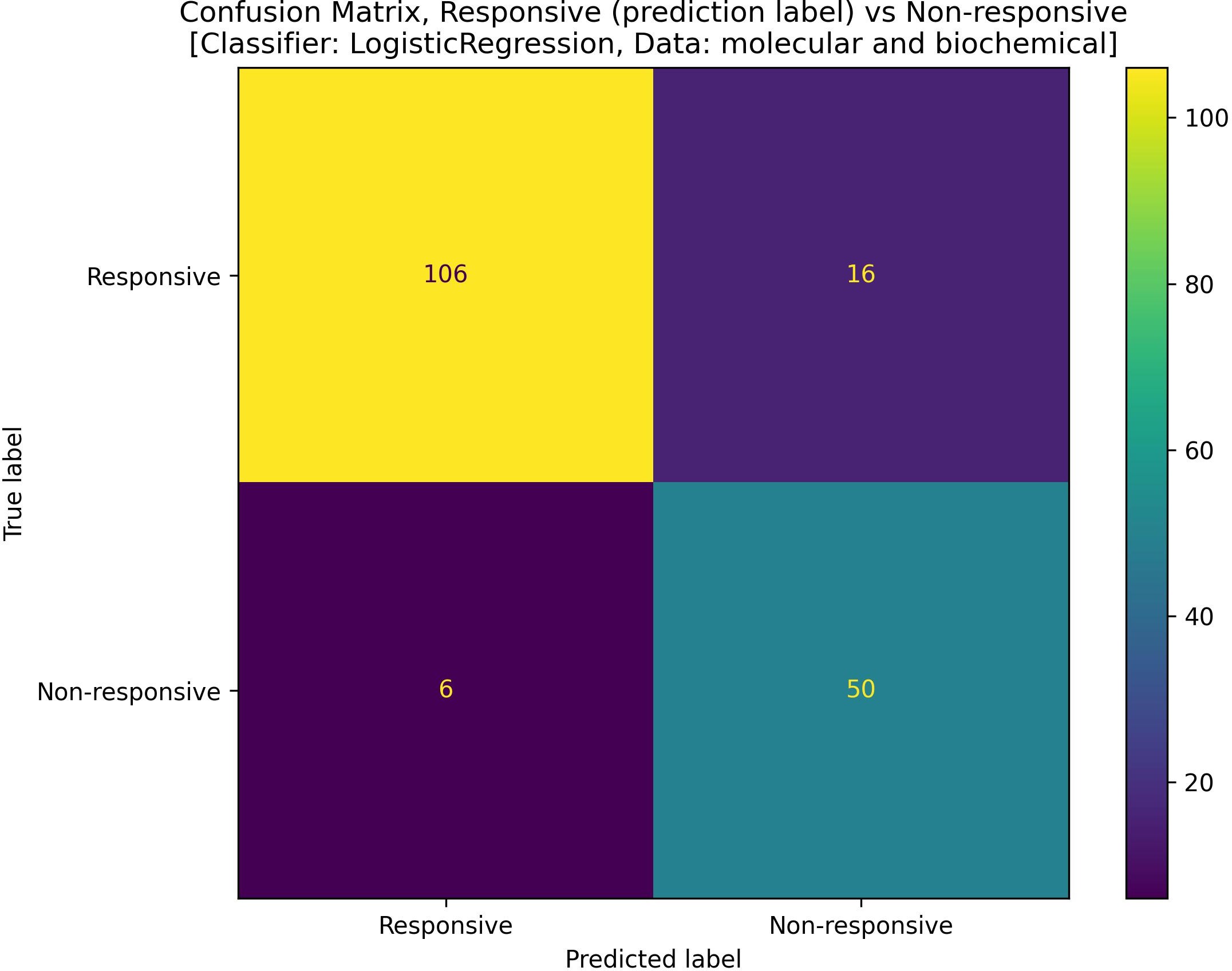


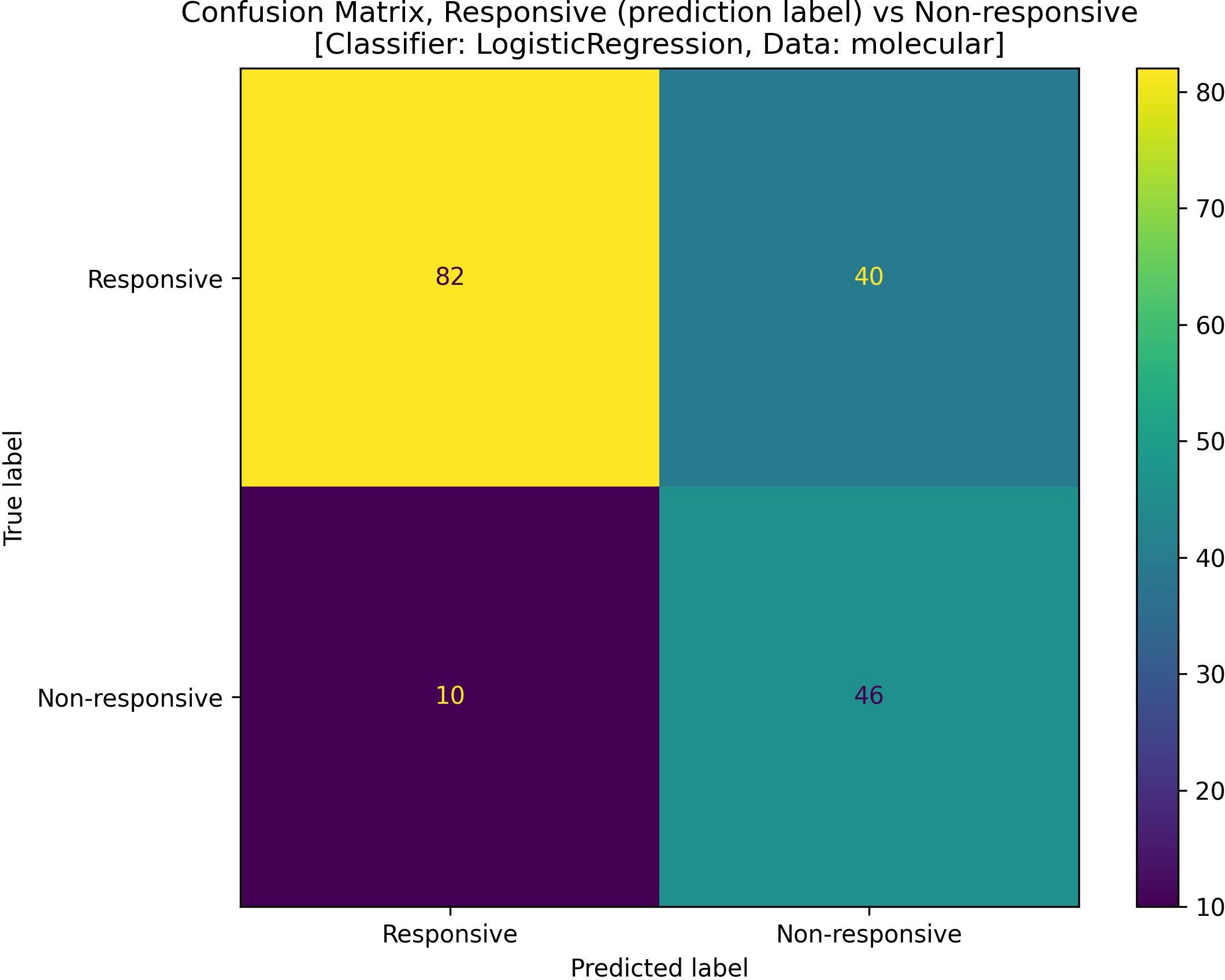


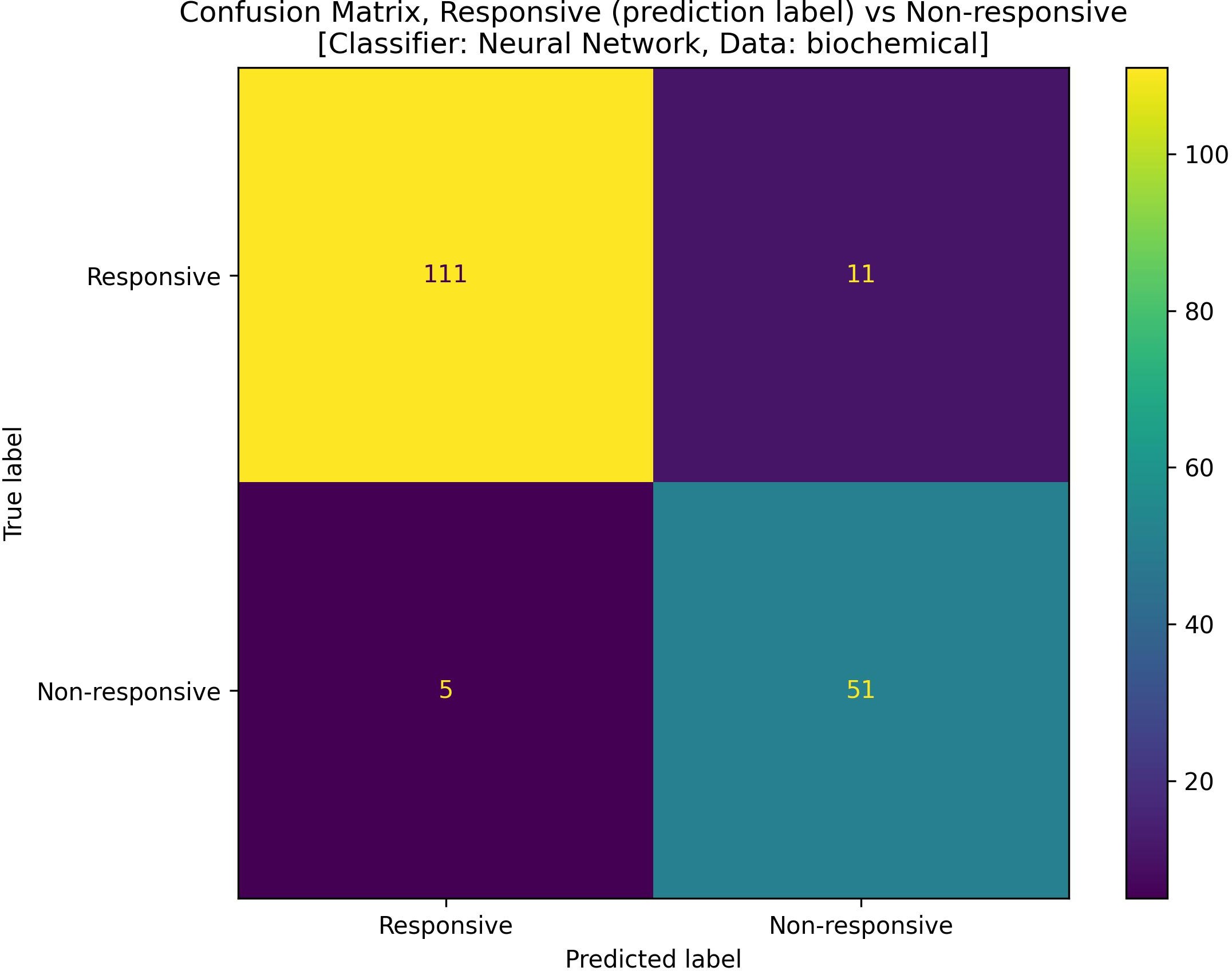


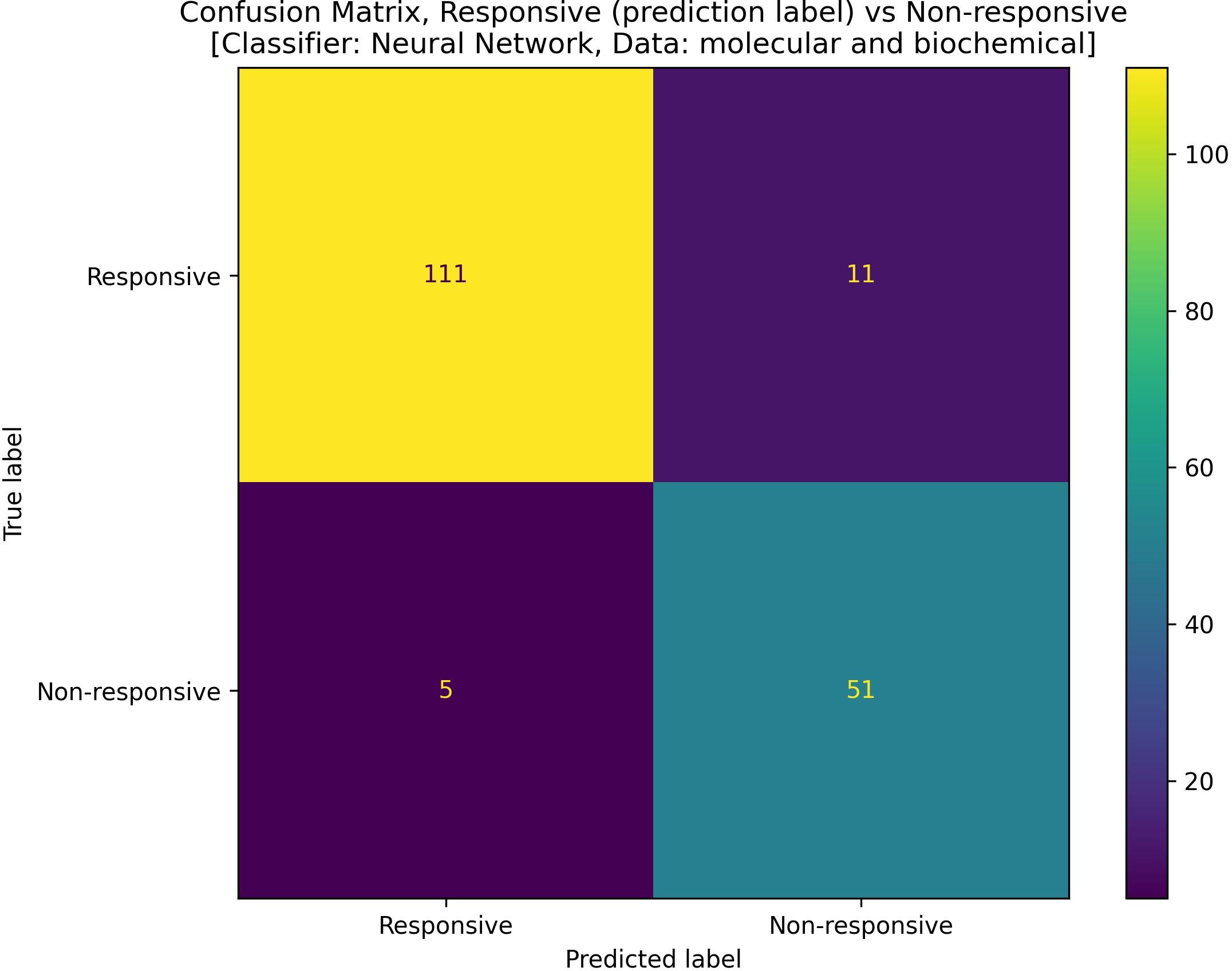


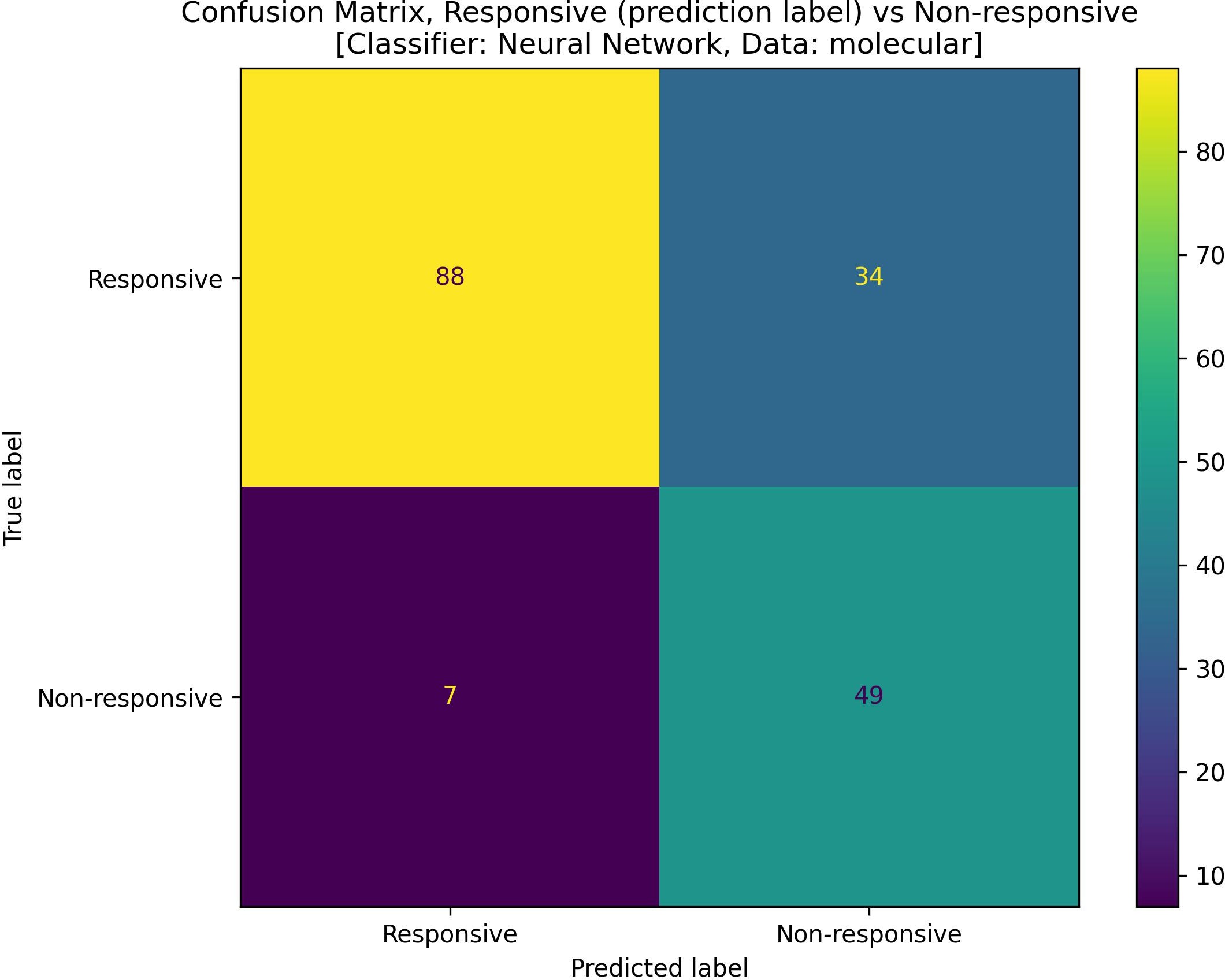


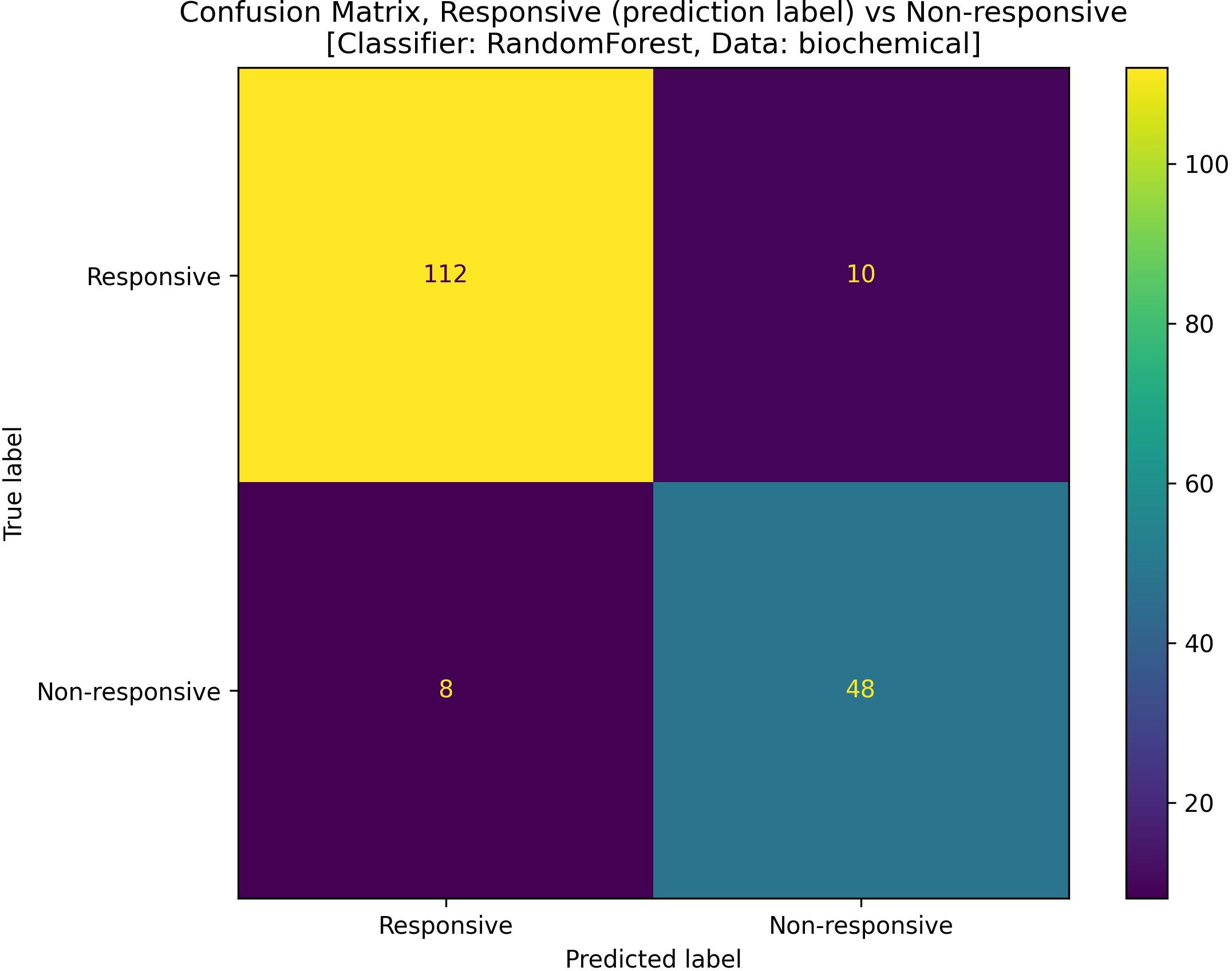


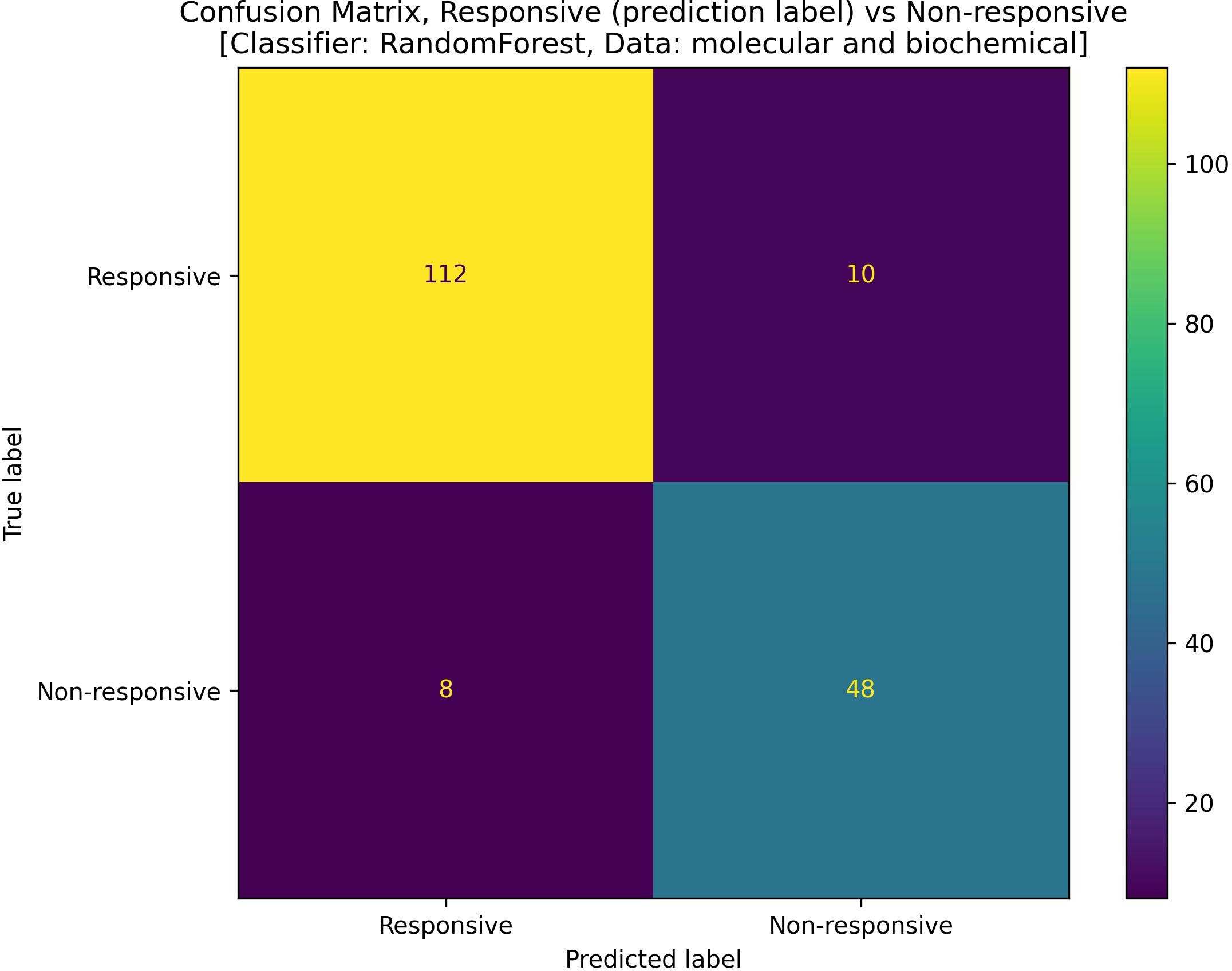


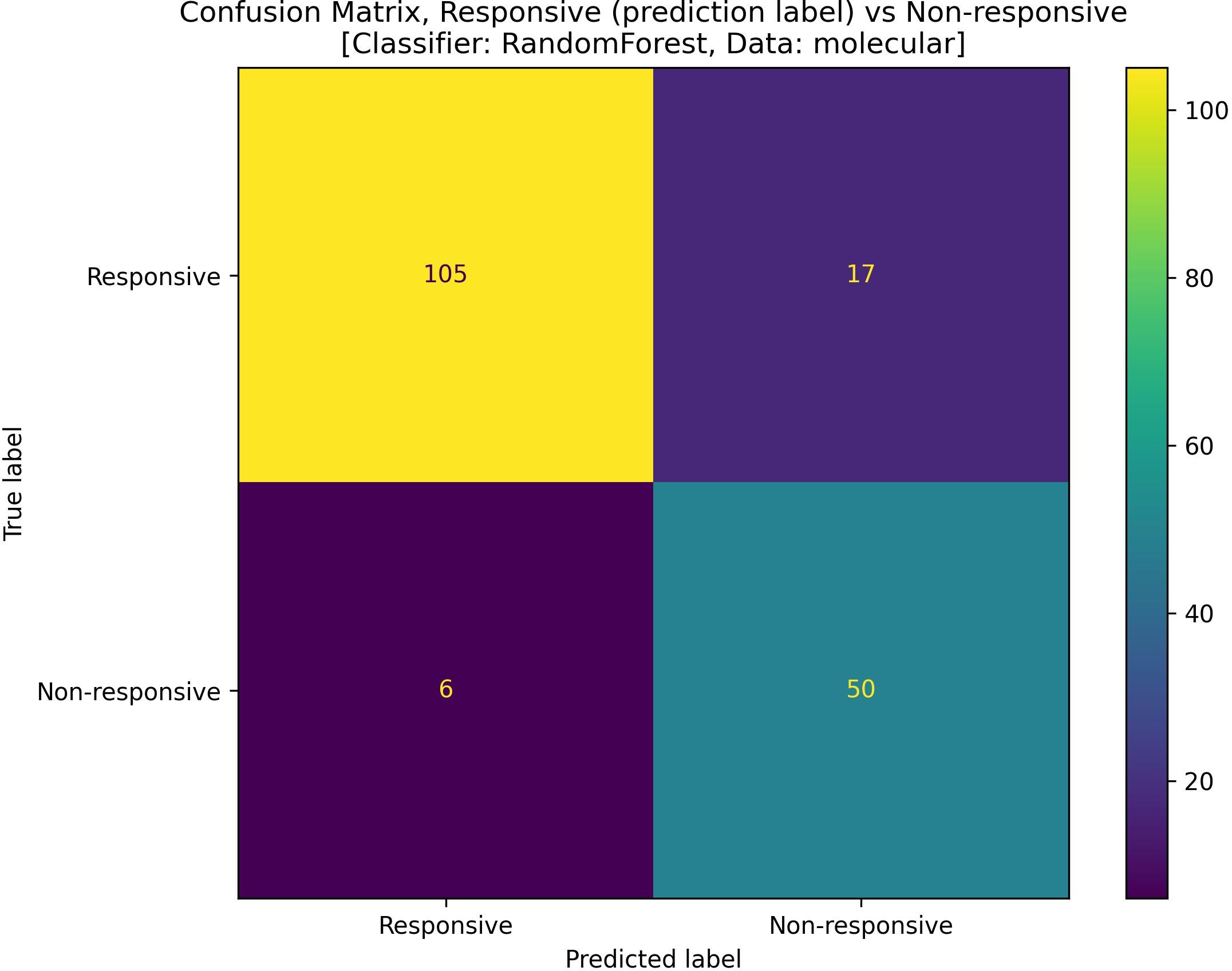


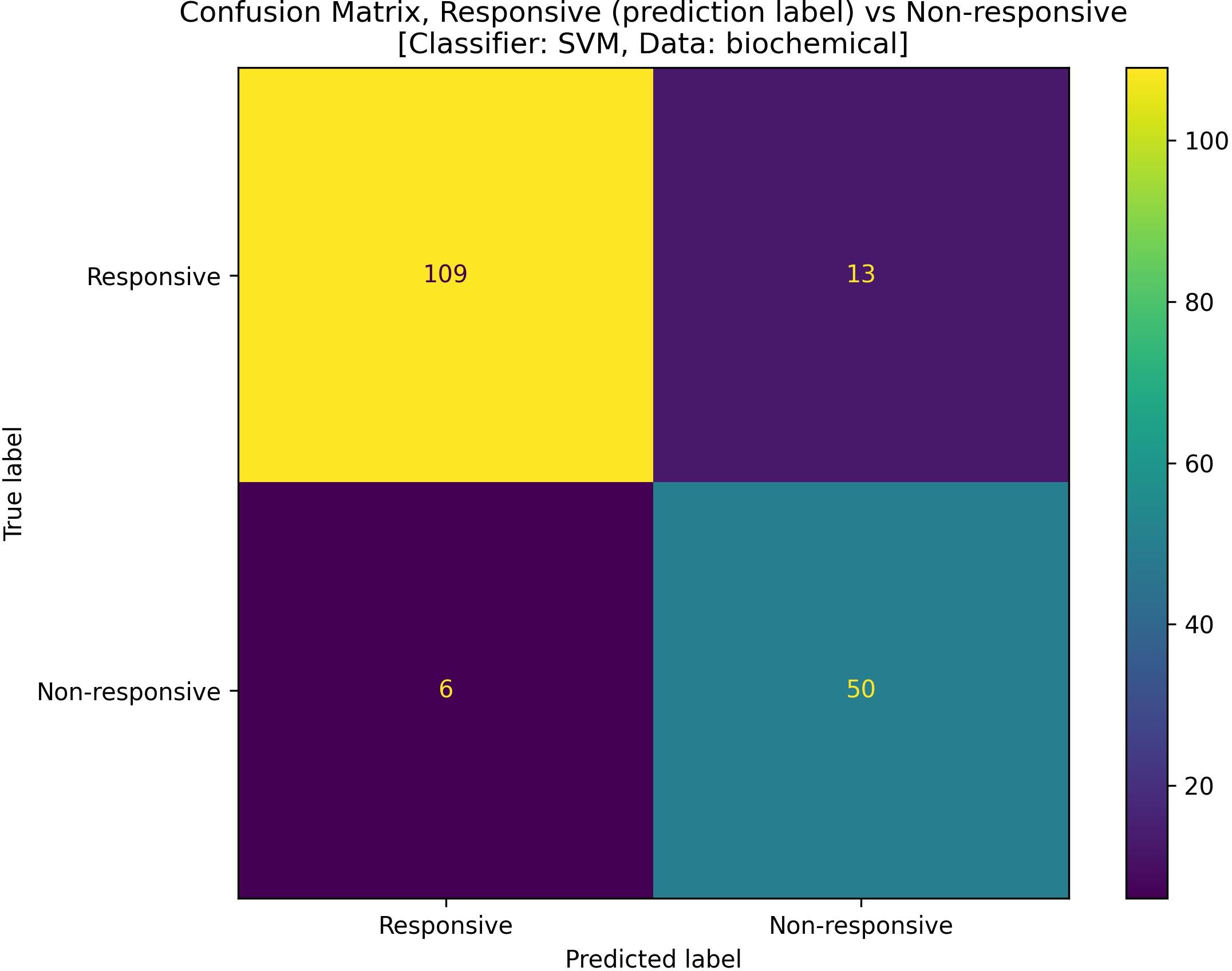


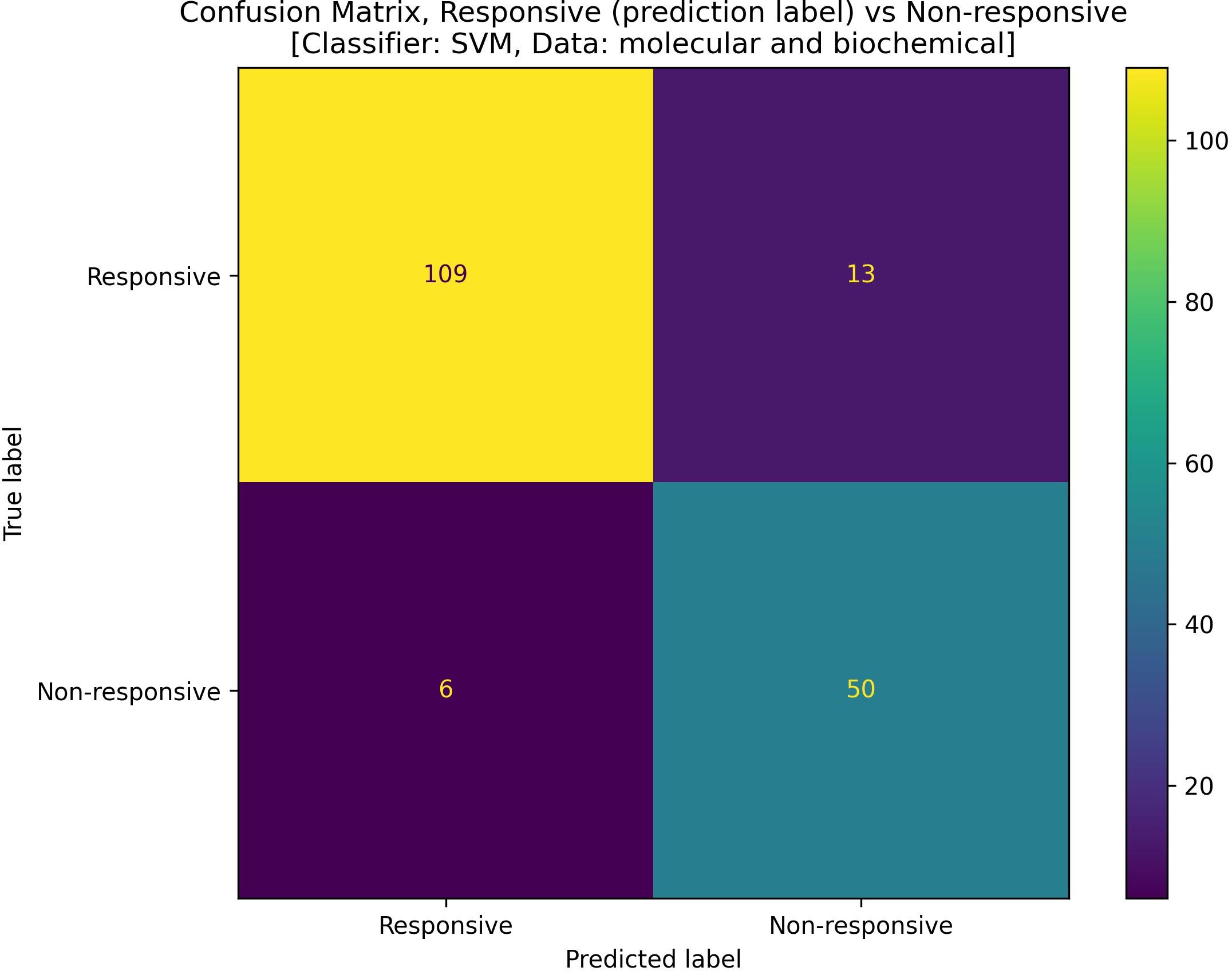


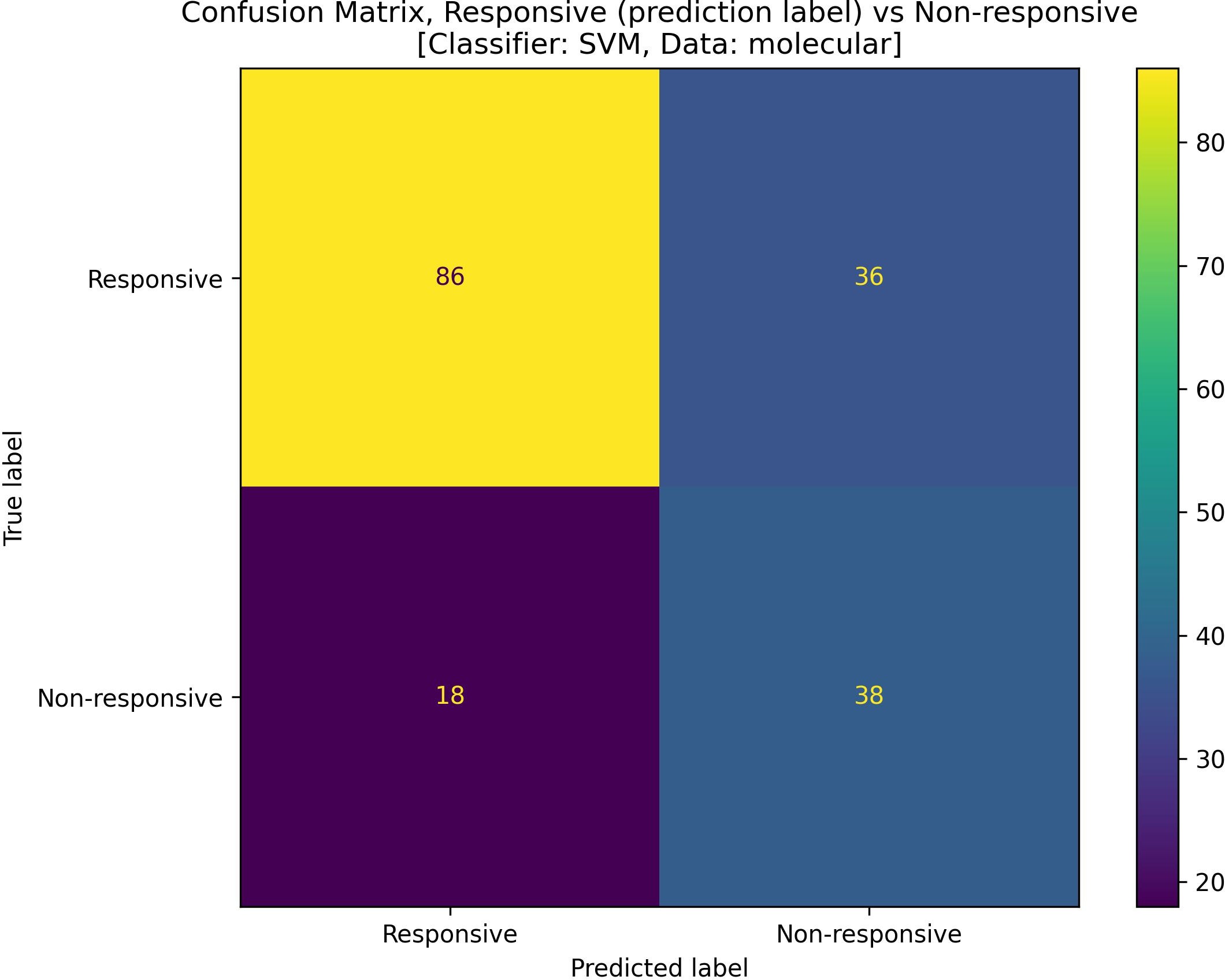


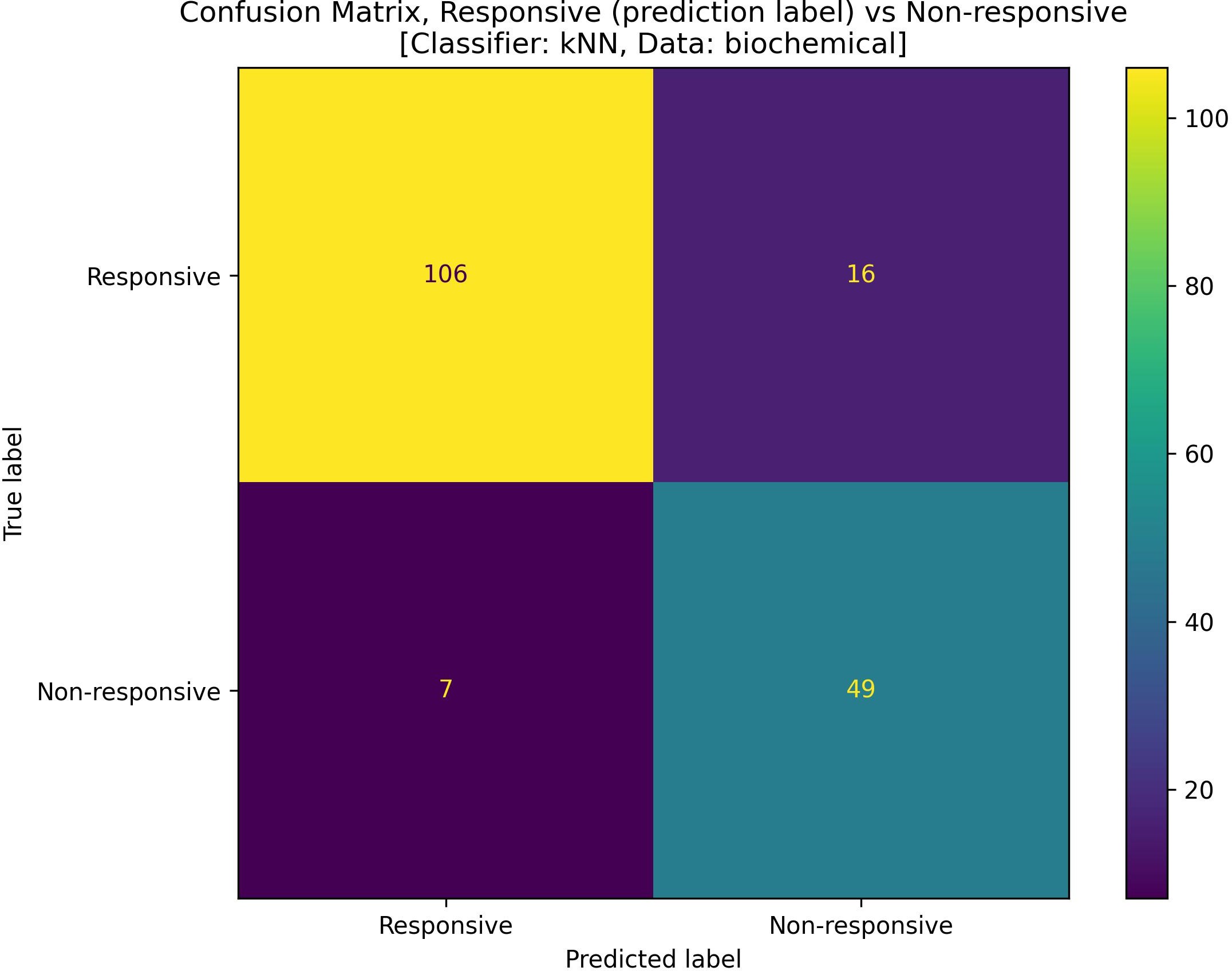


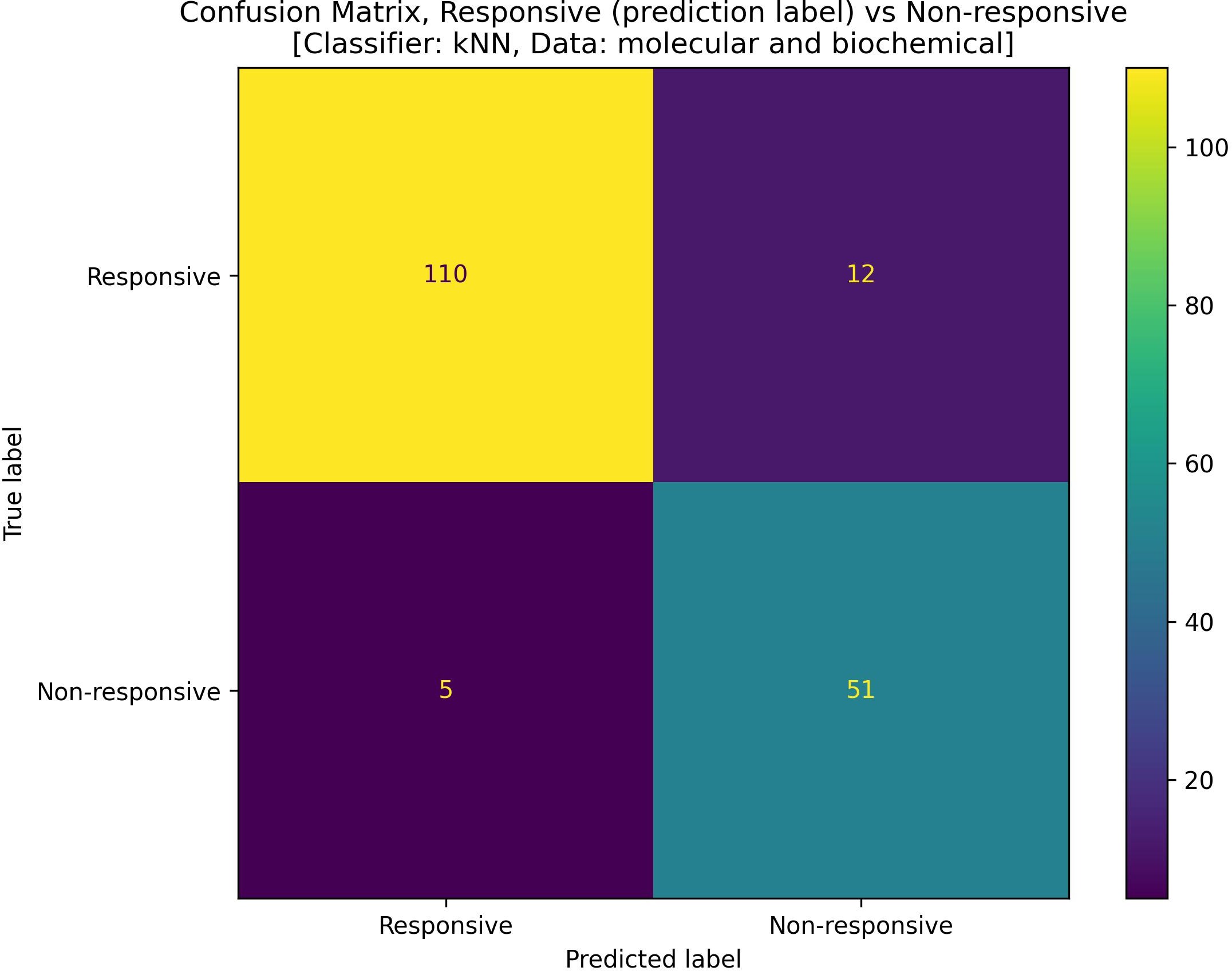


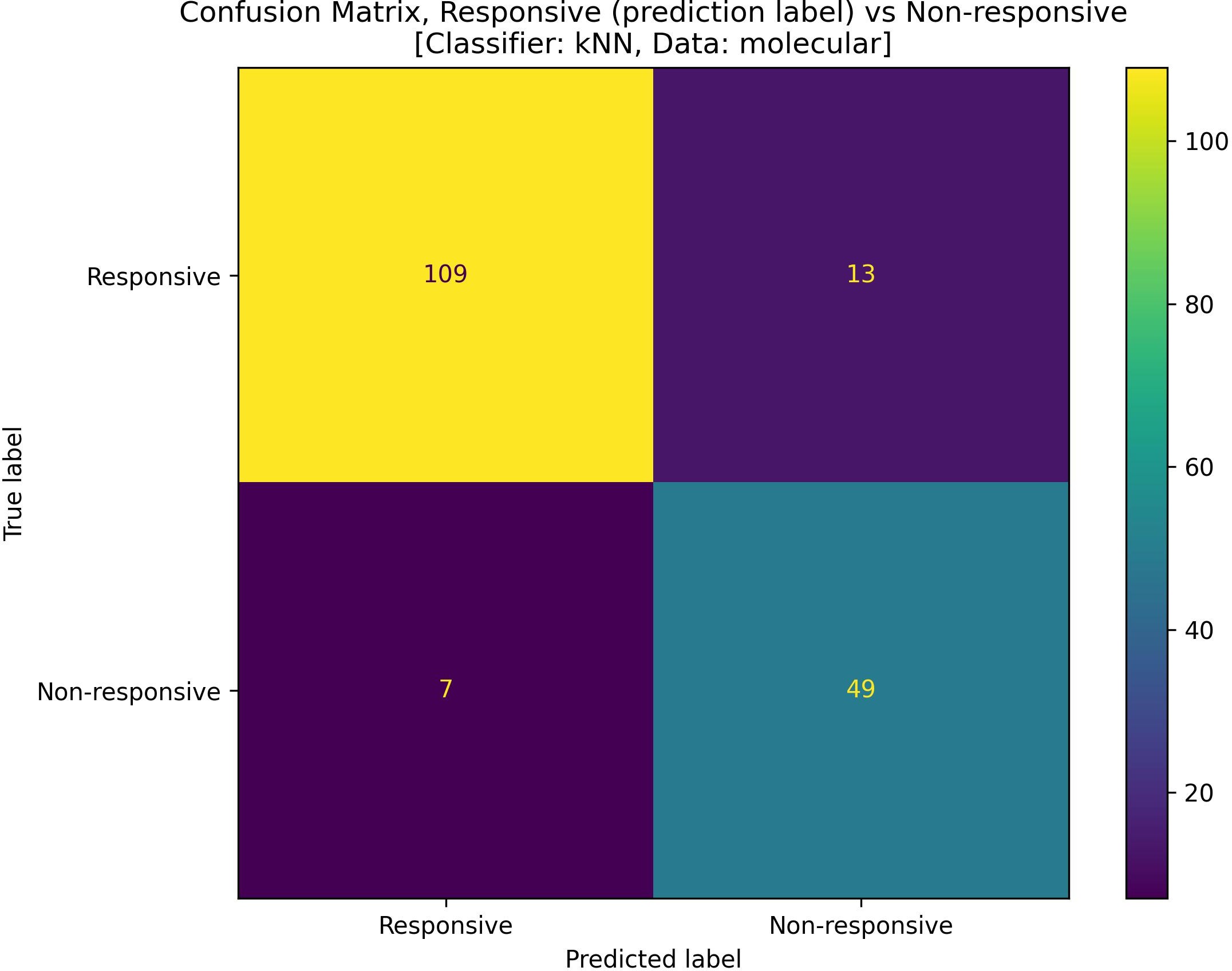


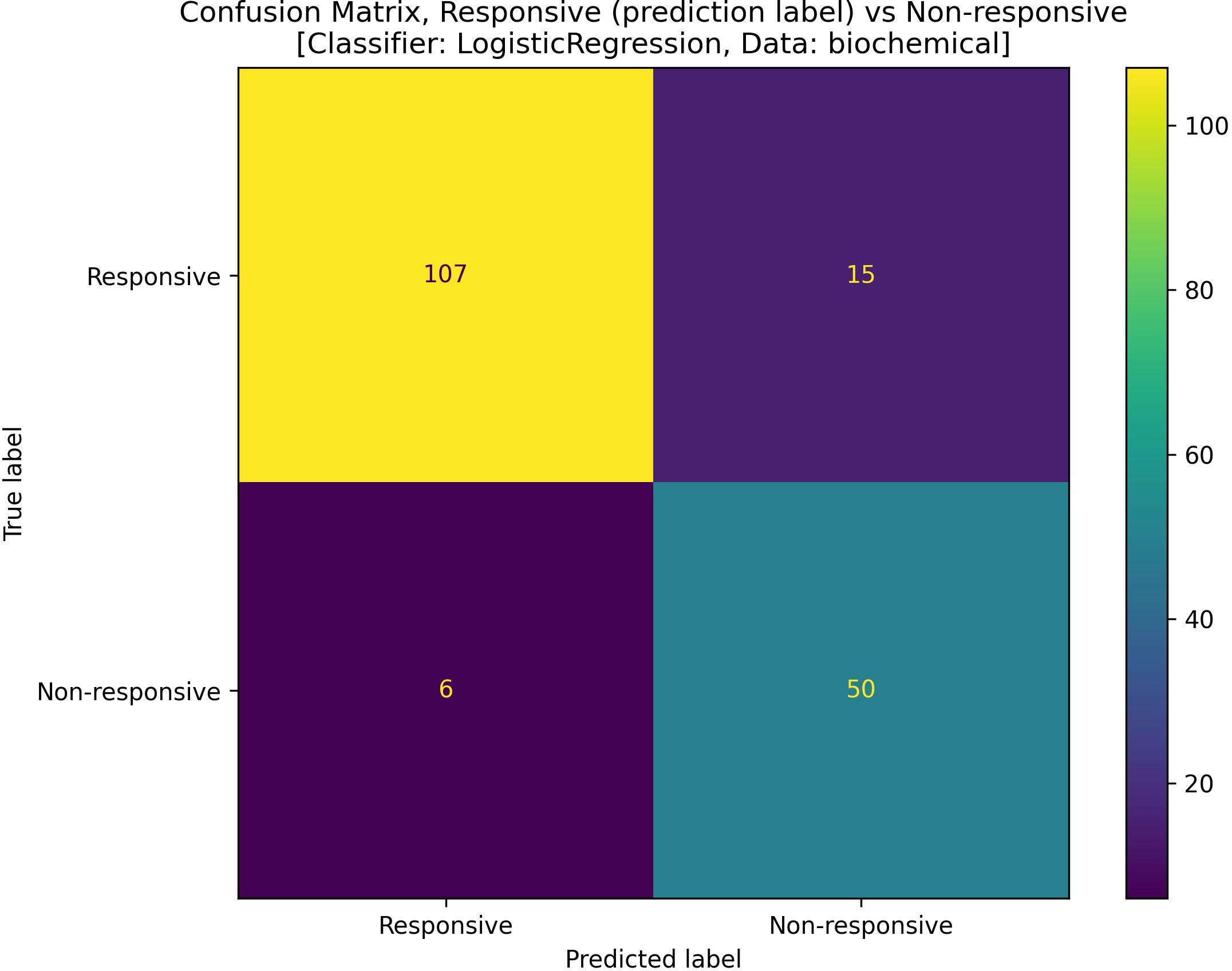


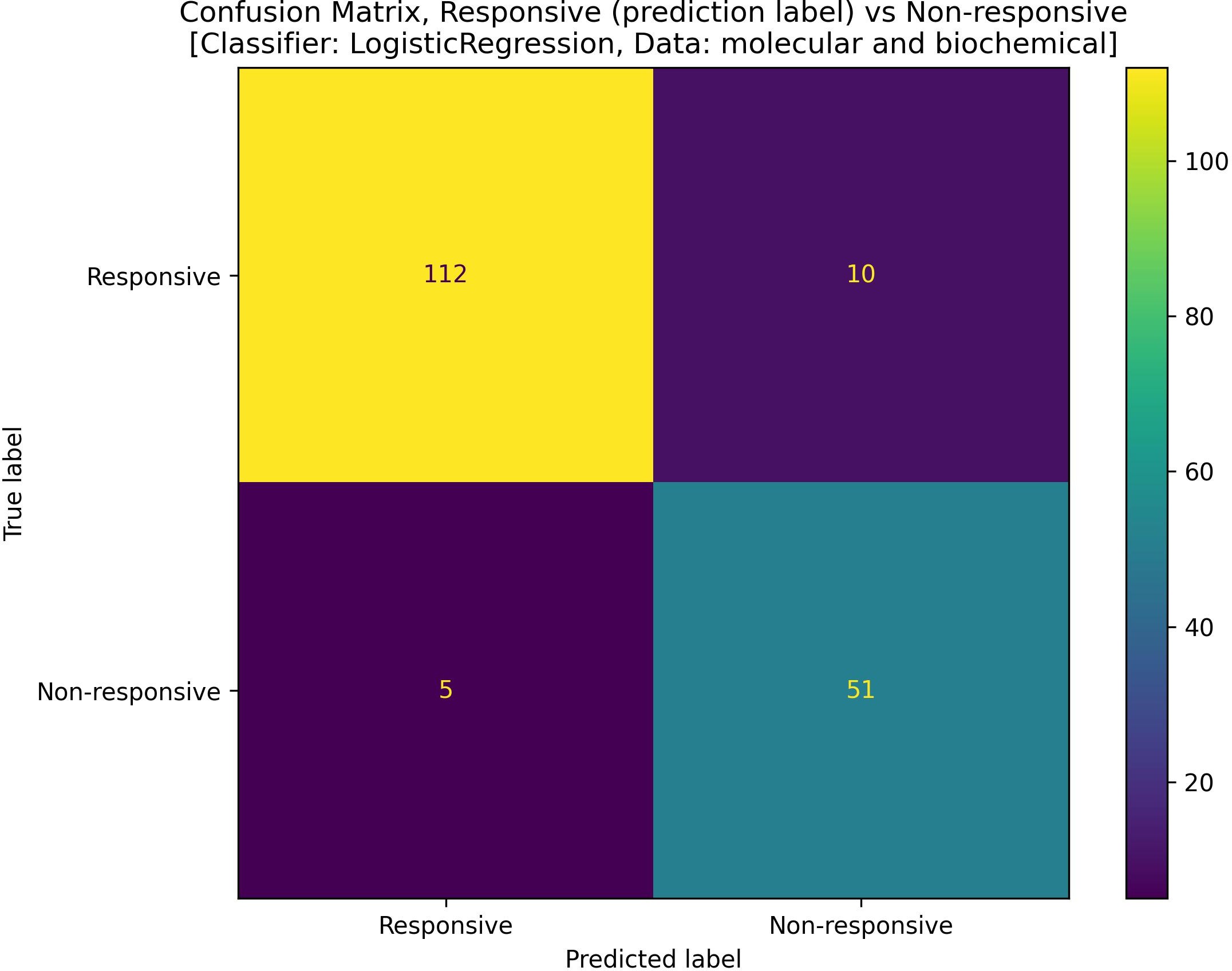


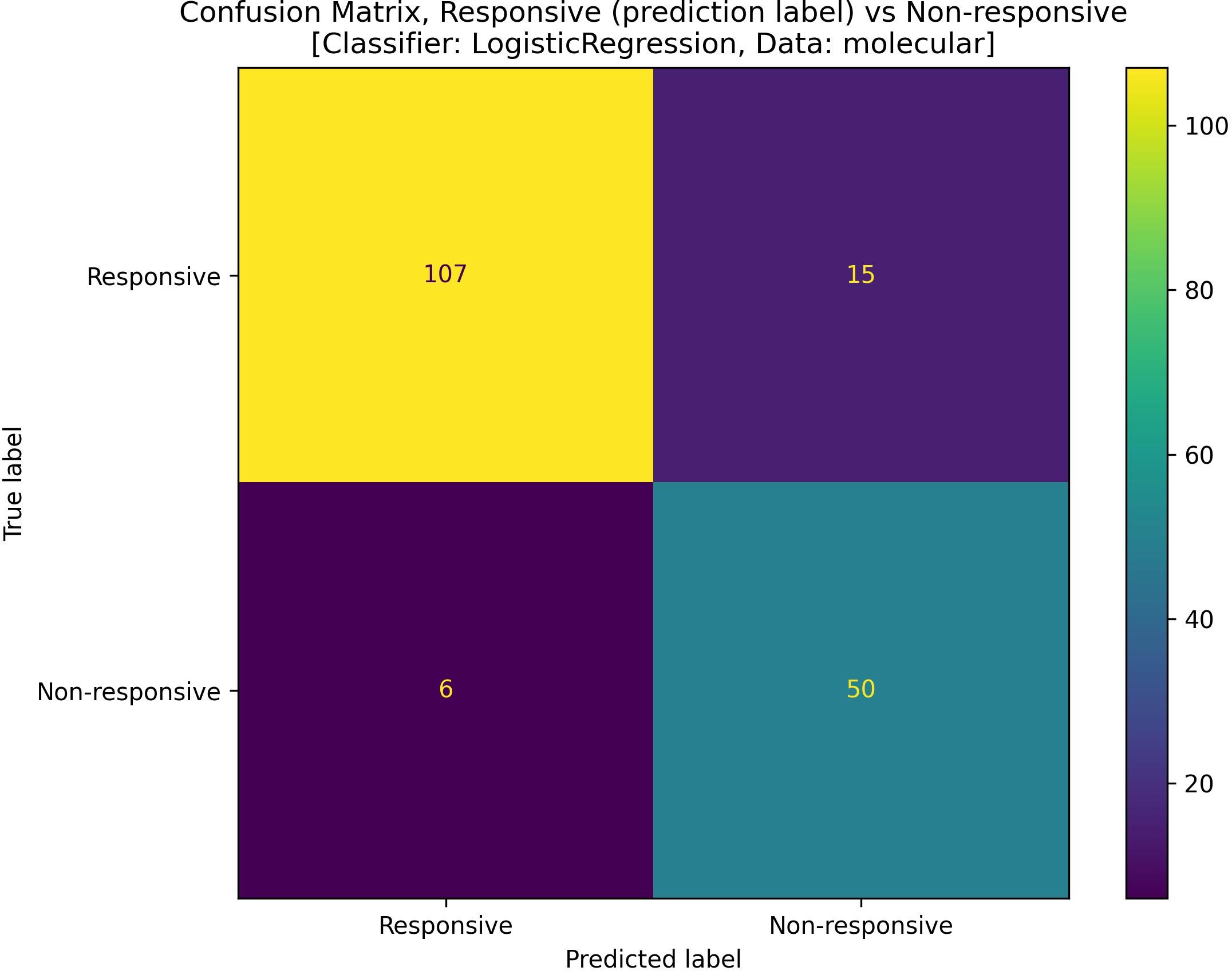


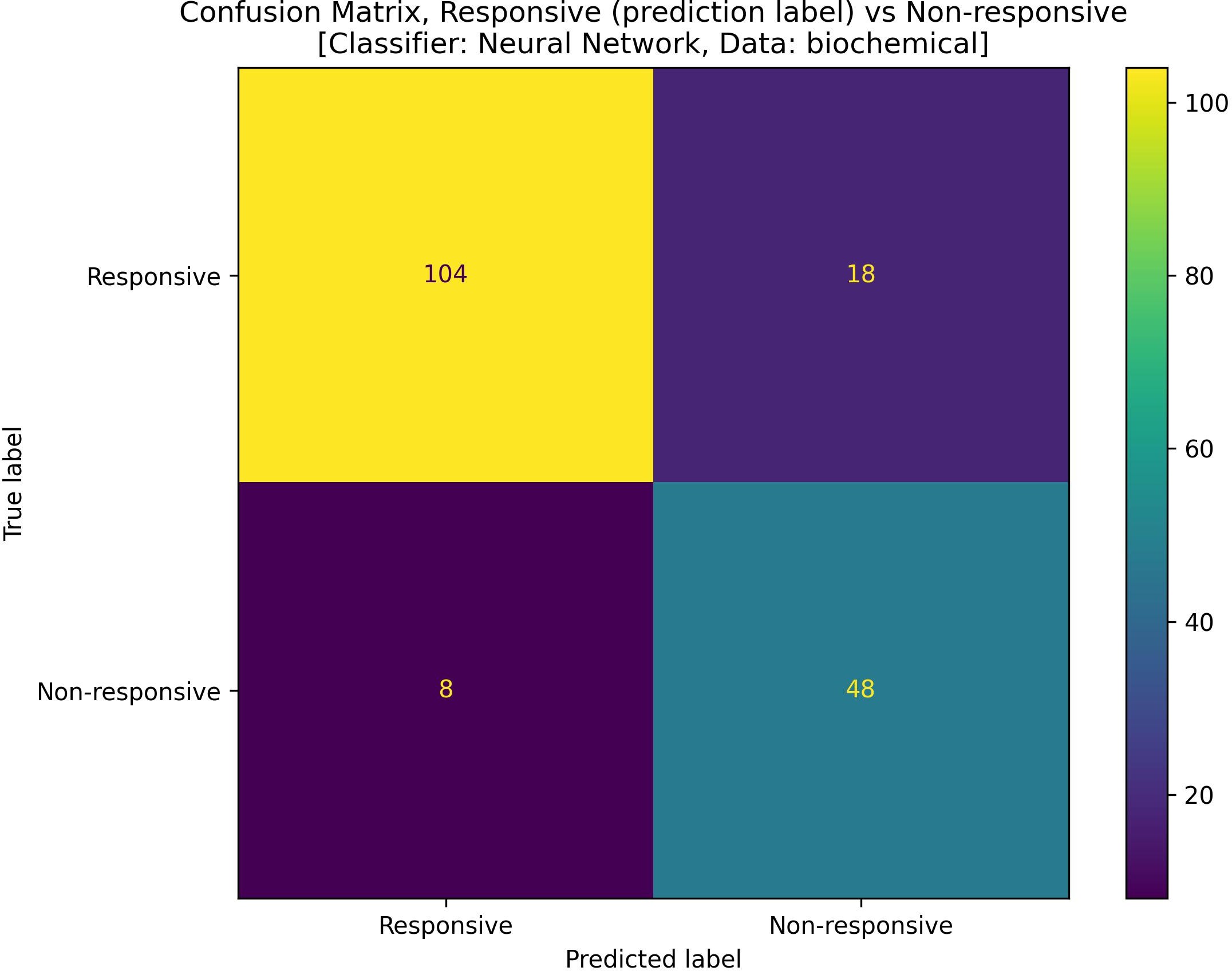


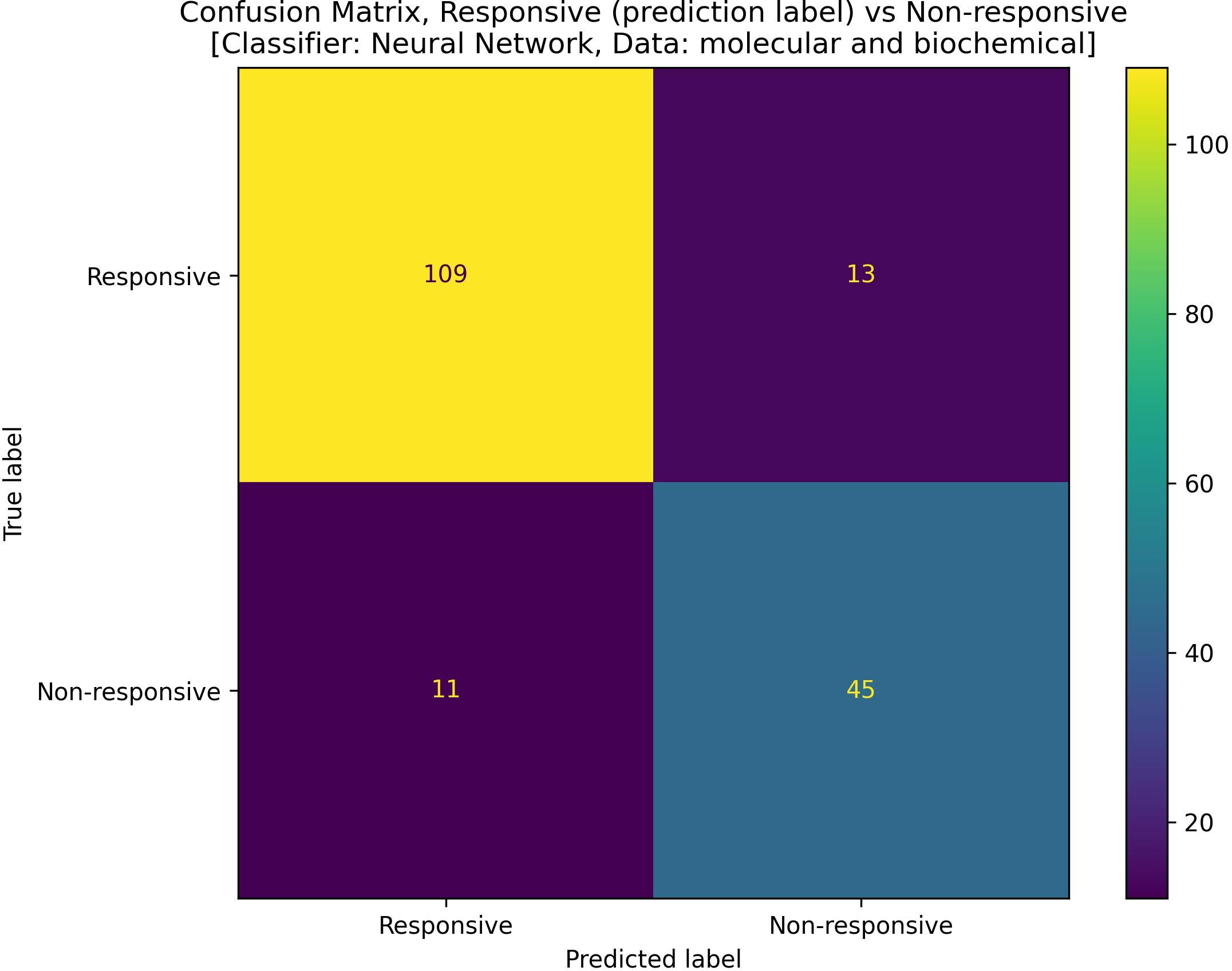


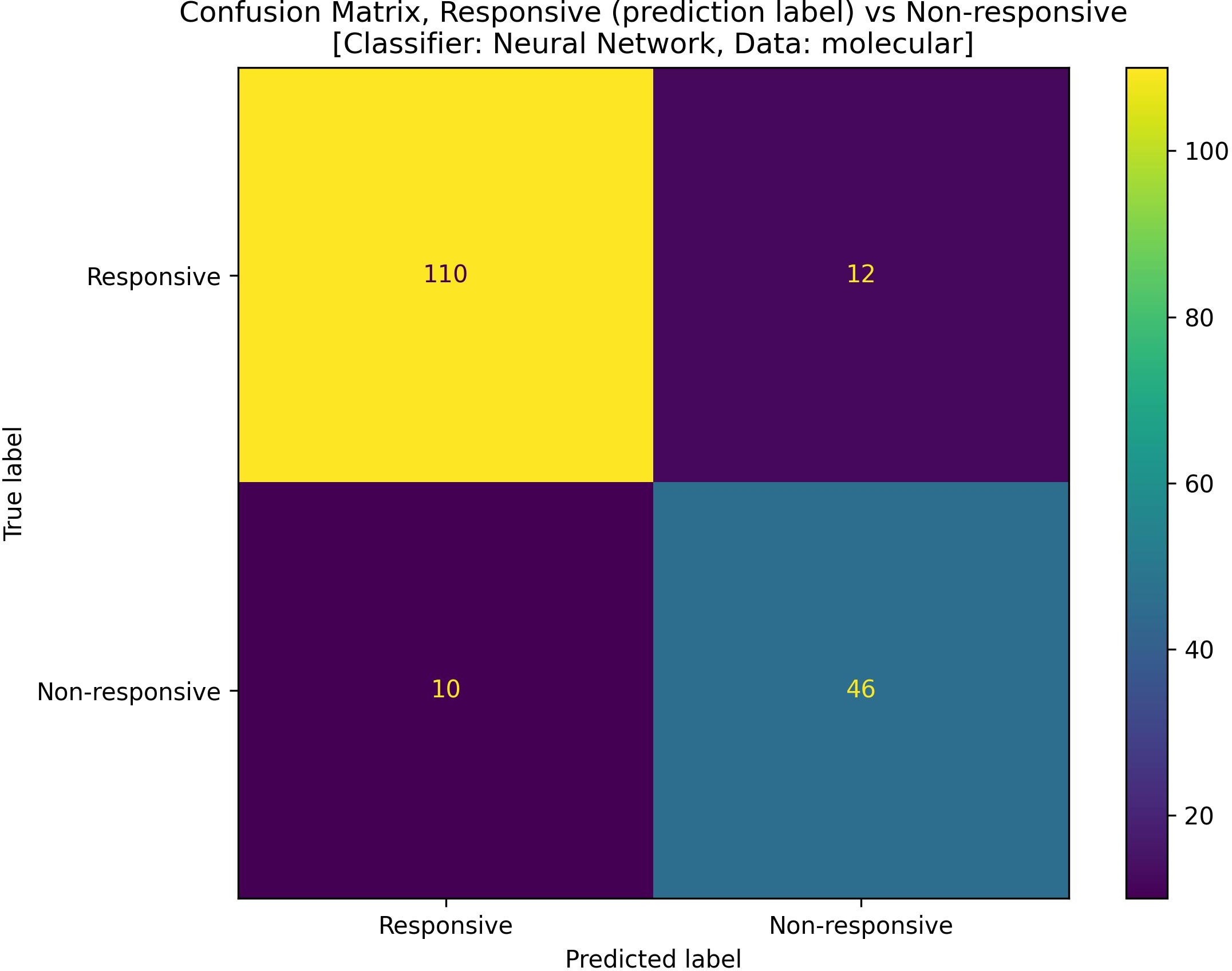


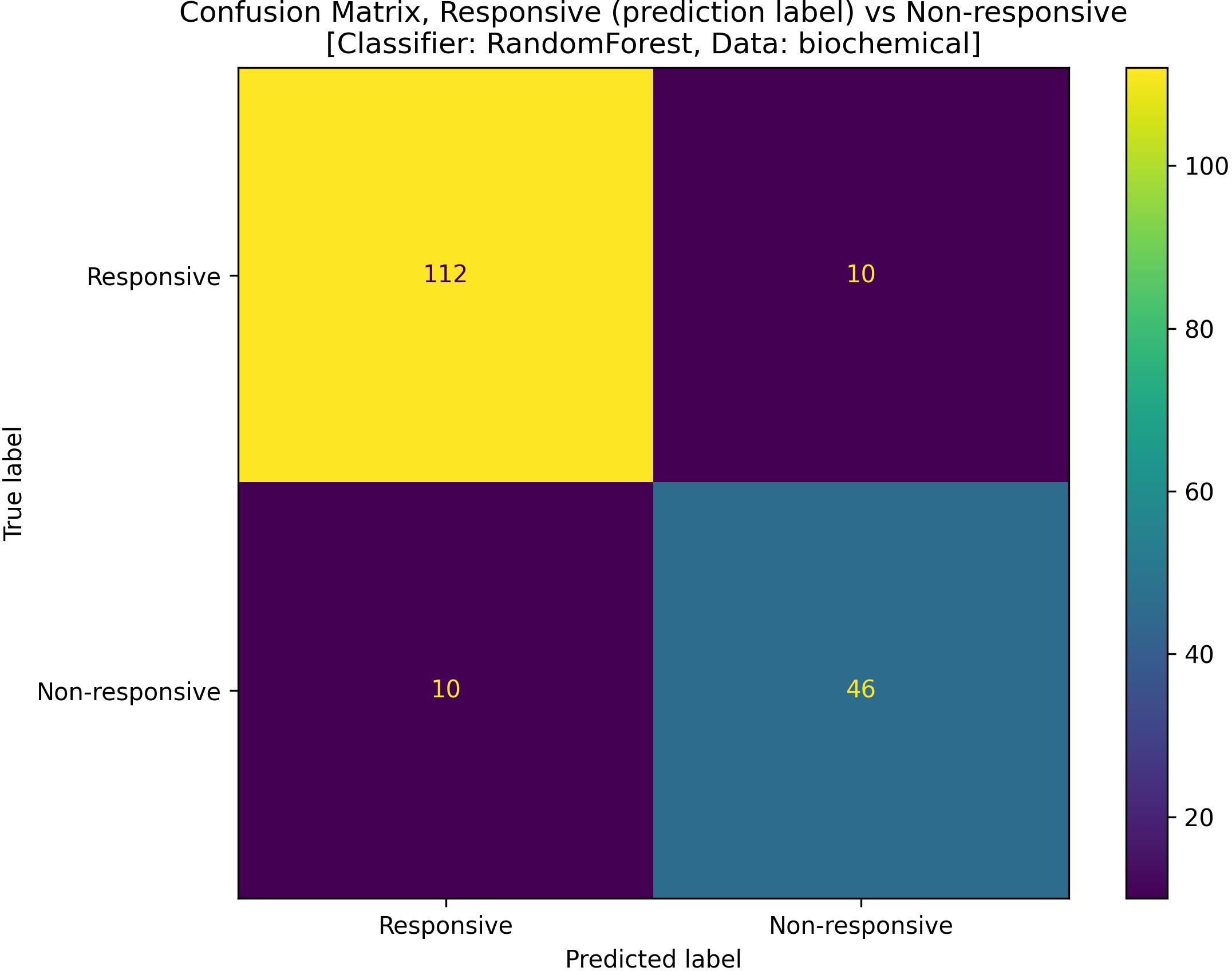


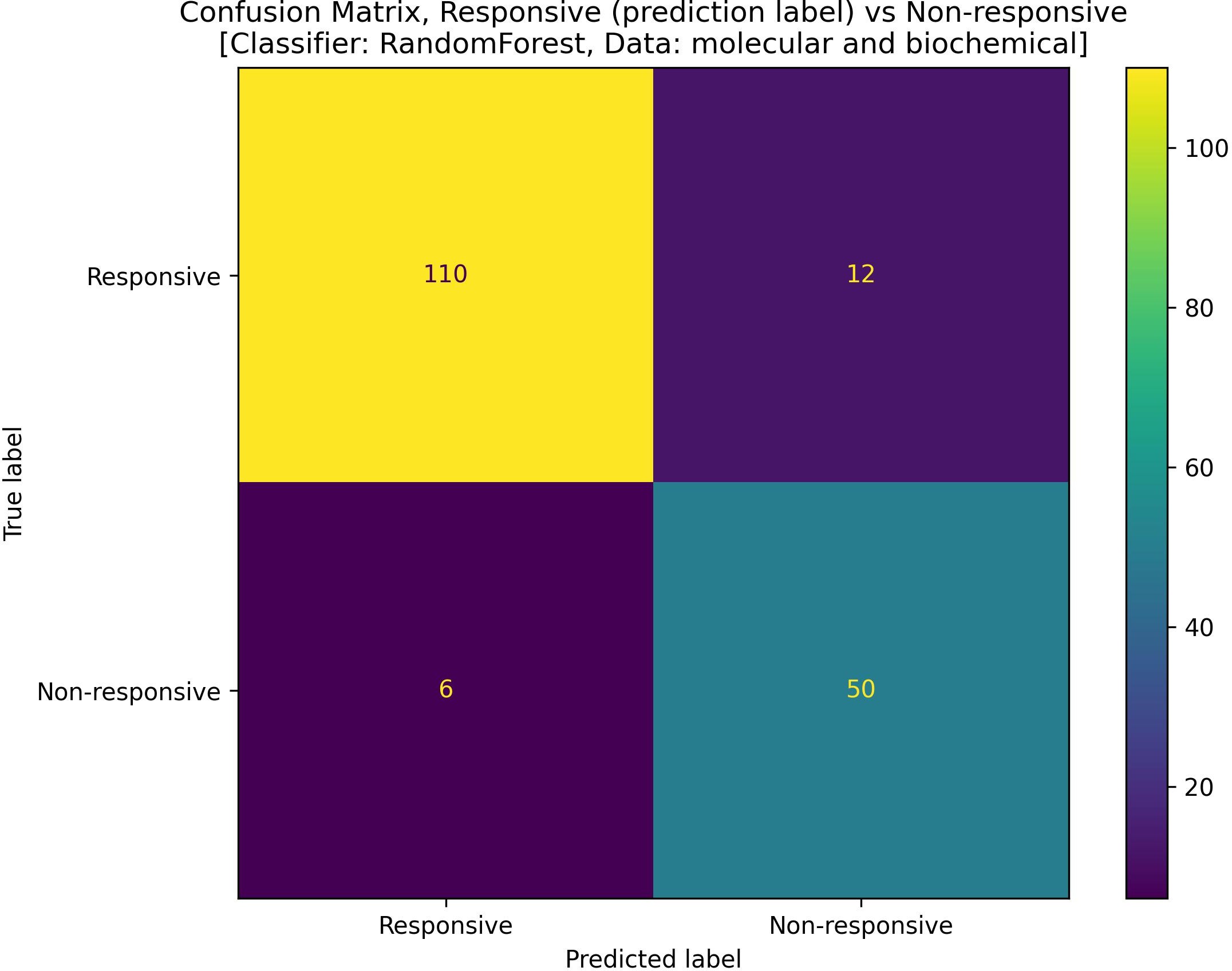


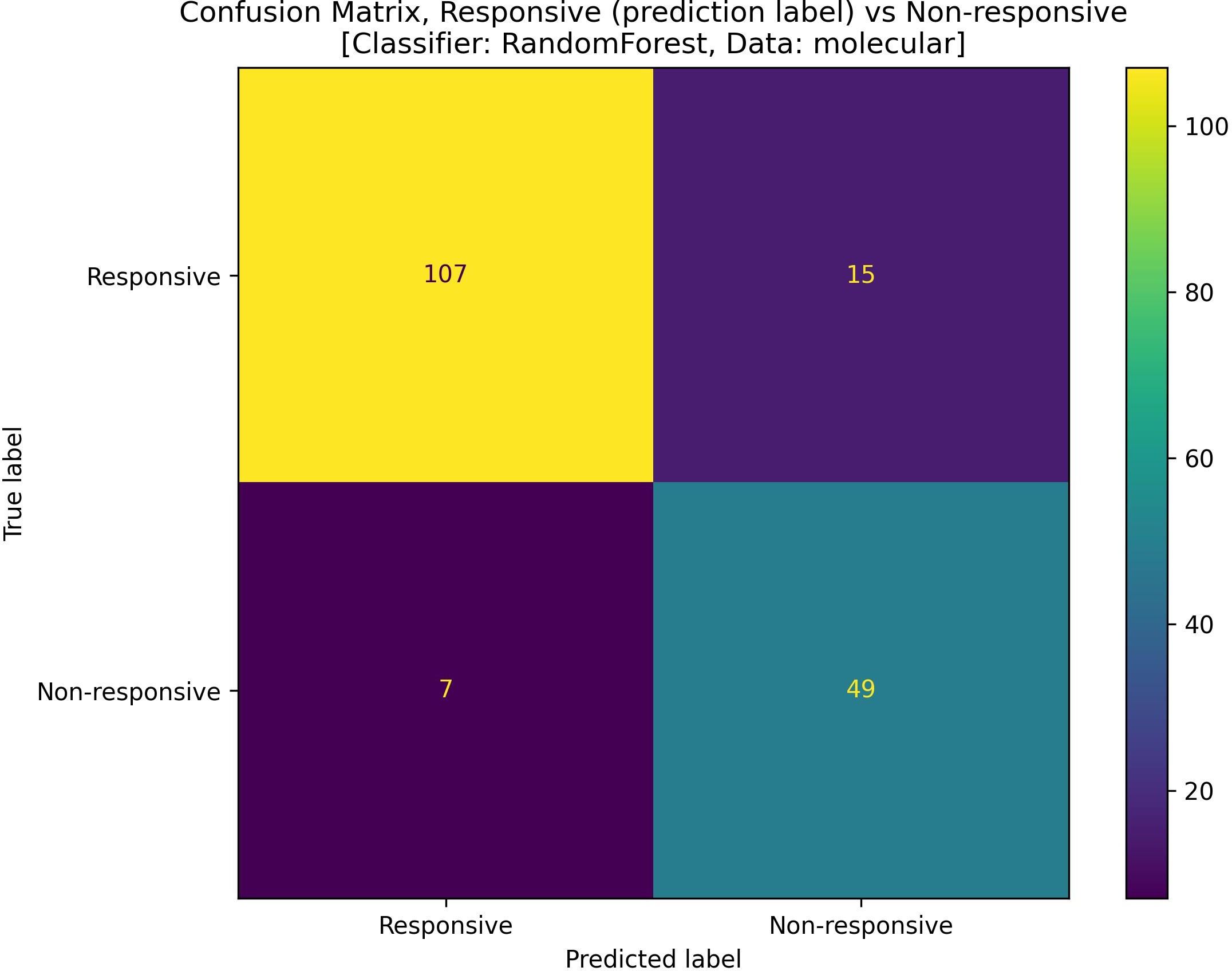


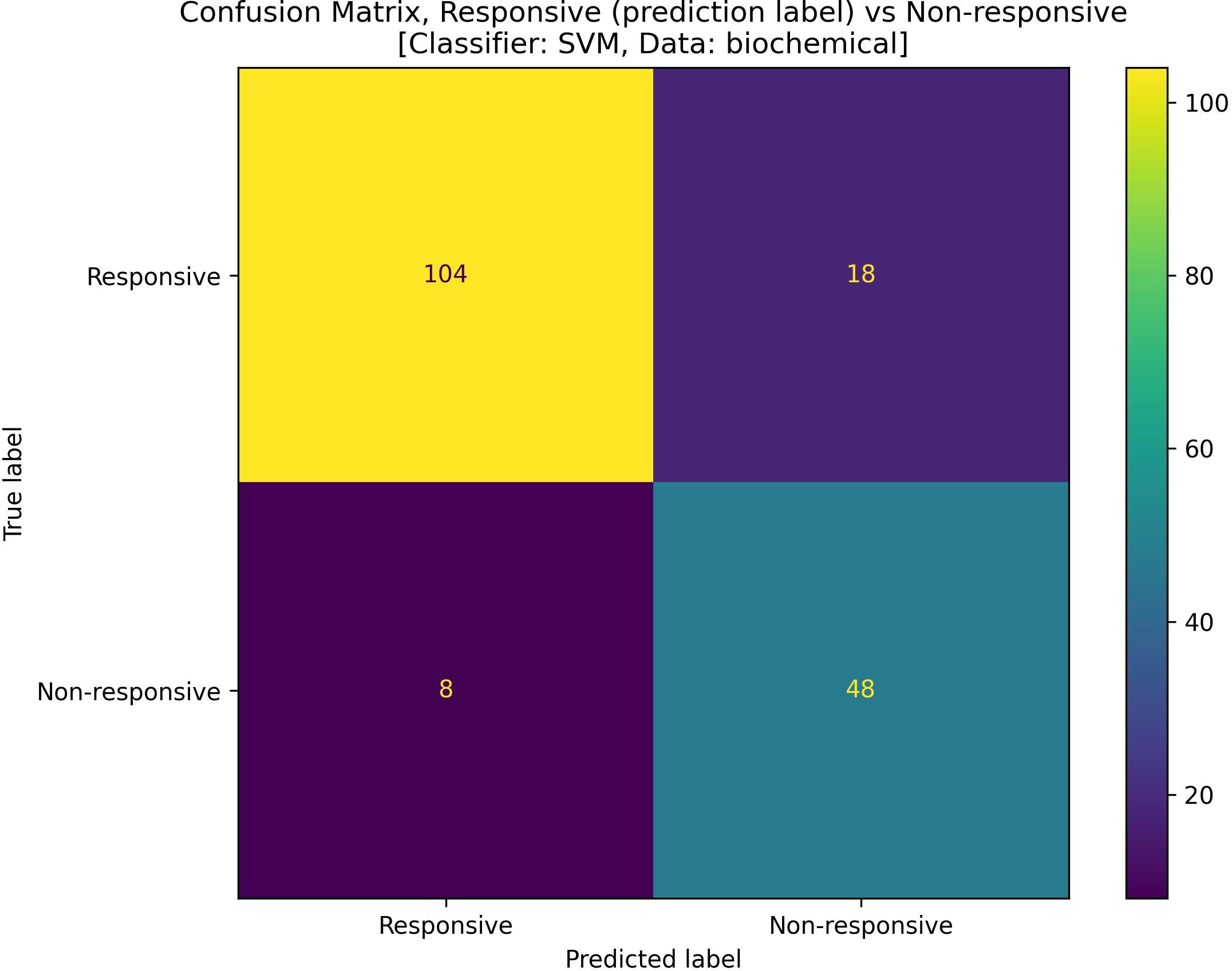


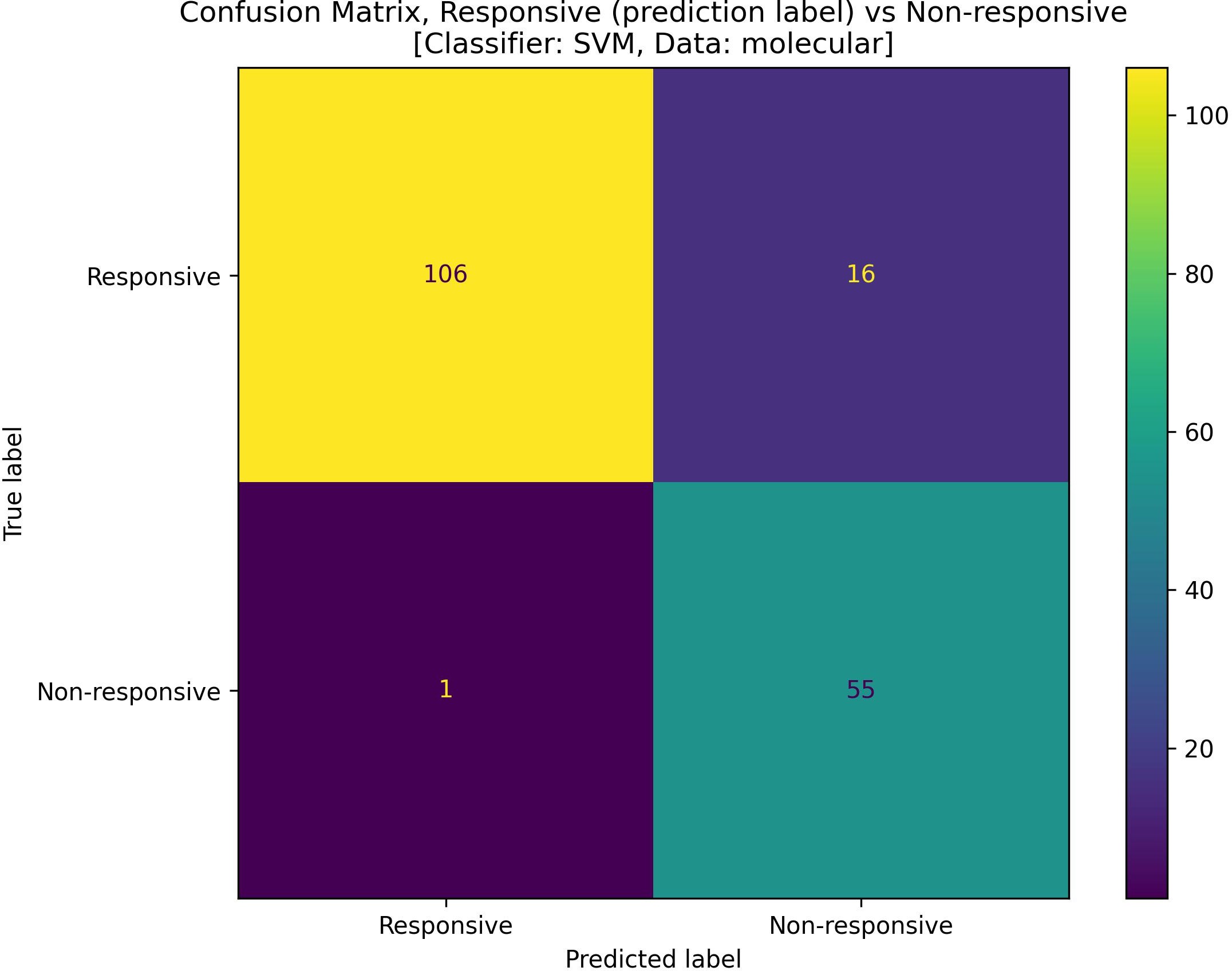


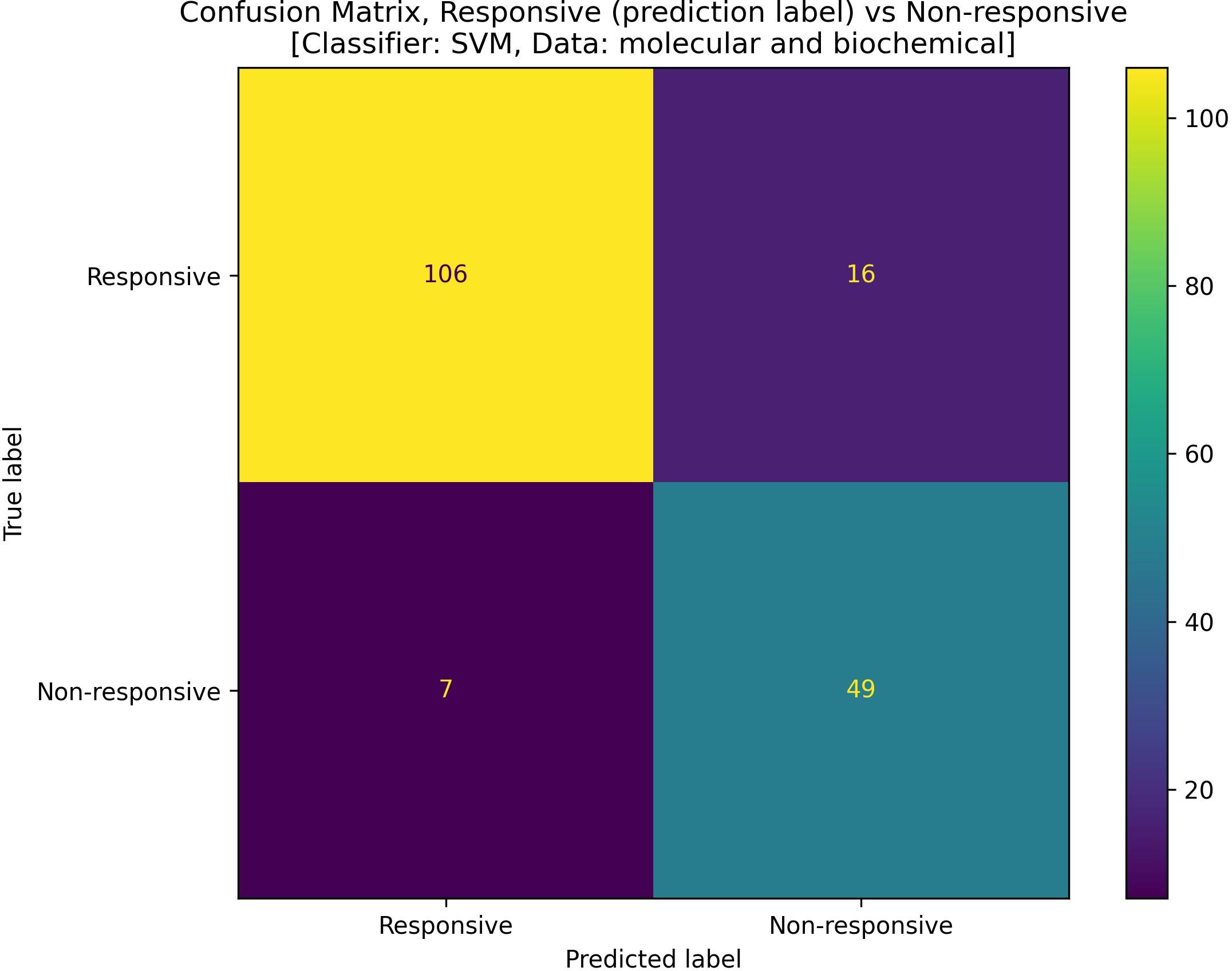


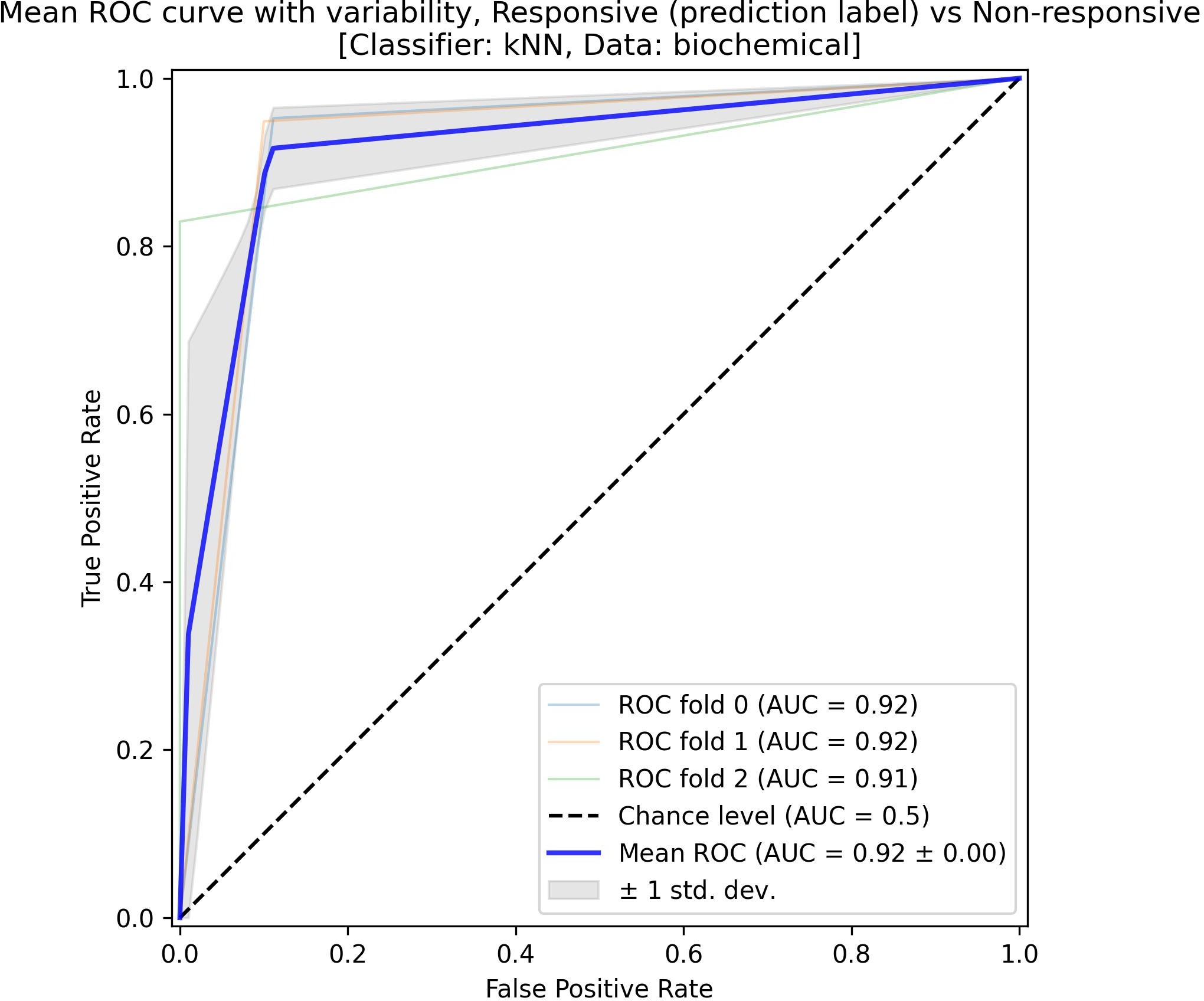
**Figure S6. Roc Curves**


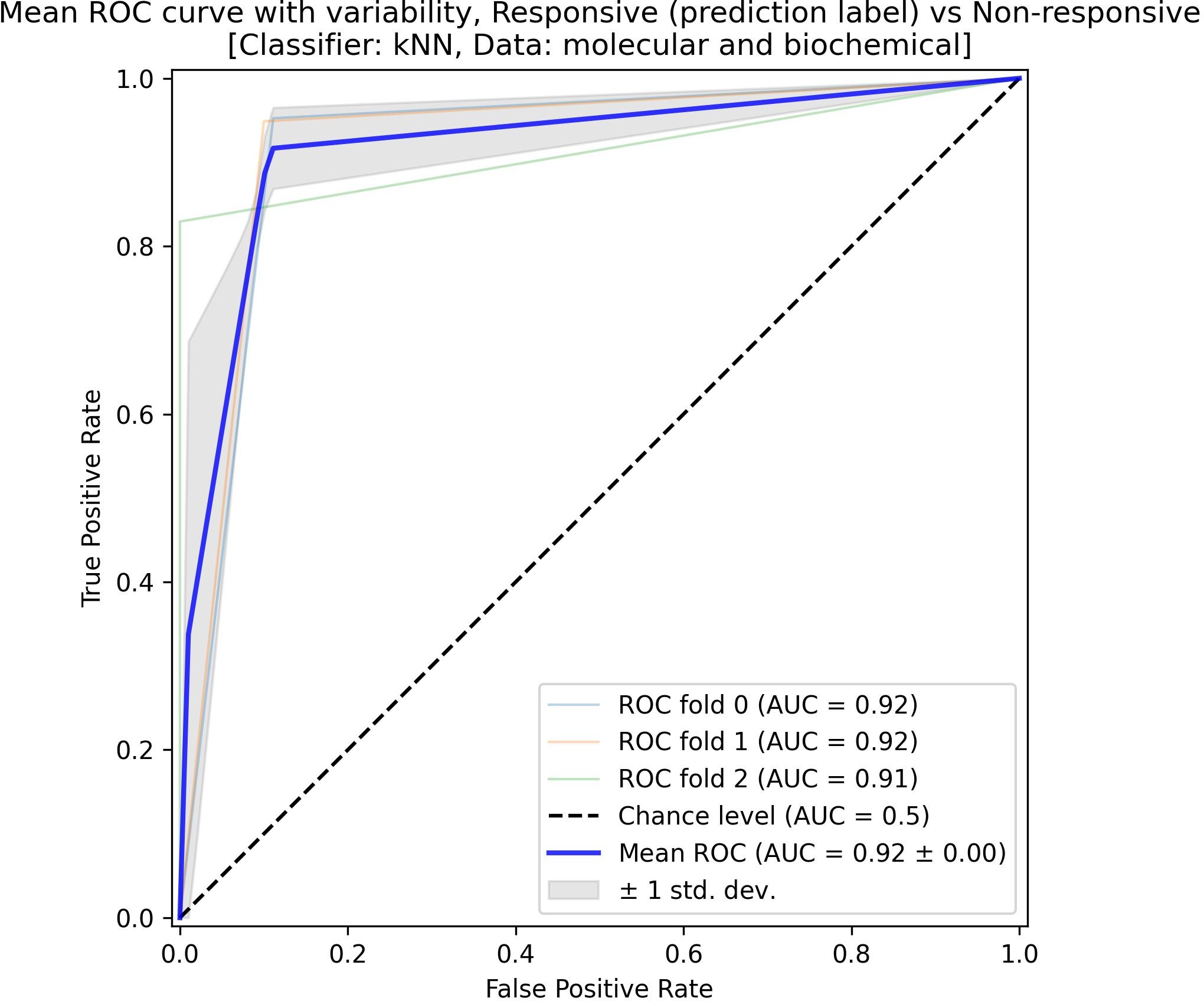


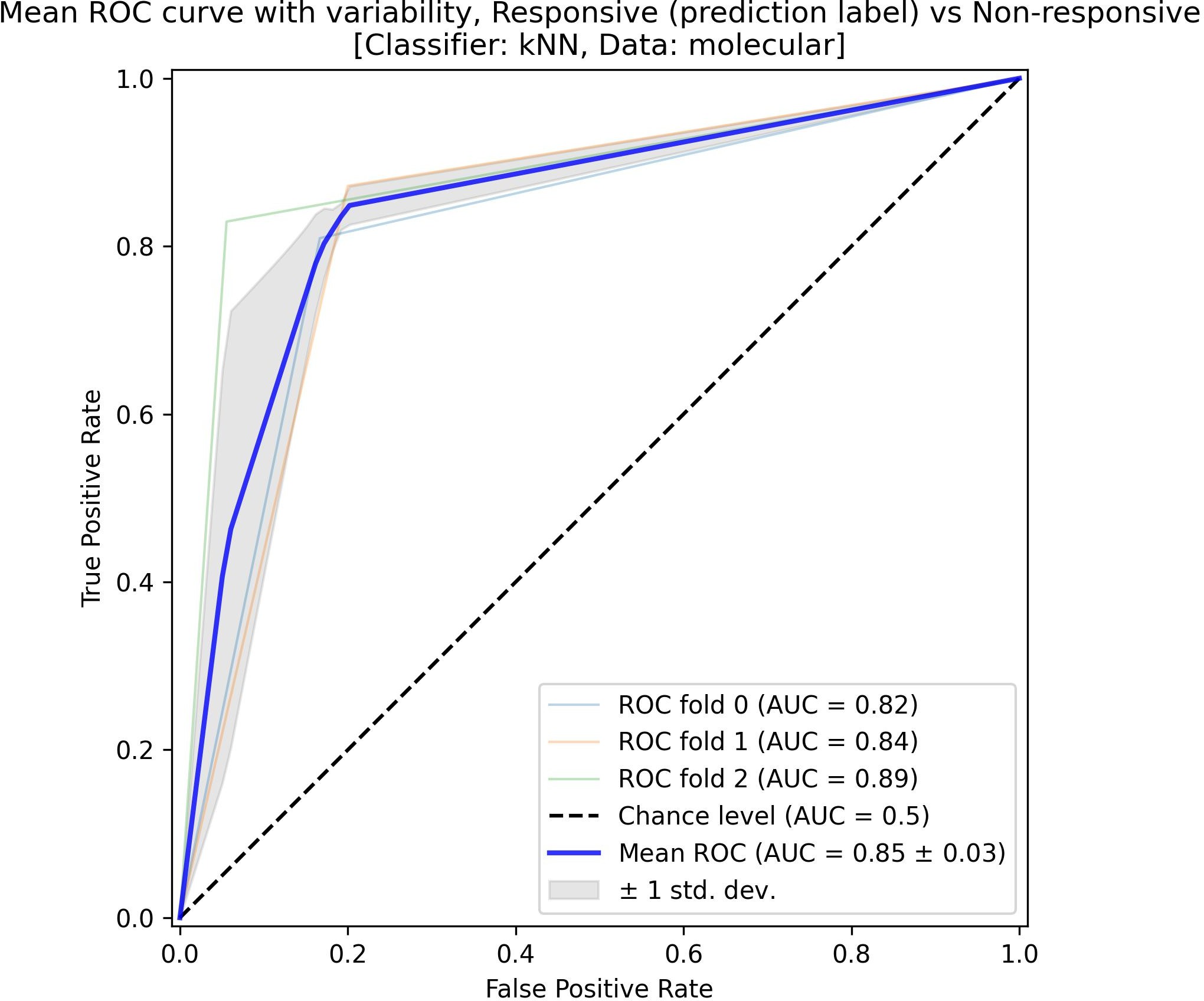


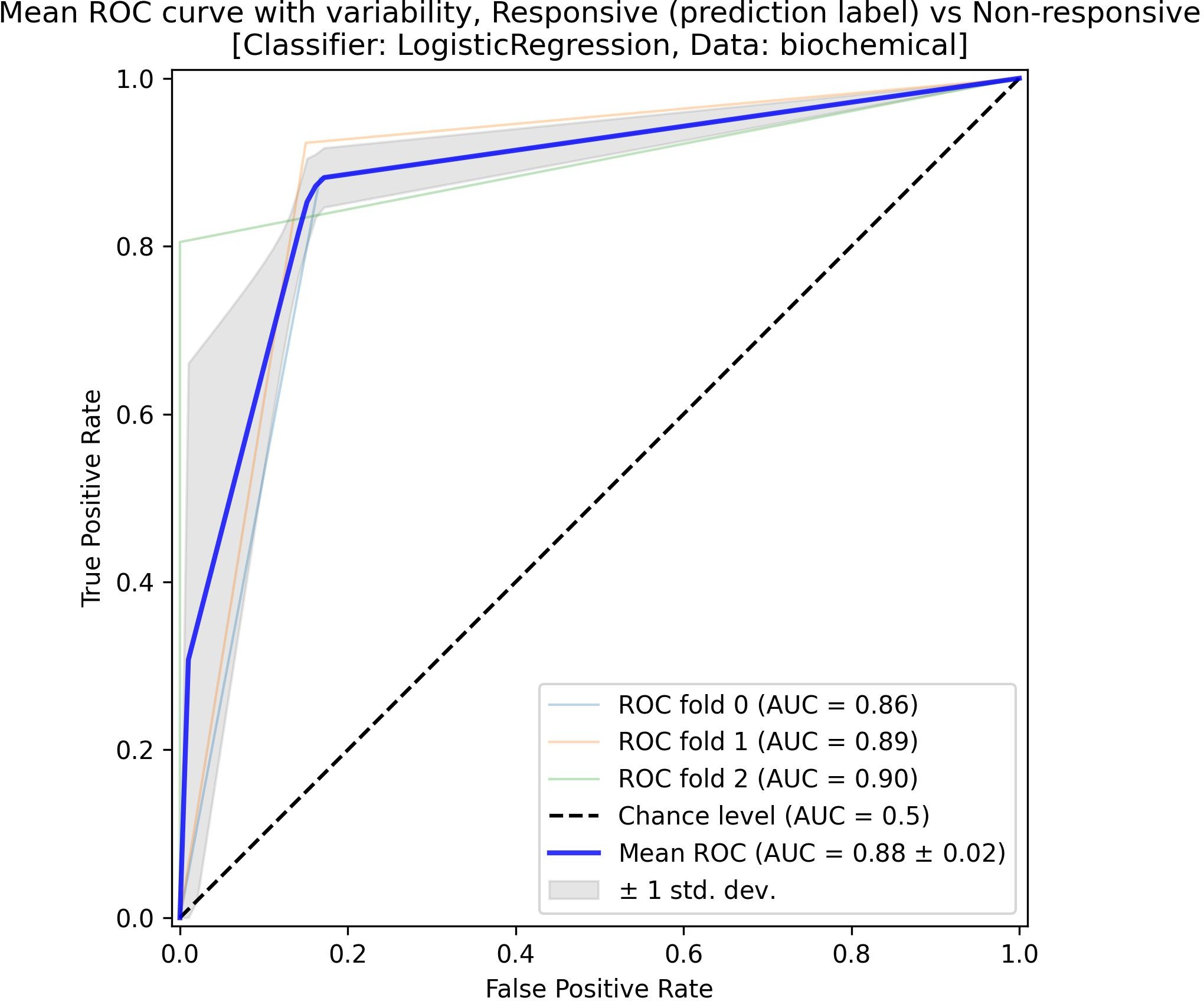


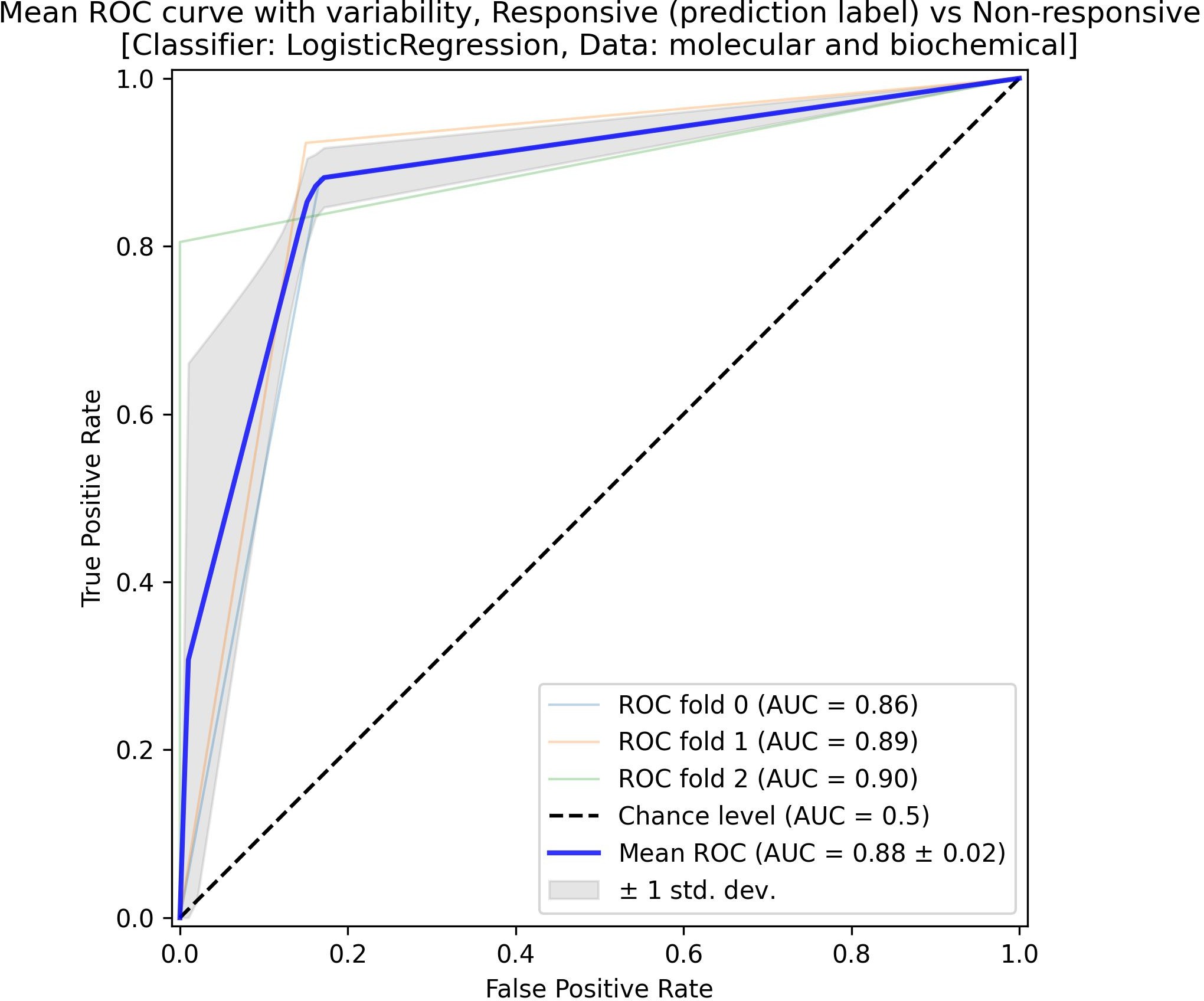


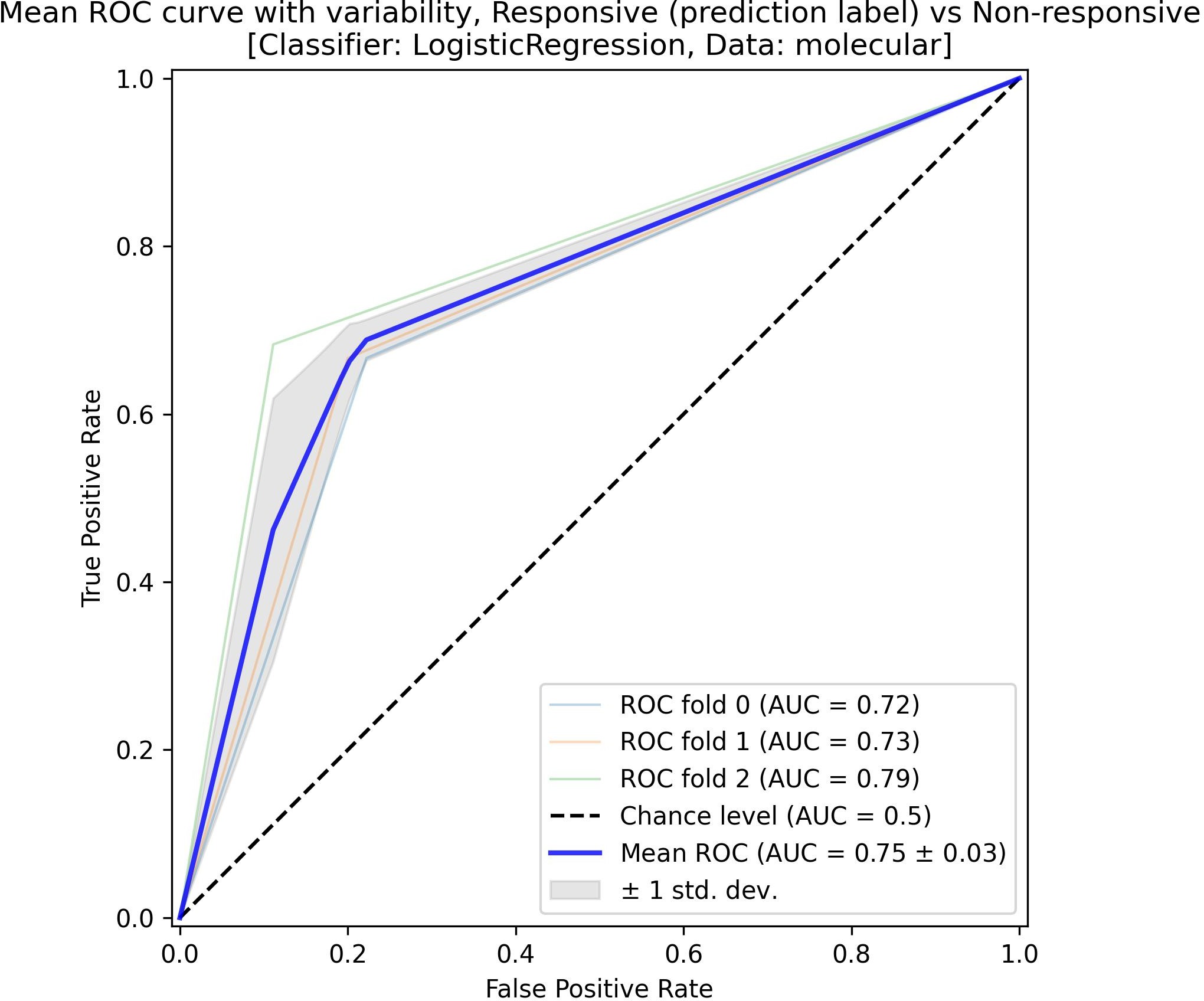


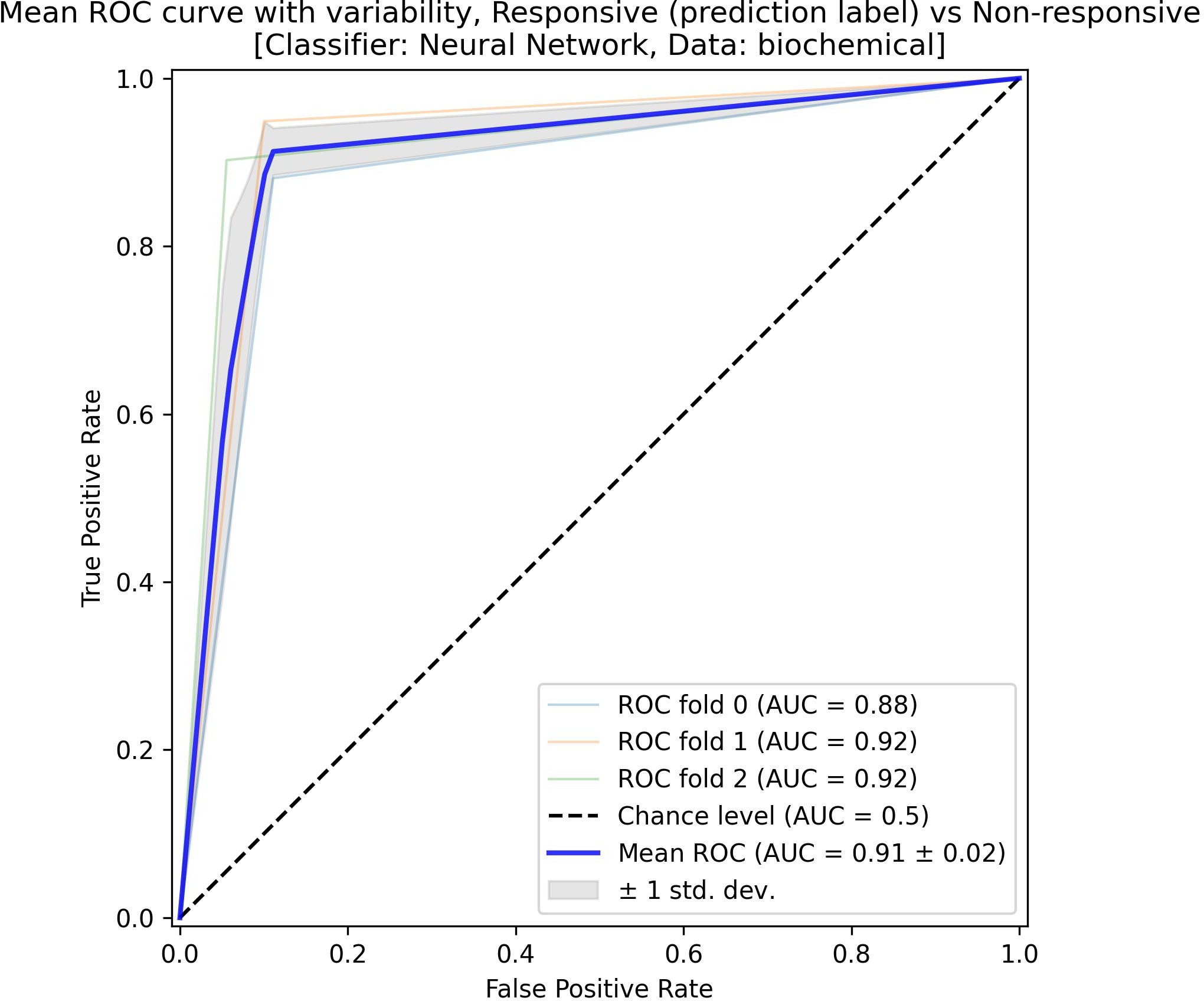


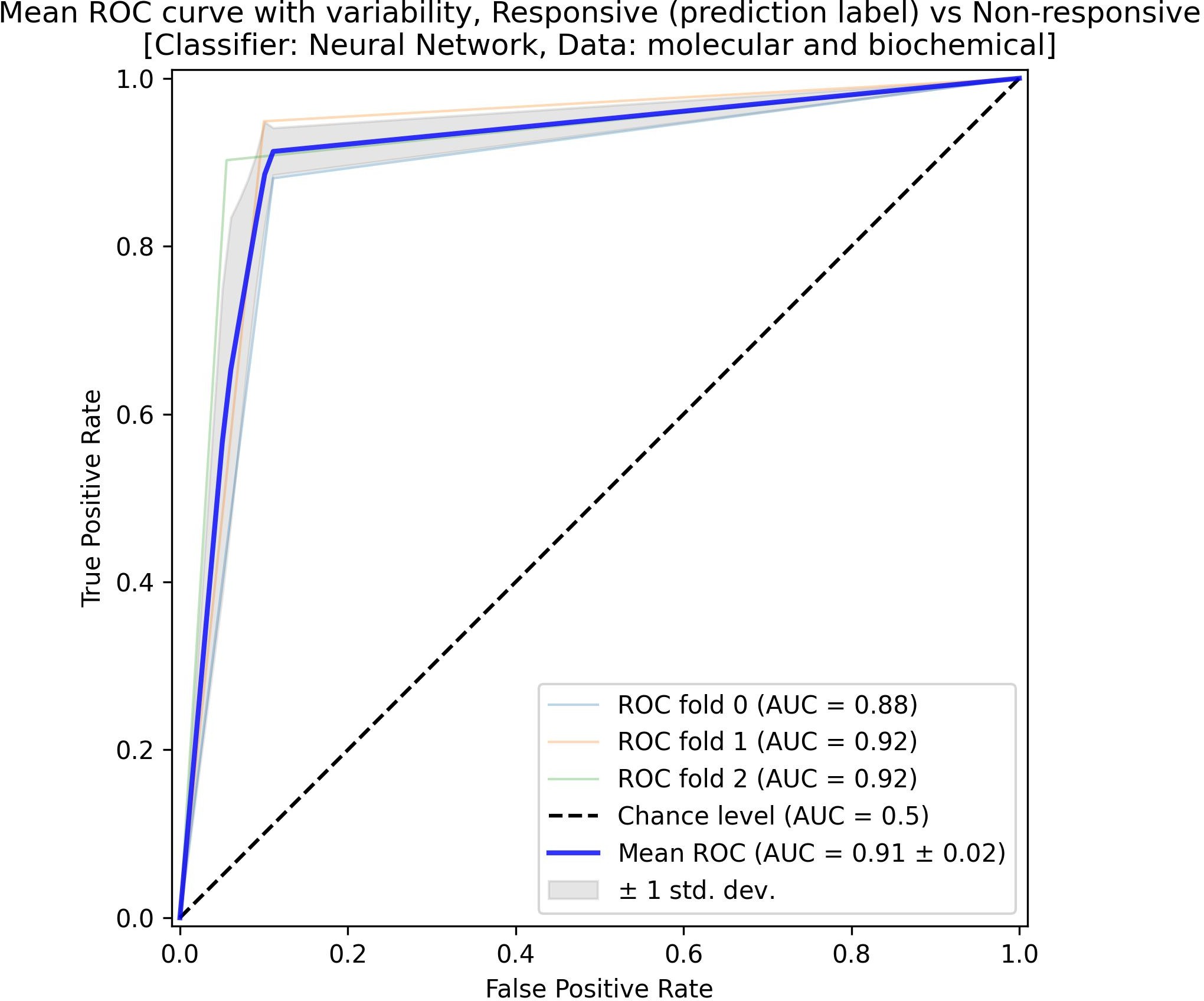


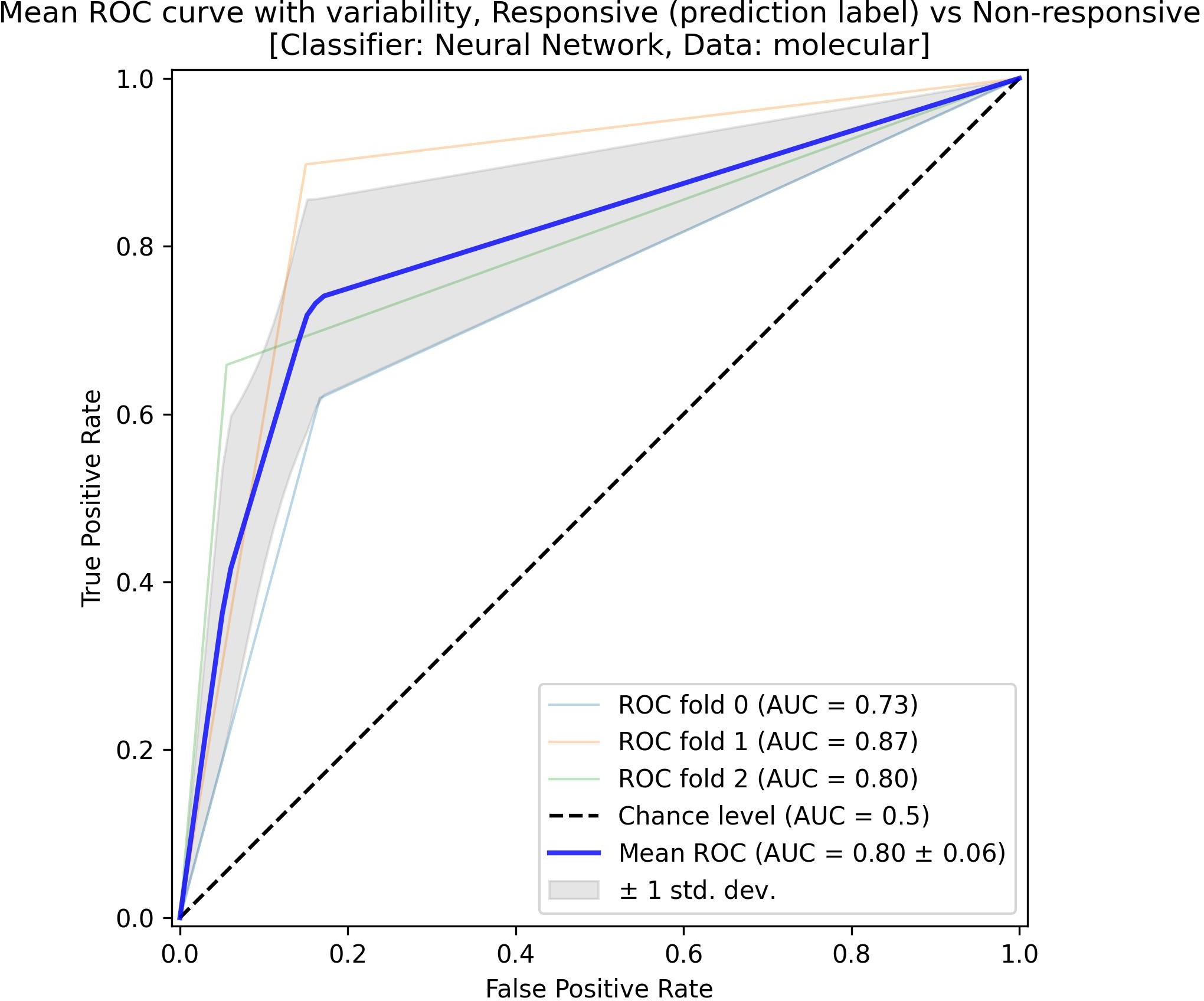


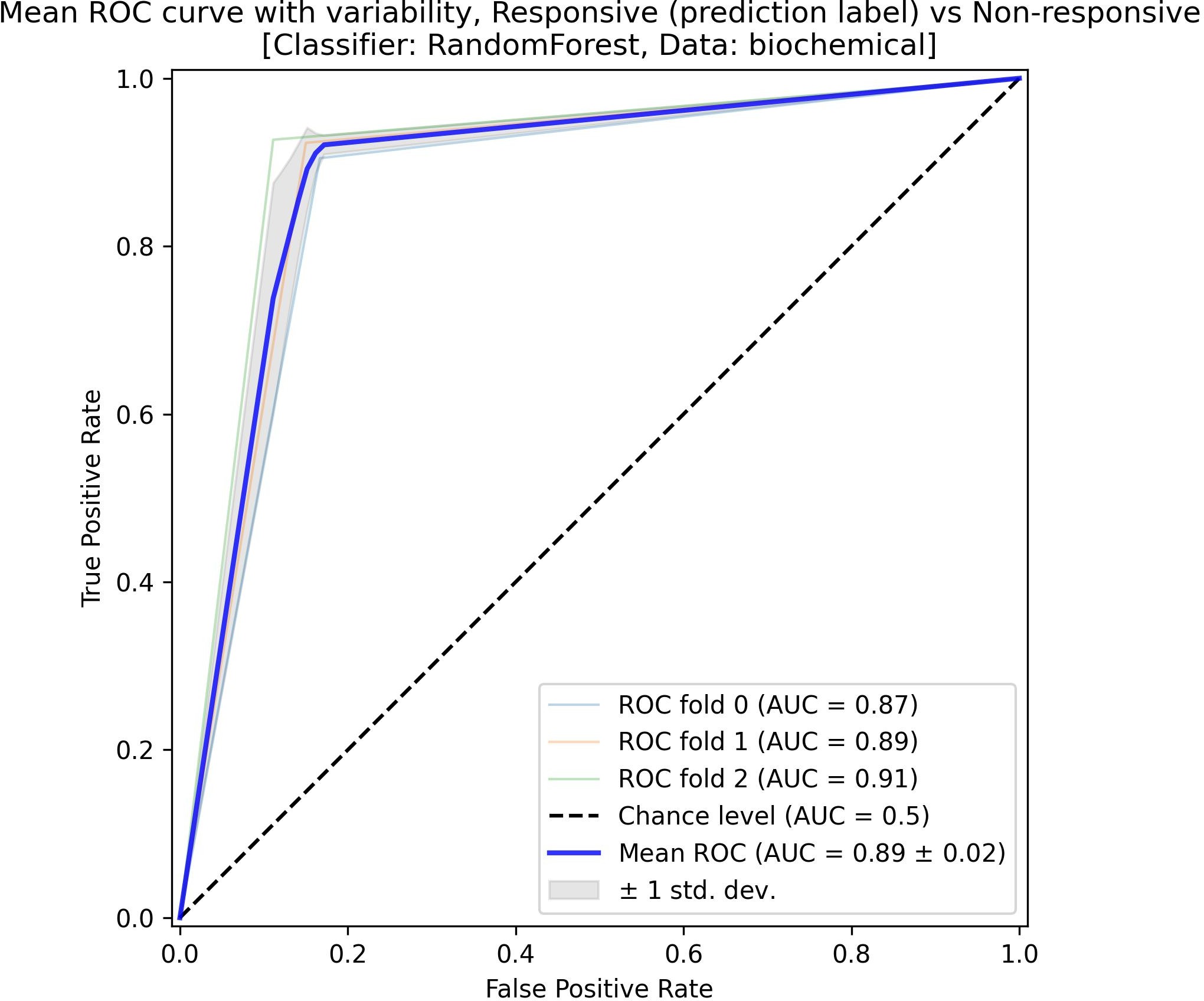


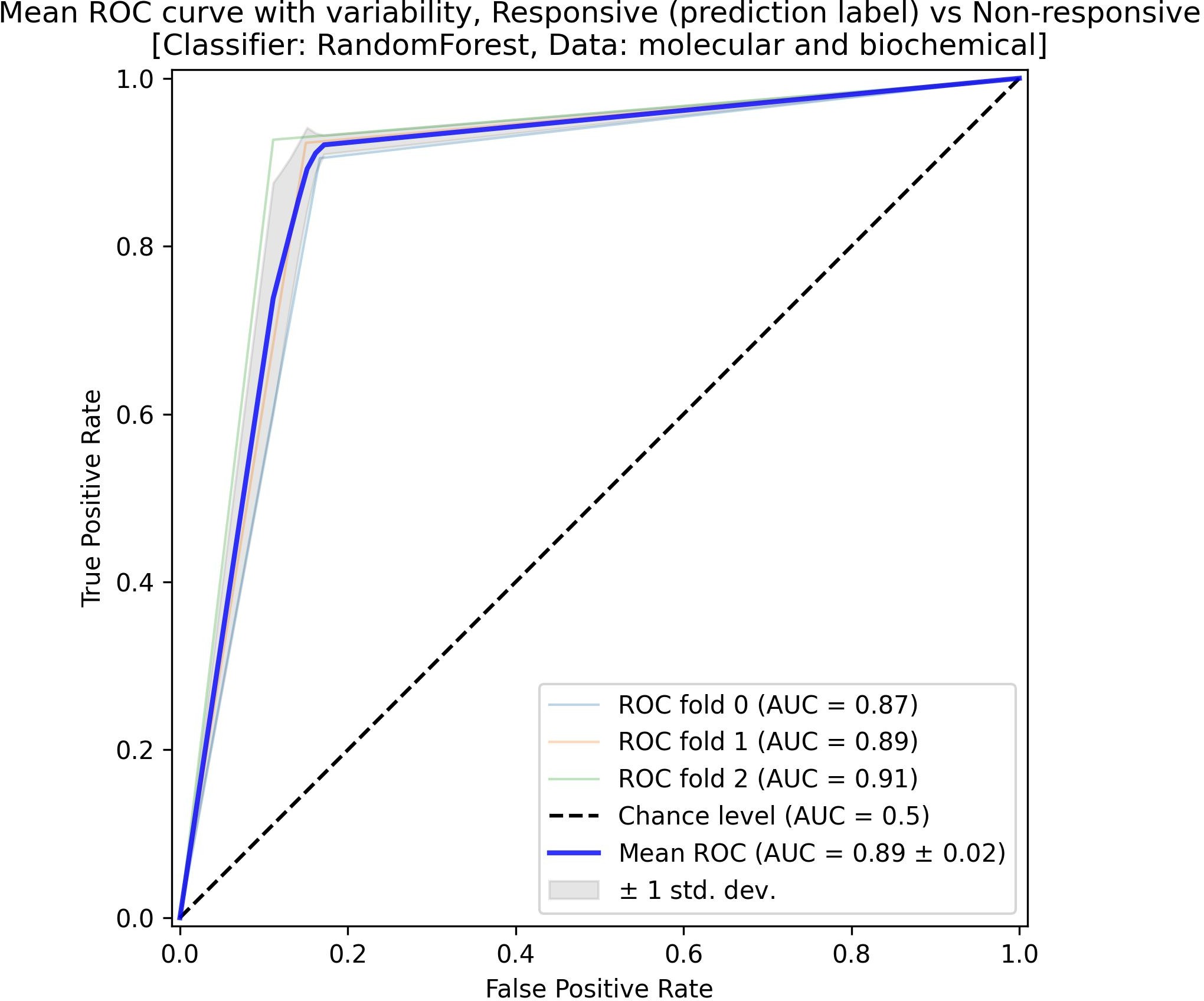


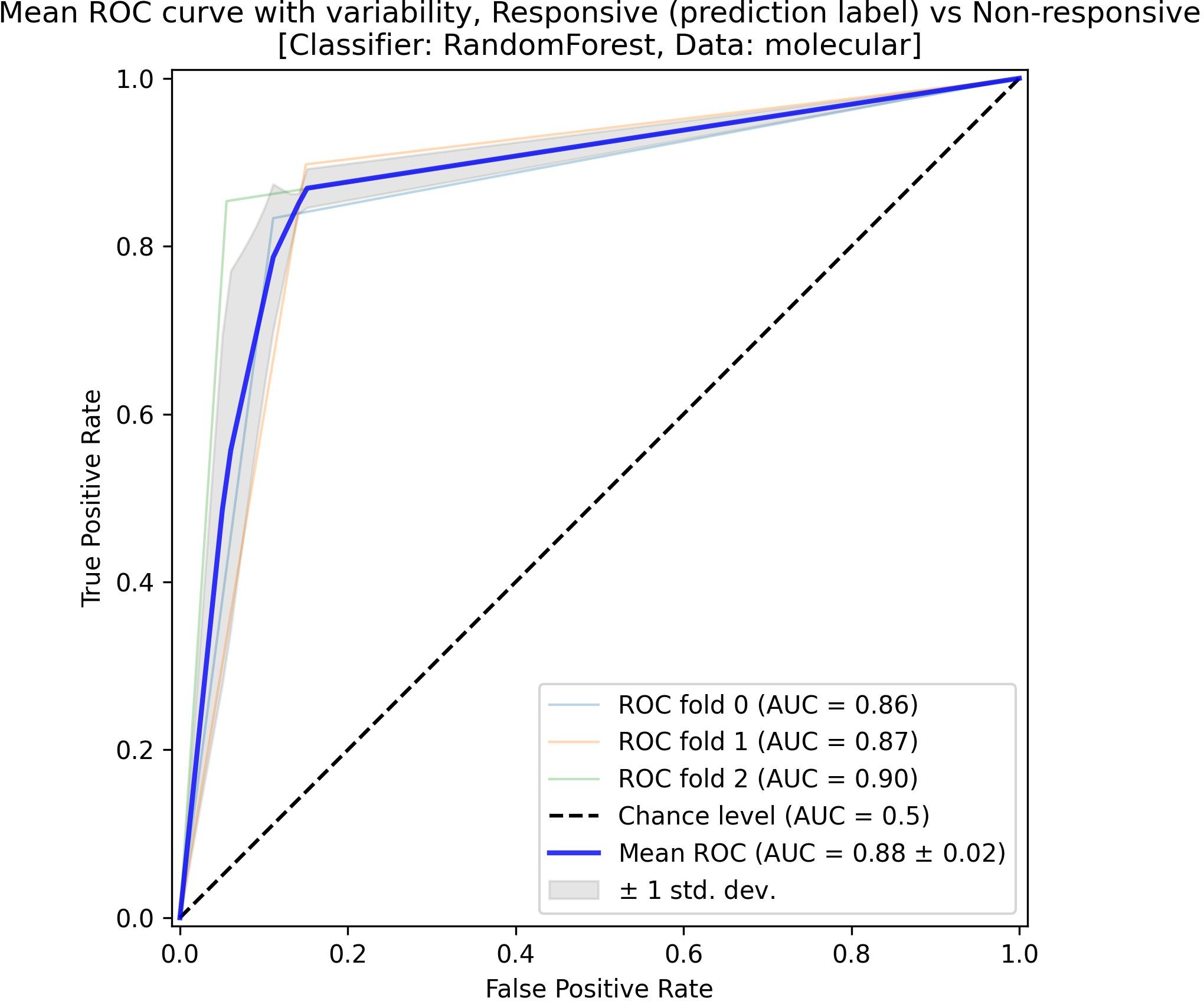


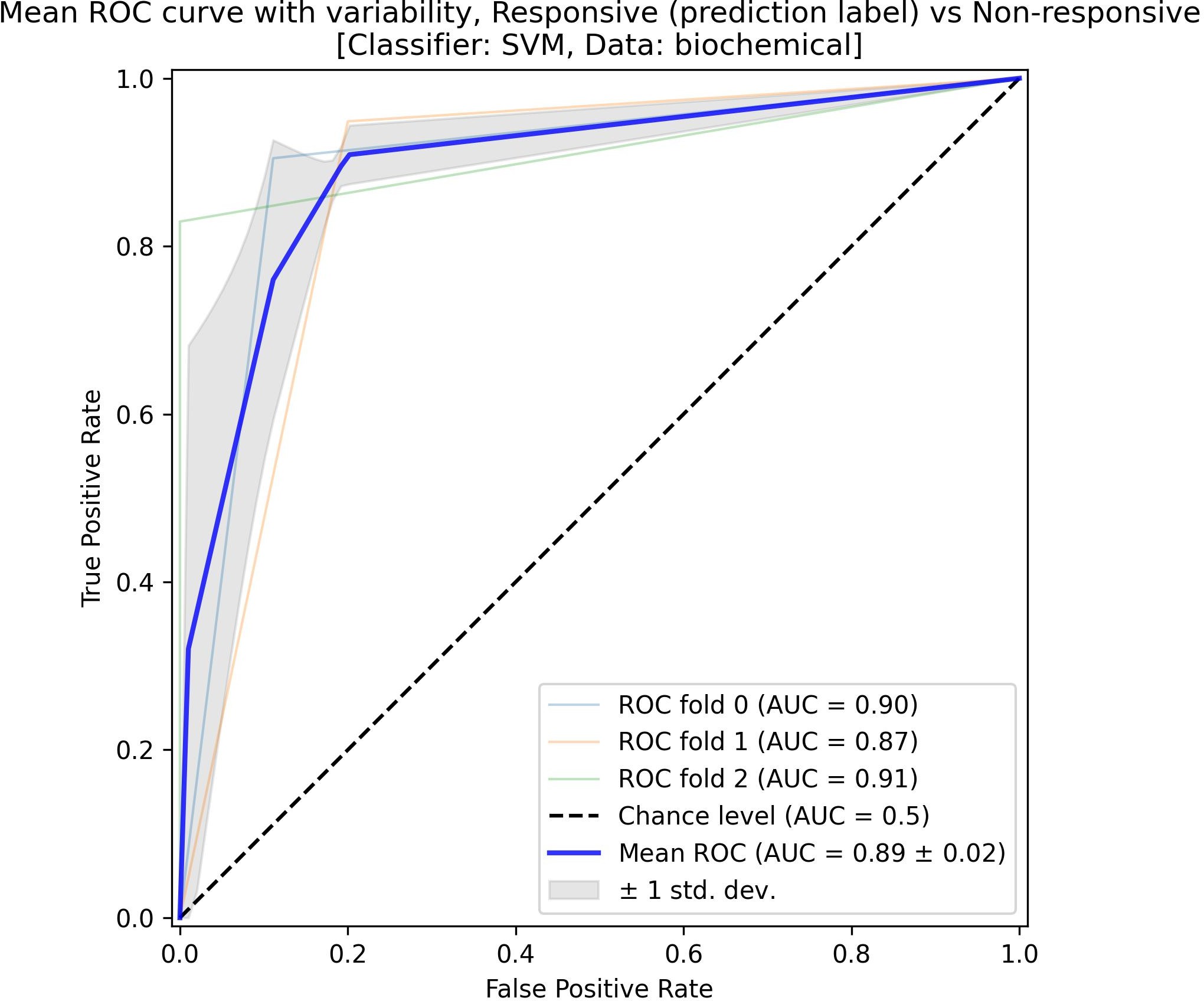

Supplement: Supplementary file 8 — Supplementary Material 8. [file 41598_2025_18620_MOESM8_ESM.docx]
